# Cortical morphometric gradients reveal molecular and cognitive underpinnings of bipolar disorder

**DOI:** 10.1017/S0033291725102705

**Published:** 2025-12-18

**Authors:** Rui Wang, Jiajun Xu, Fei Li, Xiaoqi Huang, Chunchao Xia, Su Lui, Qiyong Gong, Huaiqiang Sun

**Affiliations:** 1 Department of Radiology, Institution of Radiology and Medical Imaging, West China Hospital of Sichuan University, China; 2 Mental Health Center, West China Hospital, Sichuan University, China; 3 Department of Radiology, West China Hospital, Sichuan University, China; 4 Xiamen Key Lab of Psychoradiology and Neuromodulation, West China Hospital of Sichuan University, China

**Keywords:** bipolar disorder, cognitive-behavioral processes, MIND network gradients, neurotransmitter systems, transcriptomics

## Abstract

**Background:**

Structural brain alterations in bipolar disorder (BD) have been widely reported, yet the hierarchical organization of cortical morphometric networks and their molecular and cognitive underpinnings remain unclear.

**Methods:**

We applied the morphometric inverse divergence (MIND) network approach to structural MRI data from 49 BD patients and 119 healthy controls. Principal MIND gradients were derived using diffusion map embedding, followed by multiscale analyses linking gradient alterations to neurotransmitter systems, cognitive-behavioral domains, and transcriptomic profiles from the Allen Human Brain Atlas. Validation was performed in three independent, cross-scanner, cross-race, and cross-age validation datasets.

**Results:**

Bipolar disorder patients showed significant principal gradient alterations in the left rostral middle frontal and lateral occipital cortices, with network-level decreases in the ventral attention and motor networks and increases in frontoparietal and visual networks. Gradient alterations spatially correlated with acetylcholine (VAChT) and GABA (GABA_A/BZ_) systems, and were associated with cognitive processes involving executive control and visual attention. Transcriptomic analyses identified gene sets enriched for BD-related GWAS loci, expressed predominantly in excitatory and inhibitory neurons, astrocytes, and oligodendrocytes, with preferential enrichment in cortical layers III-IV and developmental windows spanning early fetal to young adulthood.

**Conclusions:**

These findings reveal disrupted hierarchical cortical organization in BD and link macroscale morphometric alterations to specific neurotransmitter systems and transcriptional architectures. The MIND gradient emerges as a potential biomarker bridging structural disruptions with molecular and cognitive mechanisms in BD.

## Introduction

Bipolar disorder (BD) is a severe psychiatric illness characterized by recurrent episodes of depression and mania or hypomania, leading to substantial functional and societal burden (Nierenberg et al., 2023; Oliva et al., 2025; Young & Juruena, 2021). Despite extensive research, the neurobiological mechanisms underlying BD remain incompletely understood, hindering progress in biomarker development and treatment stratification. Structural brain abnormalities, particularly in prefrontal and temporal regions, have been consistently reported using voxel-based (Ashburner & Friston, 2000; Volle, Gonen-Yaacovi, de Lacy Costello, Gilbert, & Burgess, 2011; Yongfeng Yang et al., 2022) and surface-based morphometry (Dale, Fischl, & Sereno, 1999; Hanford, Nazarov, Hall, & Sassi, 2016; Hibar et al., 2018; Tu, Chang, Kuan, Chen, & Su, 2024). However, most studies rely on univariate analyses of individual cortical features, which may fail to capture the network-level organization of the cortex and its relevance to BD pathophysiology.

Network-based approaches provide a framework to quantify cortical organization beyond isolated features. Morphometric similarity networks (MSN) integrate multiple morphometric indices to capture structural correlations across regions and have revealed alterations in various neuropsychiatric disorders (Del Casale et al., 2025; Janssen et al., 2025; Li et al., 2021; Lu et al., 2024; Park, Kim, & Lee, 2023; Tan et al., 2025; Tranfa et al., 2025; Wu et al., 2025; Xu et al., 2025; Zheng, Zhao, Yang, Guo, & Initiative, 2025). Yet, MSN approaches are constrained by reducing rich vertex-level data to region-wise averages and assuming standardized distributions. To overcome these limitations, the morphometric inverse divergence (MIND) method was recently proposed, which computes similarity using the divergence of vertex-level morphometric distributions (Sebenius et al., 2023). MIND networks demonstrate superior reliability and stronger correspondence with cortical cytoarchitecture, offering a promising tool to probe individual differences in cortical organization.

A complementary development in network neuroscience is the use of gradient analysis, which reduces high-dimensional connectivity features into low-dimensional components reflecting hierarchical brain organization (Bajada et al., 2020; Hong et al., 2020; Vos de Wael et al., 2020). Gradients derived from functional or structural connectomes consistently map a sensory-to-transmodal axis, providing insights into macroscale organization (Y. He et al., 2025; Huntenburg, Bazin, & Margulies, 2018; Margulies et al., 2016; Smallwood et al., 2021). Recent studies have applied gradient approaches to psychiatric conditions such as depression (Xue et al., 2023) and schizophrenia (Han et al., 2025), identifying disease-related disruptions in cortical hierarchy. However, it remains unclear whether BD is also associated with alterations in morphometric gradients, particularly those derived from the more reliable MIND framework.

Beyond characterizing structural hierarchy, it is essential to contextualize neuroimaging phenotypes with underlying molecular and cognitive mechanisms. The integration of neuroimaging with neurotransmitter distributions maps, meta-analytic cognitive activation profiles, and transcriptomic atlases (e.g. Allen Human Brain Atlas) has recently emerged as a powerful strategy to link macroscale abnormalities with neurochemical systems, behavioral domains, and genetic risk factors (Cui et al., 2023; Hansen & Misic, 2025; Huang et al., 2024; Yuchao Jiang et al., 2025; Jiang et al., 2024; Sun et al., 2024; Xu et al., 2024; Zhao et al., 2025). This multiscale framework is particularly relevant for BD, a highly heritable disorder with established polygenic architecture (Mistry, Harrison, Smith, Escott-Price, & Zammit, 2018; Mullins et al., 2021) and converging evidence for neurotransmitter system involvement (Ji et al., 2023; Kaufman et al., 2009).

**Figure 1.**
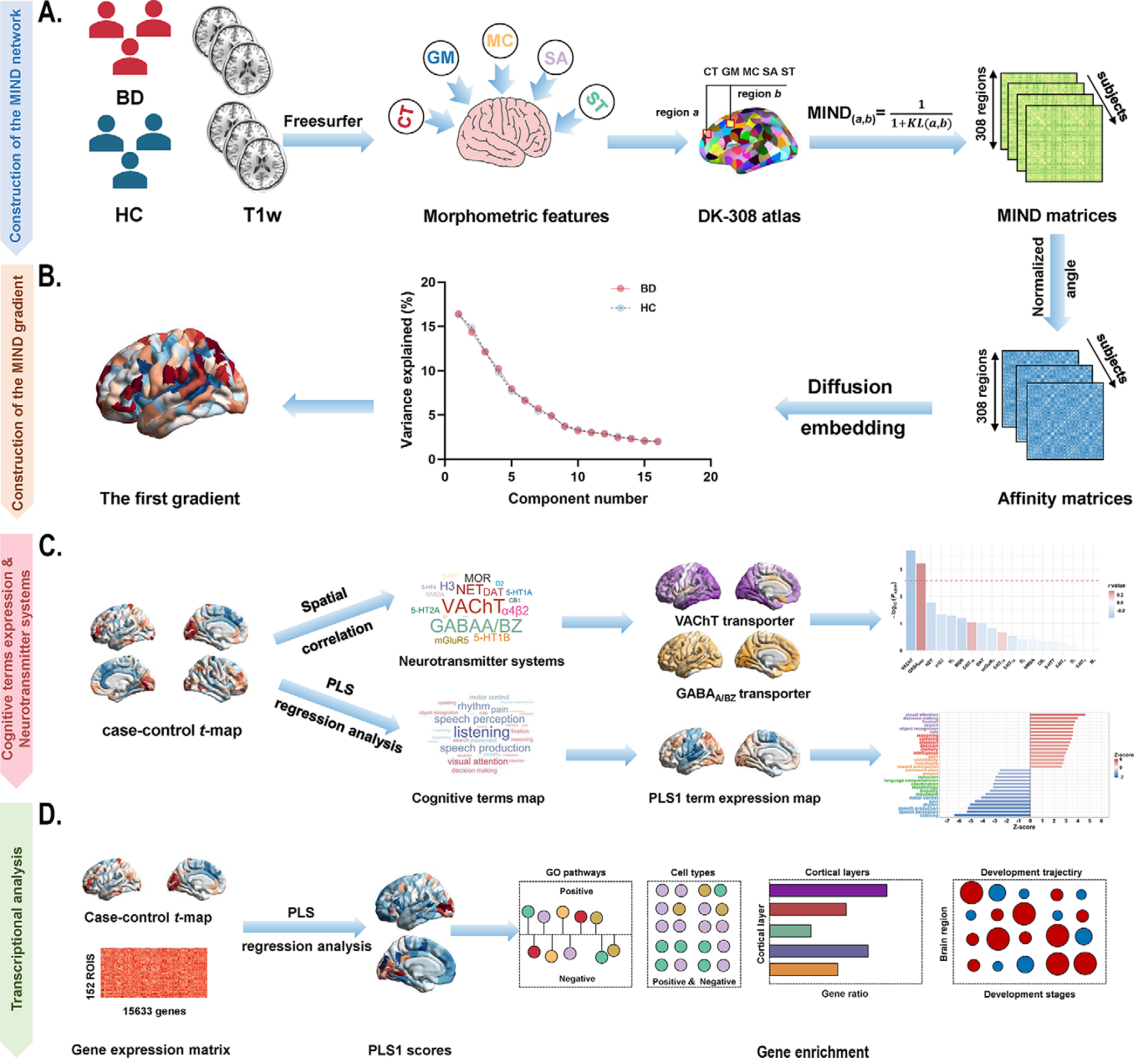
Schematic framework of the study design. (a) Construction of the MIND network in patients with BD and healthy controls. The MIND network construction process involves deriving vertex-level five structural features (CT, GM, MC, SA, and SD) from individual structural imaging maps. These features were standardized using *z*-scores across all vertices and then parcellated to create regional multivariate distributions. The MIND similarity statistic was calculated to generate the final MIND network, represented as a 308 × 308 matrix. The weighted node degree was then computed by averaging values across each column of the matrix. (b) Construction of the MIND gradient. The MIND network matrix was transformed into the affinity matrix by using the normalized angle method. We calculated the MIND network gradients using diffusion map embedding and focused on the principal gradient, which explained the greatest variance in connectivity. (c) Association of neurotransmitter systems and cognitive-behavioral processes with the principal MIND gradient case–control *t*-maps. Spatial relationships between the principal MIND gradient case–control *t*-maps and maps of neurotransmitter receptors or transporters were examined using Pearson’s correlation with the spin permutation tests. PLS regression analysis was applied to explore relate disparities in the principal MIND gradient identified by paired *t*-tests (response variables, represented by the paired *t* value) to cognitive functions (predictor variables, represented by the 125 cognitive-behavioral processes terms). (d) Transcriptional analysis workflow using AHBA gene expression data and PLS regression to identify genes associated with gradient alterations, followed by gene enrichment analyses. Abbreviations: AHBA, Allen Human Brain Atlas; BD, bipolar disorder; CT, cortical thickness; DK, Desikan-Killiany; GABA_A/BZ_, gamma-aminobutyric acid A/BZ; GLM, General linear model; GM, gray matter; GO, Gene Ontology; MC, mean curvature; MIND, Morphometric Inverse Divergence; PLS, partial least squares; SA, surface area; SD, sulcal depth; VAChT, vesicular acetylcholine transporter.

In this study, we aimed to investigate whether BD is associated with alterations in the principal MIND network gradient and to determine how these alterations relate to neurotransmitter systems, cognitive-behavioral processes, and gene expression profiles. Using a publicly available BD dataset, validated across independent samples, we combined morphometric gradient analysis with neurotransmitter systems, cognitive ontology frameworks, and transcriptomic decoding. We hypothesized that BD patients would exhibit heterogeneous gradient alterations, which would align with specific neurotransmitter receptors or transporters, cognitive functions, and BD-related genetic signatures. A schematic overview of the study framework is presented in Figure 1.

## Materials and methods

### Participants

The study comprised a primary analysis dataset and three independent cross-scanner, cross-race, and cross-age validation datasets. Primary analyses utilized the Consortium for Neuropsychiatric Phenomics (CNP) dataset, accessible through the OpenfMRI project (https://openneuro.org/datasets/ds000030/versions/1.0.0) (Poldrack et al., 2016). Validation samples were sourced from publicly available high-quality imaging datasets of healthy adults: Chinese Human Connectome Project (CHCP, https://www.Chinese-HCP.cn) (Ge et al., 2023), Multimodal Imaging and Connectome Analysis-Microstructure-Informed Connectomics (MICA-MICs, https://osf.io/j532r/) (Royer et al., 2022), and Southwest University Adult Lifespan Dataset (SALD, http://dx.doi.org/10.15387/fcp_indi.sald; Participants aged >60 years) (Wei et al., 2018). The CNP dataset originally included 179 right-handed adults (aged 21–50 years), comprising 49 individuals with BD and 130 healthy controls. BD diagnosis was established using the Diagnostic and Statistical Manual of Disorders, Fourth Edition-Text Revision (DSM-IV-TR) (Bell, 1994) and the Structured Clinical Interview for DSM-IV (First, 2002). Healthy participants were excluded for any lifetime psychiatric disorder, left-handedness, pregnancy, or other scanning contraindications. Validation datasets included individuals with no reported history of psychiatric or neurological disorders. Detailed participant information (ethics, informed consent, handedness, and inclusion/exclusion criteria) has been previously described (Ge et al., 2023; Poldrack et al., 2016; Royer et al., 2022; Wei et al., 2018; Zhu et al., 2021; Zhu, Zhang, Cai, Wang, & Yu, 2020). Following quality control procedures to exclude participants with poor imaging quality, motion artifacts, or missing structural data, as well as thorough checks of the preprocessed neuroimaging data to identify and correct errors in segmentation and surface reconstruction (Supplementary method 1). The final sample comprised 49 individuals with BD (21 females) and 119 healthy controls (57 females) from CNP, 353 healthy individuals (186 females, age range: 18–79 years, mean age: 33.98 ± 18.12) from CHCP, 50 healthy individuals (23 females, age range: 15–45 years, mean age: 29.54 ± 5.62) from MICA-MICs, and 95 healthy individuals (55 females, age range: 61–80 years, mean age: 67.95 ± 5.85) from SALD. Demographic details are provided in Table 1 and Supplementary Table S1.

**Table 1.** Demographic of the CNP dataset

Characteristics	BD (*n* = 49)	HC (*n* = 119)	Statistic	*P* value
Age (years)	35.29 ± 9.03	31.19 ± 8.63	*t* = 2.76	*P* < 0.01^a^
Gender (F/M)	21/28	57/62	*χ*^2^ = 0.3548	0.5514^b^
Education (years)	14.55 ± 1.96	15.13 ± 1.63	*t* = −1.99	0.0484^a^
TIV (cm^3^)	1526.12 ± 180.25	1519.15 ± 197.14	*t* = 0.21	0.8313^a^

a The *P* value is obtained by Chi-square test.

b The *P* value is obtained by two-sample *t*-test.

Age, education, and TIV are expressed as mean ± standard deviation.

Abbreviations: BD: bipolar disorder; F: female; HC: healthy controls; M: male; TIV: total intracranial volume.

### High-resolution structural MRI data acquisition scheme and preprocessing

For the CNP dataset, participants were scanned using two 3.0-Tesla Siemens Trio scanners, located at the Ahmanson-Lovelace Brain Mapping Center and the Staglin Center for Cognitive Neuroscience at UCLA, and the validation samples were acquired using 3.0-T Siemens Prisma or Trio scanners. Details of the acquisition parameters for all three datasets are provided in Supplementary Table S2 of the Supplementary Materials. High-resolution T1-weighted structural scans were preprocessed using the recon-all pipeline in *FreeSurfer* (version: 6.0.1; http://surfer.nmr.mgh.harvard.edu/) (Fischl, 2012), an automated brain segmentation process including: (i) skull stripping; (ii) tissue segmentation; (iii) hemispheric and subcortical structure segmentation; (iv) reconstruction of white matter and pial surfaces (Hedges et al., 2022).

### Construction of the MIND network

To minimize the influence of variability in parcel sizes, the cortical surface was parcellated into approximately equal-sized, spatially contiguous regions (~5 cm^2^) using a backtracking algorithm (Romero-Garcia, Atienza, Clemmensen, & Cantero, 2012), based on the 68 cortical regions in the Desikan–Killiany (DK) atlas (Desikan et al., 2006). Cortical regions were then parcellated into 308 spatially contiguous regions (Li et al., 2024; Morgan et al., 2019; Seidlitz et al., 2018) (Table 1 in the Supplementary file). This standard surface underwent transformation from standard space into each participant’s individual space. Each vertex of the individual surface was characterized by five cortical morphological measures, including cortical thickness (CT), gray matter (GM) volume, surface area (SA), mean curvature (MC), and sulcal depth (SD) through the recon-all pipeline in *FreeSurfer* (Sebenius et al., 2023). Based on the aforementioned five structural features, each measure was *z*-score normalized across all cortical vertices. The standardized data were then aggregated within a predefined 308-region parcellation to generate a regional multivariate distribution. Subsequently, the MIND similarity statistic was calculated by applying a transformation to the Kullback–Leibler (KL) divergence between the multivariate morphometric distributions of each pair of cortical regions, resulting in a symmetric similarity matrix with values bounded between 0 and 1, which yielded a 308 × 308 MIND matrix for each participant (Figure 1 and Supplementary Method 2) (Sebenius et al., 2023). Regional MIND values were determined by calculating the average weighted edge for each of the 308 cortical nodes, with no thresholds applied.

### Calculation of MIND gradients

The MIND gradients were calculated using the *BrainSpace* toolbox (Vos de Wael et al., 2020). The calculation pipeline proceeded as follows: First, consistent with previous studies (Chen et al., 2025; Hong et al., 2019; Jiang, Cui, et al., 2023; Margulies et al., 2016; Paquola et al., 2019; Shen et al., 2023; Song et al., 2023; Wang, Mo, et al., 2023; Yang et al., 2020; Yang et al., 2021), the top 10% MIND values were retained for each row in the MIND matrix (308 × 308). Second, pair-wise cortical region similarity was computed using the normalized angle kernel function, yielding a positive symmetric 308 × 308 affinity matrix that captures morphological profile similarities across regions. Third, MIND gradients were derived using diffusion map embedding (Coifman et al., 2005; Guell, Schmahmann, Gabrieli, & Ghosh, 2018; Margulies et al., 2016), a nonlinear dimensionality reduction technique that transforms high-dimensional data into lower-dimensional representations with components ranked by descending variance. The hyperparameter *α* in diffusion map embedding controls the influence of sampling density on the underlying manifold (*α* = 0 indicates maximal influence, whereas *α* = 1 indicates none). Following previous recommendations and the default setting of the *BrainSpace* toolbox (Dong et al., 2020; Guo et al., 2025; Margulies et al., 2016; Qu, Zhu, Wu, Xu, & Wang, 2024; Shen, Liu, Chen, & Qiu, 2025; Vos de Wael et al., 2020; Wang et al., 2025), we set *α* = 0.5, a value that optimally preserves global data relationships in the embedded space for brain connectivity analysis. Fourth, a group-level MIND gradient template was generated across all participants (both patients with BD and healthy controls). Finally, individual MIND gradients were calculated using identical parameters, with Procrustes rotation (Li et al., 2025; Sasse et al., 2024; Tan et al., 2024; Vos de Wael et al., 2020; Xia et al., 2022) applied to align individual gradients to the group template. Given that the principal MIND gradient (first gradient) explained the greatest structural connectivity variance, this study focused exclusively on this component.

### Comparison of the principal MIND gradient in patients with BD and healthy controls

General linear model (GLM) was employed to investigate between-group alterations of the regional principal MIND gradient while controlling for age, sex, education years, and age × sex interaction. To assess whether BD-related principal MIND gradient alterations preferentially involve specific functional networks and cytoarchitectonic types, we applied two widely used cortical parcellation schemes: the Yeo functional network (Yeo et al., 2011), which categorizes cortical regions into seven intrinsic functional networks, and the von Economo class (Economo, Koskinas, & Triarhou, 2008), which divides cortical regions into seven distinct classes based on cytoarchitectonic criteria (Supplementary Figure S3, and Table S3–S4). For each parcellation scheme, we calculated the mean principal MIND gradient score across regions within each network or class, and the GLM with the same covariates was applied. Multiple comparisons were corrected using the Benjamini-Hochberg false discovery rate (BH-FDR), with statistical significance set at *P* < 0.05.

### Association of neurotransmitter systems with the principal MIND gradient alterations

To further investigate the neurochemical basis of BD-related alterations in the principal MIND gradient, we correlated the case–control *t*-maps of the principal gradient with the cortical distributions of 19 neurotransmitter receptors and transporters derived from previously published nuclear imaging datasets of healthy individuals (Hansen et al., 2022). These maps summarized in Supplementary Table S8, cover major neurotransmitter systems, including acetylcholine (*α_4_β_2_*, M_1_, VAChT), cannabinoid (CB_1_), dopamine (DAT, D_1_, D_2_), gamma-aminobutyric acid (GABA_A/BZ_), glutamate (mGluR_5_, NMDA), histamine (H_3_), norepinephrine (NET), opioid (MOR), serotonin (5-HT_1A_, 5-HT_1B_, 5-HT_2A_, 5-HT_4_, 5-HT_6_, 5-HTT). Pearson’s correlations were computed between case–control *t*-maps and each neurotransmitter receptors or transporters across 308 cortical regions. Statistical significance was assessed using 10,000 spin permutation tests (Alexander-Bloch et al., 2018) (Supplementary method 5) with Bonferroni correction applied for multiple comparisons across the 19 neurotransmitter systems.

## Excitatory/inhibitory ratio

Excitatory–inhibitory (*E*/*I*) ratio was calculated across 308 cortical regions as the ratio of the normalized (*z*-scores) average density of excitatory receptors to that of inhibitory receptors (Hansen, Shafiei, Markello, et al., 2022; Hansen, Shafiei, Vogel, et al., 2022; Xia et al., 2025). Excitatory receptors include: *α_4_β_2_*, M_1_, D_1_, mGluR_5_, NMDA, 5-HT_2A_, 5-HT_4_, and 5-HT_6_. Inhibitory receptors include: CB_1_, D_2_, GABA_A/BZ_, H_3_, MOR, 5-HT_1A_, and 5-HT_1B_.

### Association of cognitive-behavioral processes with the principal MIND gradient alterations

To characterize the relationship between cognitive-behavioral processes and the principal MIND gradient alterations, we utilized Neurosynth (Lu et al., 2022) (https://neurosynth.org/), an automated, meta-analytic platform containing activation maps for 1,335 behavioral terms across diverse cognitive and affective functions. To minimize selection bias, we selected 125 cognitive-behavioral terms from the Cognitive Atlas (https://cognitiveatlas.org/) (Poldrack et al., 2011), a comprehensive neurocognitive taxonomy encompassing umbrella concepts (attention, emotion), specific processes (visual attention, episodic memory), behaviors (eating, sleep), and emotional states (fear, anxiety). The complete term list is provided in Supplementary Table S10. Neurosynth coordinates for each term were parcellated using the DK-308 atlas, yielding probabilistic measures representing how regional activity fluctuations align with psychological processes. Partial least squares (PLS) regression (Abdi & Williams, 2013; Liu et al., 2022; Mihalik et al., 2022; Poldrack et al., 2011) was applied to examine the relationships between cognitive-behavioral processes and alterations in the principal MIND gradient. The regression model treated the 125 cognitive-behavioral terms (308 regions × 125 terms) as predictor variables and principal MIND gradient case–control *t*-values (308 regions × 1) as the response variable. Of the extracted components (*n* = 20), the first component (PLS1) was the primary focus of our analysis, as it accounted for the greatest proportion of variance across components. Statistical significance of the variance explained by PLS1 was verified using 10,000 spin permutation tests of the response variables to generate a null distribution for comparison, with a threshold of *P* < 0.05. Furthermore, we computed normalized weight (*Z*-scores) using a bootstrapping method (10,000 iterations) to further assess the significance of each term’s contribution to the components, and all terms were ranked based on their contributions to the PLS component. Statistically significant terms corrected by BH-FDR method were retained (*P* < 0.05).

### Transcriptomic data and preprocessing

Brain gene expression data were obtained from the Allen Human Brain Atlas (AHBA) database (https://human.brain-map.org/static/download) (Hawrylycz et al., 2012), comprising post-mortem samples from six adult donors (Supplementary Table S12). Since all six donors provided left hemisphere data while only two included right hemisphere samples, we restricted our analysis to left hemisphere transcriptional profiles. Data preprocessing was performed using *abagen* (version: 0.1.4, https://github.com/rmarkello/abagen) (Markello et al., 2021), a Python-based toolbox that provides a standardized workflow widely adopted in transcription-neuroimaging studies (Ji et al., 2023; Liu, Abdellaoui, van Wingen, & Verweij, 2024; Markello et al., 2021; Meng et al., 2022; Shafiei et al., 2023; Zhang et al., 2025). The preprocessing pipeline included: (i) updating probe-to-gene annotations, (ii) intensity-based filtering, (iii) representative probe selection, (iv) sample-to-region matching, (v) missing data imputation, (vi) cross-sample and cross-gene normalization, (vii) within-region expression aggregation, and (viii) filtering for stable and consistent genes. This workflow generated a final expression matrix of 152 left hemisphere regions × 15,632 genes for subsequent analyses.

### Transcription-neuroimaging association analysis

We employed PLS regression (Abdi & Williams, 2013; Chen et al., 2025; Jiang et al., 2025; Jiang et al., 2024; Liu et al., 2022; Mihalik et al., 2022; Song et al., 2023) to establish the connection between gene expression and regional changes in the principal MIND gradient. The regression model used *z*-score normalized gene expression data (152 cortical regions × 15,632 genes) as predictor variables and the corresponding principal MIND gradient case–control *t* values (152 cortical regions × 1) as the response variable. Resultant PLS components represent linear combinations of weighted gene expression values, ranked by their explained variance between predictor and response variables. As mentioned previously, we focused primarily on the first component (PLS1), which accounted for the greatest proportion of variance. Statistical significance of the PLS components was assessed using 10,000 spin permutation tests to generate null distributions (*P* < 0.05). We further evaluated gene-level contributions using bootstrapping (10,000 iterations) to compute normalized weights (*Z*-scores), retaining genes that remained significant after BH-FDR correction (*P* < 0.05). To examine the relationships between bipolar disorder-related genes and principal MIND gradient alterations, we analyzed three BD-associated genes from the Allen Human Brain Atlas database (https://help.brainmap.org/download/attachments/2818165/HBA_ISH_GeneList.pdf?version=2&modificationDate=1614977648535&api=v2) (Zeng et al., 2012) (Supplementary Table S13). Finally, gene set enrichment analyses were performed on significant genes with positive or negative weights (PLS1+/−) using the *MAGMA* toolbox (De Leeuw, Mooij, Heskes, & Posthuma, 2015) (version 1.10, https://cncr.nl/research/magma/) to determine whether PLS1-derived genes were enriched for BD risk genes identified by a large-scale, multi-cohort genome-wide association study (GWAS) (Mullins et al., 2021) (Supplementary Method 3).

### Enrichment analyses

Genes with positive weights on the first PLS component (PLS1+) were subjected to comprehensive functional enrichment analyses. Using the *Metascape* platform (https://metascape.org/gp/index.html#/main/step1), we examined functional annotations of PLS1+ genes to identify biological pathways related to Gene Ontology (GO) terms and human diseases from the DisGeNET database (Zhou et al., 2019). To further investigate cellular and cortical layer specificity associated with the principal MIND gradient, we conducted cell type and cortical layer enrichment analyses. In addition, the cell-type specific expression analysis (CSEA) tool (http://doughertytools.wustl.edu/CSEAtool.html) (Dougherty, Schmidt, Nakajima, & Heintz, 2010) was employed to perform developmental gene expression enrichment analysis to investigate developmental time windows across brain regions. All enrichment analyses used a significance threshold of *P* < 0.05 after BH-FDR correction. Detailed procedures for all enrichment analyses can be found in Supplementary Method 4.

### Multiple linear regression analysis

Multiple linear regression model was constructed to examine the genetic and neurotransmitter systems’ contributions to the observed principal MIND gradient alterations in BD. Regional changes in the principal MIND gradient (case–control *t* value) served as the response variable, with genetic profiles (PLS1 scores) and neurotransmitter transporter-related profiles (transporter density and *E*/*I* ratio) as predictors. The relative importance of each predictor was quantified using the *relaimpo* package (version:2.2.7) (Grömping, 2007) in R, followed by a bootstrapping procedure (1,000 iterations) to estimate each predictor’s relative contribution (%) to the model’s total explained variance (*R*^2^).

### Validation analyses

Several validation analyses were conducted to verify the robustness of our findings. First, we addressed potential confounding by total intracranial volume (TIV), a commonly used covariate in neuroimaging studies (Crowley et al., 2018; Malone et al., 2015), by regressing out TIV and assessing the stability of case–control *t*-maps in the principal MIND gradient. We then examined the effect of different connectivity thresholds on our results by re-analyzing data using the top 20% and 30% MIND values per row (compared to our standard 10% threshold), following established protocols (Jingyao Chen et al., 2025; Jiang, Sultan, et al., 2023; Shen et al., 2023; Song et al., 2023; Wang, Mo, et al., 2023; Wang, Royer, et al., 2023). To address potential sample size imbalance effects, we repeated analyses using a randomly-selected, size-matched subset of healthy controls. Cross-validation was performed using three independent, cross-race, cross-scanner, and cross-age datasets (CHCP, MICA-MICs, and SALD) to exclude sample-specific and age-specific effects and assess analytical reliability. Finally, given the established links between BD and specific genes, notably *CACNA1C* (Allen IV et al., 2024; Jiang, Sultan, et al., 2023; Owen, Bray, Walters, & O’Donovan, 2025; Yang, Zhu, Hui, & Sun, 2024) and five genes associated with somatostatin (*SST*) interneurons (Keon Arbabi et al., 2025; Pantazopoulos, Wiseman, Markota, Ehrenfeld, & Berretta, 2017), we tested the robustness of relationships between case–control differences and the transcriptional expression patterns of these targeted genes.

## Results

### Demographic characteristics

A total of 168 participants were ultimately retained in the primary analysis, including 49 patients with BD (21 females) and 119 healthy controls (57 females). Demographic for the primary analysis is detailed in Table 1. The demographic characteristics, including sex (chi-square test, *χ*^2^ = 0.35, *P* = 0.55), and TIV (two-sample *t*-test, *t* = 0.21, *P* = 0.83) exhibited no statistically significant differences between BD patients and HC.

### Case–control differences in the principal MIND gradient

**Figure 2.**
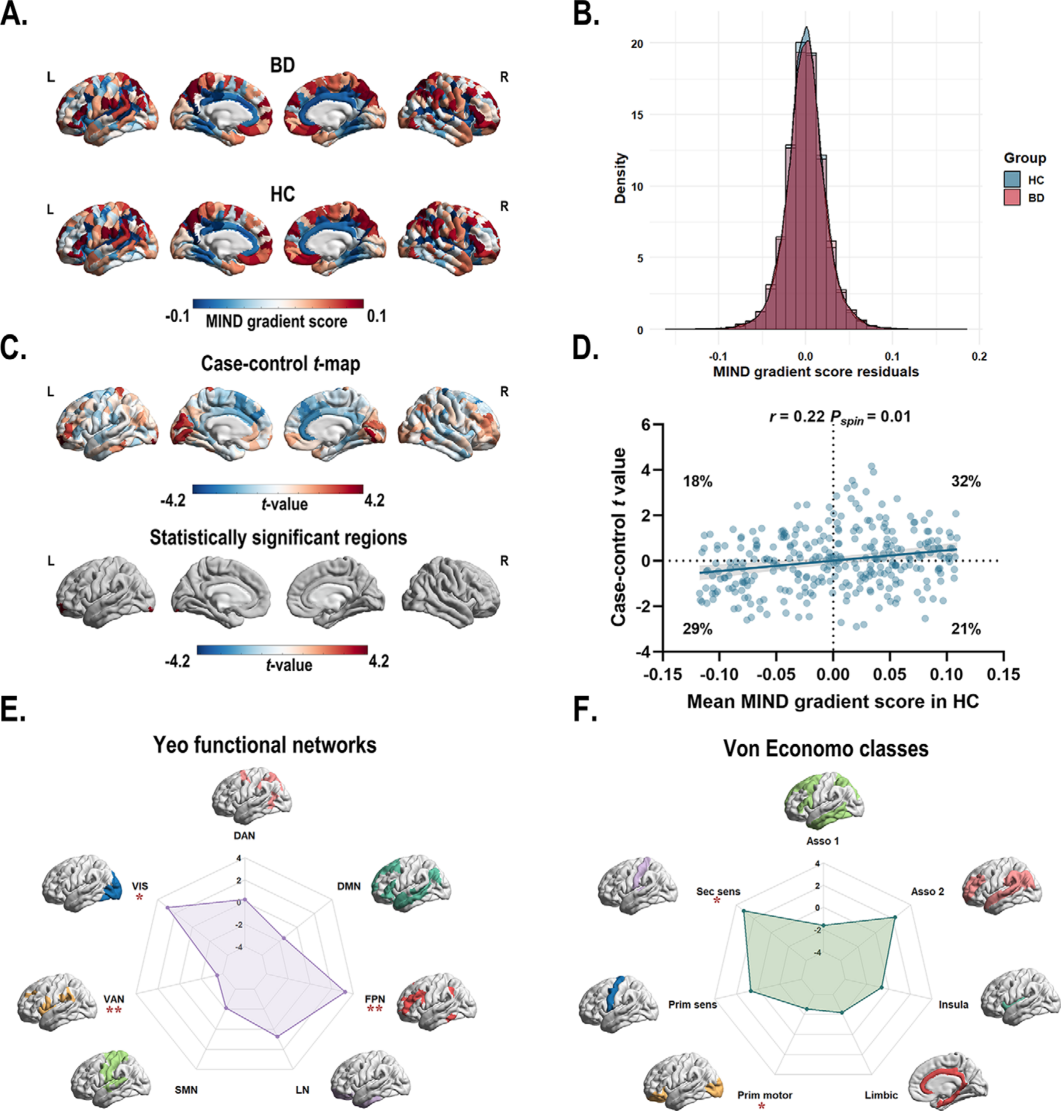
(a) The principal MIND gradient pattern in patients with BD and healthy controls. Regions with similar connectivity patterns show similar colors. (b) The histogram shows the distributions of mean principal MIND gradient scores in the BD and healthy controls while regressing out the effect of age, sex, education years, and age × sex interaction. (c) Case–control comparison of regional principal MIND gradient between BD patients and healthy controls, with BD > healthy controls shown in red. BD patients showed a significantly increased principal MIND gradient in the left lateral occipital cortex (part8), and the left rostral middle frontal (part4) regions. All *P* values survived after BH-FDR correction with *P* < 0.05. (d) The scatterplot of the mean regional principal MIND gradient scores in healthy controls and the case–control *t*-values. The case–control *t*-values showed a positive spatial correlation with the regional MIND values in healthy controls (*r* = 0.22, *P_spin_* = 0.01). The gray band indicates the 95% confidence interval. (e–f) Functional community-based *t*-values (left; Yeo functional networks) and cytoarchitecture-based *t*-values (right; von Economo classes) of the principal MIND gradient. *Indicates that the BH-FDR corrected *P* value <0.05, and ^**^ indicates that the BH-FDR corrected *P* value <0.01. Abbreviations: Asso1, association cortex1; Asso2, association cortex2; BD, bipolar disorder; BH-FDR, Benjamini–Hochberg false discovery rate; DAN, dorsal attention network; DMN, default mode network; FPN, fronto-parietal network; Insula, insular cortex; Limbic, limbic regions; LN, limbic network; MIND, Morphometric Inverse Divergence; Prim motor, primary motor cortex; Prim sens, primary sensory cortex; Sec sens, second sensory cortex; SMN, somato-motor network; VAN, ventral attention network; VIS, visual network.

A two-sample *t*-test (*t* = 7.30 × 10^−3^, *P* = 0.99) indicated similar variance explained across gradients for both BD and HC groups. The principal gradient accounted for 16% of the MIND network variance (Supplementary Figure S1). The principal gradient exhibited a similar pattern with higher gradient scores in the prefrontal (i.e. dorsolateral prefrontal cortex, medial prefrontal cortex, and ventrolateral prefrontal cortex), motor, parietal (i.e. inferior parietal lobule), and temporal cortices and lower scores in the somatosensory, occipital, and primary auditory cortices (Figure 2a). The spatial patterns of the 1–5 gradients are shown Supplementary Figure S2 in the Supplementary materials.

Using GLM with age, sex, education years, and age × sex interaction, region-wise comparisons revealed that BD patients exhibited significantly increased principal MIND gradient scores in the left lateral occipital cortex (part8) and the left rostral middle frontal (part4) regions compared to HC (Figure 2c and Supplementary Table S5). However, the overall distribution of mean principal MIND gradient scores showed no significant case–control difference (*P* = 0.11, two-sample Kolmogorov–Smirnoff test; Figure 2b). A significant positive spatial correlation (*r* = 0.22, *P_spin_* = 0.01) was observed between the mean regional MIND gradient in healthy controls and the case–control *t*-map (Figure 2d). This indicates that regions at both extremes of the MIND gradient exhibited larger case–control differences: areas with higher positive gradient scores in controls showed greater increases in BD patients, while those with higher negative scores demonstrated greater decreases. To examine functional and cytoarchitectural organization, we applied the Yeo functional network atlas (Yeo et al., 2011) (Supplementary Figure S3A) and von Economo cytoarchitectural atlas (Economo et al., 2008) (Supplementary Figure S3B). Within functional networks, BD patients showed significantly decreased principal MIND gradients in the ventral attention network and significantly increased gradients in the fronto-parietal and visual networks (*P* < 0.05, BH-FDR corrected; Figure 2e, Supplementary Figure S4A, Supplementary Table S6). At the cytoarchitectural level, BD patients exhibited significantly decreased gradients in the primary motor cortex and increased gradients in the secondary sensory cortex (*P* < 0.05, BH-FDR corrected; Figure 2f, Supplementary Figure S4B, Supplementary Table S7).

### Association of neurotransmitter systems with the principal MIND gradient alterations

**Figure 3.**
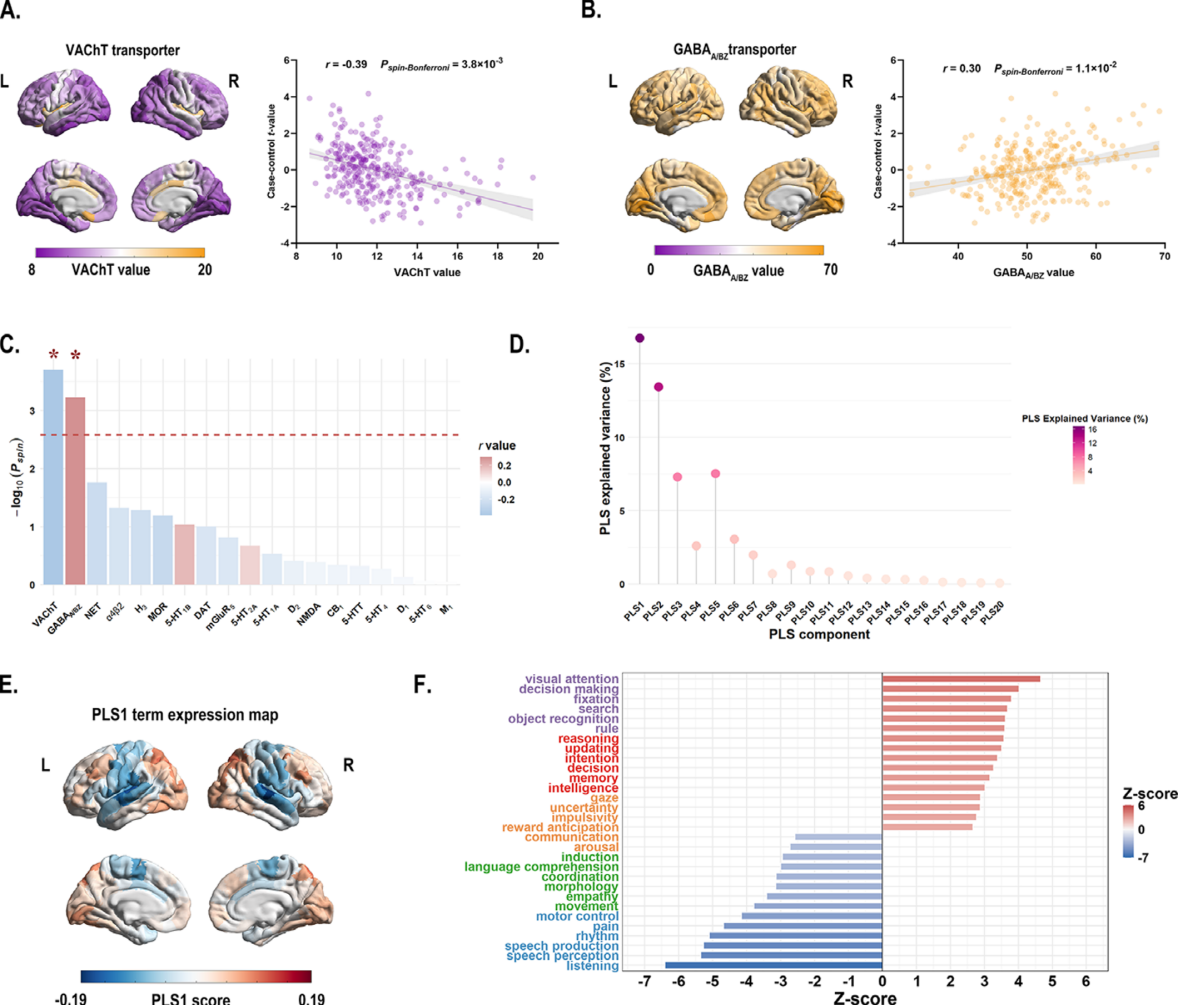
(a) Left: The distribution of VAChT transporter across 308 cortical regions. Right: Scatterplot showing the relationship between regional VAChT transporter values and the case–control *t*-values. The case–control *t*-values exhibited a significant negative spatial correlation with the regional VAChT transporter values (*r* = −0.39, *P_spin-Bonferroni_* = 3.8 × 10^−3^). The gray band indicates the 95% confidence interval. (b) Left: The distribution of GABA_A/BZ_ transporter across 308 cortical regions. Right: Scatterplot showing the relationship between regional GABA_A/BZ_ transporter values and the case–control *t*-values. The case–control *t*-values showed a significant positive spatial correlation with the regional GABA_A/BZ_ transporter values (*r* = 0.30, *P_spin-Bonferroni_* = 1.1 × 10^−2^). The gray band indicates the 95% confidence interval. All *P* values were evaluated through 10,000 spin-tests, followed by the Bonferroni method to account for multiple comparisons across 19 different transporter and receptor maps. (c) The bar plot displays the −log_10_ (*P_spin_*-value) for each transporter or receptor map. Asterisks (*) indicate significance after Bonferroni multiple comparisons correction (*P_spin-Bonferroni_* < 0.05). (d) The lollipop chart demonstrated that the significance of the variance explained by PLS1 was confirmed through permutation testing of the cognitive-behavioral processes terms and the principal MIND gradient differences. The PLS1 component significantly accounted for the total variance in the principal MIND gradient difference (*P_spin_* < 0.05), explaining 16.6% of the variance. The chart displays the explained variance for all 20 derived PLS components. (e) The PLS1 term expression map across cortical regions. (f) Cognitive-behavioral processes terms with the largest absolute Z scores, indicating their robust association with the principal MIND gradient difference survived after BH-FDR correction with *P* < 0.05. Abbreviations: *α_4_β_2_*, nicotinic acetylcholine receptors; BH-FDR, Benjamini-Hochberg false discovery rate; 5-HT, 5-hydroxytryptamine (serotonin); CB_1_, cannabinoid type 1; D, dopamine; DAT, dopamine transporter; GABA_A/BZ_, gamma-aminobutyric acid A/BZ; H_3_, histamine H_3_ receptor; mGluR_5_, metabotropic glutamate type 5; L, left; M_1_, muscarinic acetylcholine receptor M_1_; MOR, mu opioid receptor; NAT, noradrenaline transporter; NET, norepinephrine transporter; NMDA, N-methyl-D-aspartate receptor; PLS, partial least squares; R, right; VAChT, vesicular acetylcholine transporter.

Cross-region spatial correlation analyses revealed significant associations between principal MIND gradient alterations and specific neurotransmitter systems (permutation-based test, Bonferroni corrected *P* < 0.05). Specifically, the case–control *t*-map showed a negative association with acetylcholine VAChT transporter (*r* = −0.39, *P_spin-Bonferroni_* = 3.8 × 10^−3^) (Figure 3a and c, and Supplementary Table S9), and a positive association with GABA_A/BZ_ transporter (*r* = 0.30, *P_spin-Bonferroni_* = 1.1 × 10^−2^) (Figure 3b and c and Supplementary Table S9).

### Association of cognitive-behavioral processes with the principal MIND gradient alterations

In the PLS regression model that relate group differences in MIND gradient between BD patients and HC to 125 cognitive-behavioral terms, the PLS1 component explained 16.8% of the total variance in the principal MIND gradient case–control *t* value (*P_spin_ =* 0.012; Figure 3d). Terms showing significant negative correlations with principal MIND gradient alterations included ‘listening’, ‘speech perception’, and ‘speech production’ (*Z*-score < 0, *P* < 0.05, BH-FDR corrected; Figure 3f), while ‘visual attention’, ‘decision making’, and ‘fixation’ demonstrated positive correlations (*Z*-score > 0, *P* < 0.05, BH-FDR corrected; Figure 3f). Complete results are provided in Supplementary Table S11.

### Transcription-neuroimaging associations

**Figure 4.**
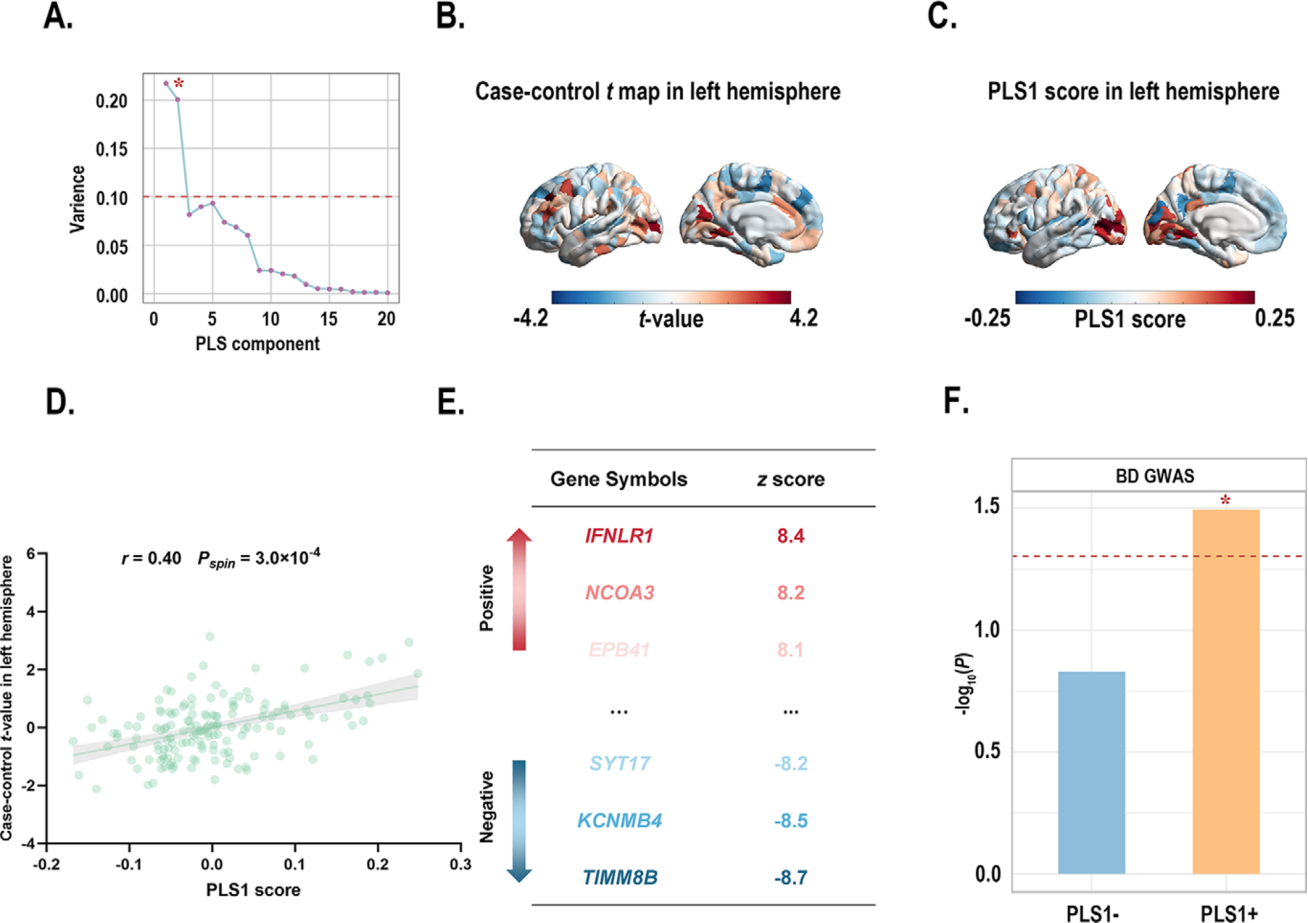
(a) Variance in case–control difference in the principal MIND gradient explained by the top 20 PLS components. Notably, the first component (PLS1) explained 21.70% of the variance, the highest among 20 PLS components, and was significantly greater than the random level controlling for spatial autocorrelation (*P_spin_* < 0.05). * indicates that the component meets the criteria. (b) The case–control *t*-maps of the regionally principal MIND gradient scores in the left hemisphere. (c) A weighted gene expression map of regional PLS1 scores in the left hemisphere. (d) Scatterplot showing the relationship between regional PLS1 scores and regional changes in the principal MIND gradient. The PLS1 scores showed a significant positive spatial correlation with the principal MIND gradient (*r* = 0.40, *P_spin_* = 3.0 × 10^−4^). The gray band indicates the 95% confidence interval. (e) Ranked PLS1 genes based on *Z* scores. (f) Gene set analyses of PLS1+/− gene lists for risk genes identified by GWAS. Asterisks (*) indicate *P*_pls1*+*_ < 0.05. Abbreviations: BD, bipolar disorder; GWAS, Genome-Wide Association Study; MIND, Morphometric Inverse Divergence; PLS, partial least squares.

PLS1 accounted for 21.70% of the variance in the principal MIND gradient case–control differences, considerably more than anticipated by chance (*P_spin_* = 0.019; Figure 4a). The distributions of the case *t*-map and PLS1 scores are presented (Figure 4b and c). Interestingly, the left rostral middle frontal (part 4) regions exhibited the highest PLS1 scores and *t*-values. We also found the PLS1 score was positively correlated with the case–control *t*-map (*r* = 0.40, *P_spin_* = 3.0 × 10^−4^; Figure 4d). A total of 4,500 genes were identified as significant contributors to PLS1 (*P* < 0.05, BH-FDR-corrected; Figure 4e). Of these, 2,332 genes with normalized positive PLS1 weights were classified as PLS1+, and 2,168 genes with normalized negative PLS1 weights were classified as PLS1−. Notably, 3 overlapping genes (identified between BD-related genes from the AHBA database and the background gene set) significantly contributed to PLS1 (Supplementary Table S13). Of these three genes, two were categorized as PLS1+ gene list and their expression profiles were also significantly positively spatially correlated with the case–control *t*-map (Supplementary Figure S6). To further investigate whether PLS1 derived genes (PLS1+/−) were enriched for BD risk genes identified by a large-scale, multi-cohort GWAS study, we performed gene set enrichment analyses using *MAGMA.* Our findings indicated that PLS1+ genes were significantly enriched for BD risk genes obtained from the GWAS (*P*_PLS1_*+* = 0.0322; Figure 4f), while PLS1− genes were not (*P*_PLS1−_ = 0.1482; Figure 4f). Consequently, we focused on the PLS1+ gene list for subsequent enrichment analyses.

### Enrichment analyses

**Figure 5.**
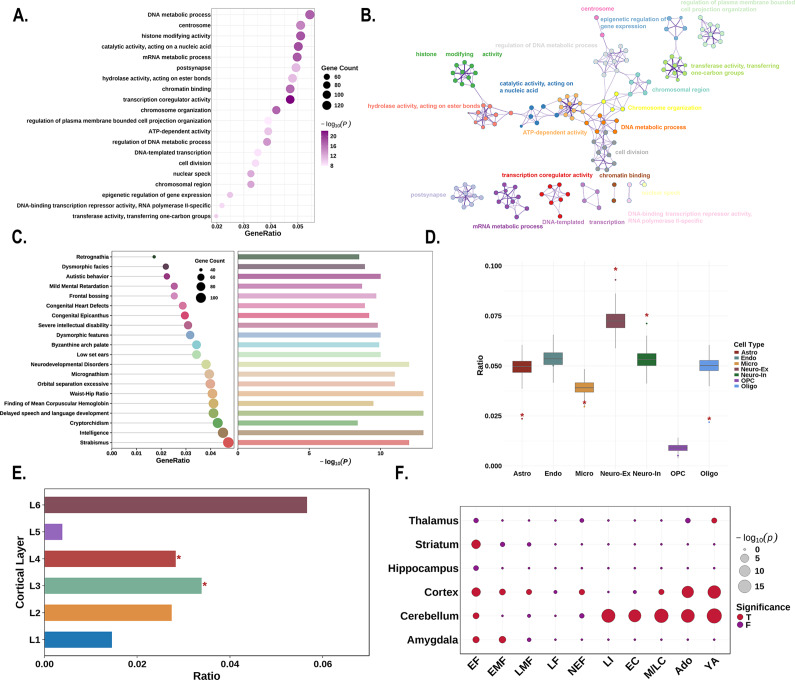
Enrichment analyses of the PLS1+ genes. (a) The bubble plot shows the GO functional annotations for the PLS1+ genes. The bubble size represents the number of overlapping genes between the PLS1+ gene list and each GO term (y-axis). The color bar represents the FDR corrected *P* value. (b) Metascape enrichment network visualization showing the intracluster and inter-cluster similarities of enriched pathways. Each pathway is shown by a node, where the node size is proportional to the number of input genes included in the pathway, and different colors correspond to different clusters. (c) The combined plot visualizes human diseases from the DisGeNET database annotated for PLS1+ genes. Left: The lollipop plot shows enrichment, where bubble size indicates the number of overlapping genes between the PLS1+ gene list and each human disease term (y-axis). Right: The bar plot depicts the statistical significance, with bar length representing the −log_10_ (*P*) value (FDR corrected *P* value), longer bars denote greater significance. (d) Cell type enrichment analysis of the PLS1+ gene list. The boxplots illustrate the ratio of genes in each gene set preferentially expressed in seven distinct cell types compared with a null model of randomly selected genes (based on 10,000 repetitions), with significant differences denoted by * *P* < 0.05. The boxplots depict the first, second (median), and third quartiles, while the dots indicate the real ratio, and small dots signify outliers. (e) Cortical layer enrichment analysis of the PLS1+ gene list. The barplot illustrates the ratio of PLS1+ genes preferentially expressed in six cortical layers, compared with a null model of randomly selected genes (based on 10,000 repetitions). Significant differences are indicated by an asterisk (* indicates that *P* < 0.05). (f) Developmental gene expression enrichment analysis of the PLS1+ gene list. The size of each bubble is inversely proportional to the BH-FDR corrected *P* value (or more commonly, proportional to the −log_10_ of the BH-FDR corrected *P* value), with larger bubbles representing greater statistical significance. The color of the bubbles indicates whether the PLS1+ genes are significantly enriched: red for significant enrichment and blue for no significant enrichment. Abbreviations: Ado, adolescence; BH-FDR, Benjamini–Hochberg false discovery rate; EC, early childhood; EF, early fetal; EMF, early/mid fetal; F, false; GO, gene ontology; LF, late fetal; LI, late infancy; LMF, late/mid fetal; M/LC, mid/late childhood; NEF, neonatal early infancy; PLS, partial least squares; T, true; YA, young adulthood.

For functional enrichment analysis, the PLS1+ genes showed significant enrichment in pathways related to gene expression and RNA metabolism, including ‘transcription coregulator activity’, ‘catalytic activity, acting on a nucleic acid’, ‘histone modifying activity’, and ‘mRNA metabolic process’ (Figure 5a, b). These genes were also enriched for human diseases and phenotypes associated with ‘delayed speech and language development’, ‘intelligence’, ‘neurodevelopmental disorders’, and ‘autistic behavior’ (Figure 5c).

Regarding cellular expression patterns, PLS1+ genes showed significantly higher expressed in astrocytes (*P* < 0.001), excitatory neurons (*P* < 0.001), inhibitory neurons (*P* < 0.001), and oligodendrocytes (*P* < 0.001), while showing significantly lower expression in microglia (*P* = 0.014; Figure 5d). At the laminar level, PLS1+ genes demonstrated preferentially higher expression in cortical layers III and IV (*P* < 0.001, for both; Figure 5e). Analysis using the CSEA web server revealed that PLS1+ genes exhibited widespread expression across cortical and subcortical brain regions, including the amygdala, striatum, and thalamus. Temporally, expression was predominantly observed during early fetal development and young adulthood (Figure 5f).

### Multiple linear regression analysis

**Figure 6.**
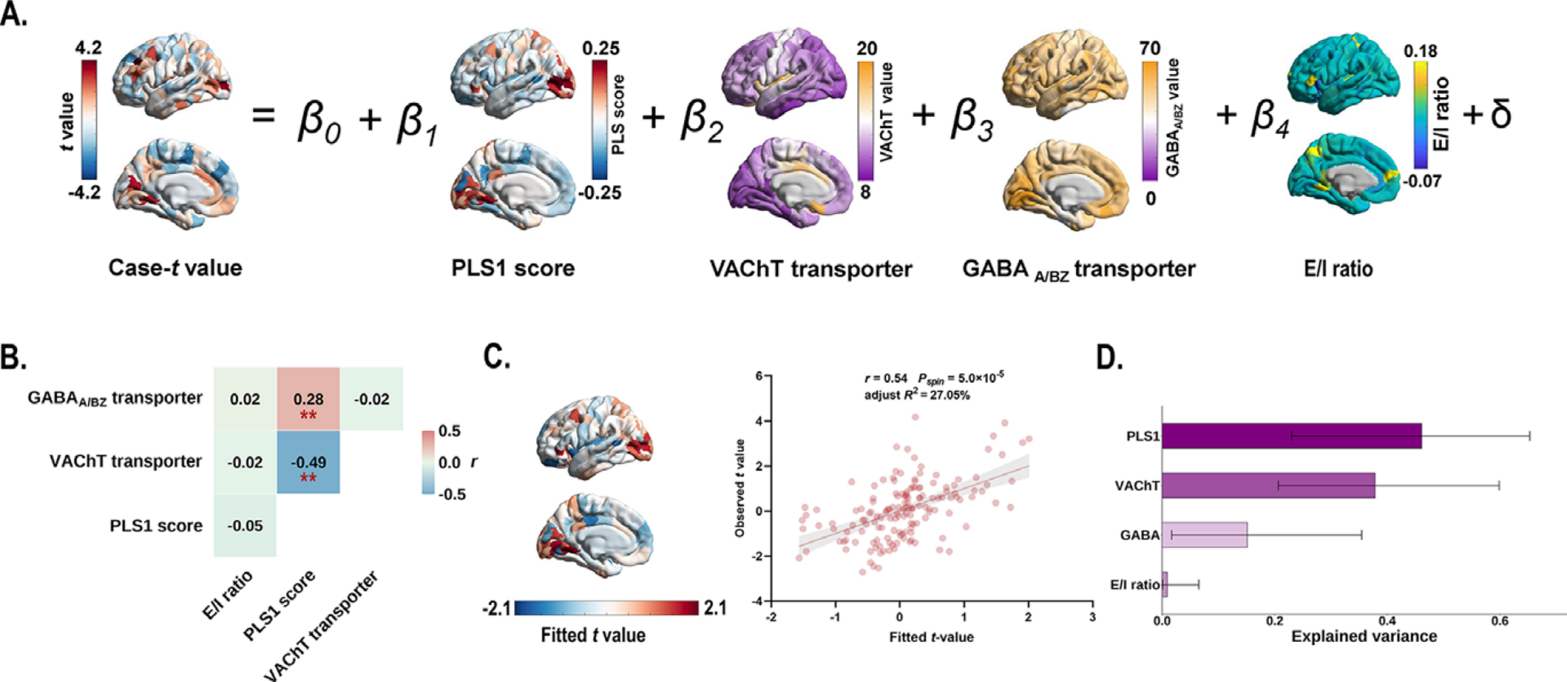
Multiple linear regression model showing the relationship between gene expression patterns, VAChT, GABA_A/BZ_ transporters, and case–control differences in the principal MIND gradient. (a) Schematic of the multiple linear regression model. (b) Heatmap showing Pearson’s correlation coefficients among gene expression patterns, VAChT, and GABA_A/BZ_ transporters. Asterisks (^**^) indicate *P_spin_* < 0.01. (c) The scatter plot displays the relationship between observed and fitted the principal MIND gradient alterations (*r* = 0.54, *P_spin_* = 5.0 × 10^−5^, adjusted *R*^2^ = 27.05%). The gray band indicates the 95% confidence interval. (d) Relative contribution (%) of each predictor in the multiple linear regression model. Error bars represent the 95% bootstrap confidence intervals. Abbreviations: GABA_A/BZ_, gamma-aminobutyric acid A/BZ; MIND, Morphometric Inverse Divergence; PLS, partial least squares; VAChT, vesicular acetylcholine transporter.

Multiple linear regression model that incorporated PLS1 scores, transporter densities (VAChT and GABA_A/BZ_), and *E*/*I* ratio as predictors of case–control gradient differences (Figure 6a). We found that the PLS1 scores significantly correlated with the VAChT transporter density (*r* = −0.49, *P_spin_* = 2.2 × 10^−3^; Figure 6b), and the GABA_A/BZ_ transporter density (*r* = 0.28, *P_spin_* = 7.3 × 10^−3^; Figure 6b). Explained 27.05% of the variance in MIND gradient alterations (adjusted *R*^2^ = 27.05%, *P_spin_* = 5.0 × 10^−5^; Figure 6c), with PLS1 score, VAChT, and GABA_A/BZ_ transporter serving as significant predictors. PLS1 score demonstrated the greatest contribution (46.10%), followed by VAChT (37.83%), GABA_A/BZ_ (15.13%), and excitatory/inhibitory ratio (0.93%) (Figure 6d).

### Validation analyses

Multiple validation analyses confirmed the robustness of our findings. (i) Case–control differences in the principal MIND gradient remained consistent when TIV as a covariate (*r* = 0.99, *P_spin_* < 1.0 × 10^−3^; Supplementary Figure S7). (ii) Primary results (top 10% threshold) correlated significantly with alternative thresholds of 20% (*r* = 0.49, *P_spin_* < 1.0 × 10^−3^; Supplementary Figure S8) and 30% (*r* = 0.39, *P_spin_* < 1.0 × 10^−3^; Supplementary Figure S8). (iii) Analysis of a randomly selected, size-matched healthy control subgroup yielded highly consistent results (*r* = 0.69, *P_spin_* < 1.0 × 10^−3^; Supplementary Figure S9), indicating that sample size imbalance did not drive our findings. (iv) External validation using three independent, cross-scanner, cross-race, and cross-age (CHCP, MICA-MICs, and SALD) datasets supported the reliability of the principal MIND gradient pattern (CHCP: *r* = 0.64, *P_spin_* < 1.0 × 10^−3^; MICA-MICs: *r* = 0.67, *P_spin_* < 1.0 × 10^−3^; SALD: *r* = 0.95, *P_spin_* < 1.0 × 10^−3^; Supplementary Figure S10–S12). (v) Gene-specific validation using *CACNA1C* and five *SST-*related genes revealed that two genes were included in the PLS1+ gene list and significantly correlated with the case–control *t*-map (*CACNA1C*: *r* = 0.34, *P_spin_* = 2.60 × 10^−3^; *NPY*: *r* = −0.31, *P_spin_* = 5.0 × 10^−3^; Supplementary Figure S13).

## Discussion

This study demonstrates that bipolar disorder (BD) is characterized by significant disruptions in cortical morphometric hierarchy, as captured by the principal MIND gradient. These alterations are spatially associated with specific neurotransmitter systems, cognitive processes, and gene expression patterns, providing converging evidence of BD pathophysiology that links disrupted macroscopic organization to its underlying molecular mechanisms.

The MIND network approach offers superior reliability compared to traditional morphometric similarity networks while capturing individual differences in cortical organization linked to development and genetic variation (Sebenius et al., 2023). Our findings reveal BD-specific alterations in the left lateral occipital and rostral middle frontal cortices, regions consistently implicated in structural neuroimaging studies of BD (Choi et al., 2022; Hanford et al., 2016; Hibar et al., 2018; Tu et al., 2024; Yongfeng Yang et al., 2022). The rostral middle frontal cortex, a core executive control network node (Friedman & Robbins, 2022; Ridderinkhof, Ullsperger, Crone, & Nieuwenhuis, 2004), showed disrupted hierarchical integration that may contribute to cognitive dysfunction in BD. Similarly, alterations in lateral occipital cortex suggest visual processing deficits, an emerging feature of the disorder (van den Boogert et al., 2022). Network-level analyses revealed widespread reorganization across functional systems, with decreased gradients in ventral attention and motor networks and increased gradients in frontoparietal and visual networks. This pattern indicates that BD involves distributed alterations in large-scale brain systems rather than localized dysfunction, consistent with the disorder’s complex symptomatology affecting mood regulation, executive function, and sensory processing (Gong et al., 2021).

The pathophysiology of BD arises from intricate biological interactions spanning from the genetic and molecular levels (e.g. neurotransmitter systems) to observable behavior (e.g. cognitive-behavioral processes) (Gillissie et al., 2022; Manji et al., 2003; O’Connell et al., 2025). Accordingly, BD-related alterations in the principal MIND gradient might also be multiscale changes. The integration of brain maps across multiple modalities offers a framework for examining the relationships among these different levels of biological and behavioral organization (Hansen et al., 2021; Hansen & Misic, 2025; Hansen, Shafiei, Markello, et al., 2022). The spatial correlation between MIND gradient alterations and acetylcholine (VAChT) and GABA (GABA_A/BZ_) transporter distributions suggests a potential mechanistic link to BD pathophysiology. Acetylcholine signaling influences mood and cognition through effects on myelinating glia (Picciotto, Higley, & Mineur, 2012), while GABAergic dysfunction disrupts inhibitory tone essential for emotional and cognitive regulation (Koh, Kwak, Cheong, & Lee, 2023). These findings align with emerging evidence implicating cholinergic and GABAergic dysfunction in BD vulnerability (Ji et al., 2023; Kaufman et al., 2009; Yohn, Breier, & Paul, 2024). Cognitive-behavioral mapping revealed that gradient alterations correlate with deficits in attention and executive function (Dickinson, Becerra, & Coombes, 2017; Sandstrom, Perroud, Alda, Uher, & Pavlova, 2021), core impairments in BD, while showing negative associations with auditory and language processing. This pattern links macroscale topological changes to specific cognitive domains, bridging neuroimaging findings with clinical phenotypes.

The significant spatial correlation between MIND gradient alterations and brain gene expression profiles demonstrates the transcriptional underpinnings of morphometric hierarchy disruptions. Notably, PLS1+ genes were enriched for BD risk variants from genome-wide association studies, including *CACNA1C* and *SST*-related genes, establishing a crucial connection between genetic vulnerability and structural alterations (Allen IV et al., 2024; Arbabi et al., 2025; Jiang, Sultan, et al., 2023; Owen et al., 2025; Pantazopoulos et al., 2017; Yang et al., 2024). This is particularly relevant given that the *CACNA1C* gene plays a key role in calcium signaling and neuronal excitability (Szymanowicz et al., 2024), while *SST* interneurons are central to regulating cortical circuit activity (Fee, Banasr, & Sibille, 2017; Lin & Sibille, 2015), both of which are strongly implicated in BD pathophysiology. Functional enrichment analyses further revealed that BD-associated genes primarily regulate gene expression and RNA metabolism, suggesting that morphometric alterations reflect fundamental disruptions in neuroplasticity mechanisms. These molecular processes are essential for the synthesis of new proteins and the remodeling of synapses, which are foundational to the brain’s ability to adapt and change (Kandel, Dudai, & Mayford, 2014; Sutton & Schuman, 2006). The preferential expression of these genes in astrocytes, excitatory neurons, inhibitory neurons, and oligodendrocytes across cortical layers III–IV provides critical cellular and structural context (Chana, Landau, Beasley, Everall, & Cotter, 2003). This points to cell-type-specific vulnerability patterns, impacting not only the excitatory-inhibitory balance but also glial support systems and long-range cortical communication. The identified critical windows for gene expression during early fetal development and young adulthood suggest a temporal specificity for BD susceptibility (Xue et al., 2023). This temporal pattern aligns with the neurodevelopmental hypothesis of BD, indicating that the disorder’s pathology may originate from molecular dysregulation during key periods of brain maturation.

Multiple regression analysis demonstrated that genetic architecture and neurotransmitter systems primarily drive morphometric alterations, while the classical excitatory/inhibitory ratio contributed minimally. These finding challenges traditional *E*/*I* balance hypotheses (Selten, van Bokhoven, & Kasri, 2018) and highlights the importance of specific neurochemical systems in BD pathophysiology.

Our study should be interpreted with respect to several limitations. First, the MIND gradient was constructed using only five morphometric features from anatomical MRI data; future research should incorporate multimodal imaging and microstructural metrics. Second, the relatively small sample size and broad age range limit generalizability; larger, age-stratified cohorts are needed to better characterize abnormalities in patients with BD. Third, transcriptomic data from healthy donors may not fully capture BD-specific expression patterns; patient-derived datasets would strengthen these findings. Fourth, the structural MRI field-of-view did not include the entire cerebellum in all participants, and the MIND method is currently optimized for cortical estimation. Consequently, we only calculated MIND gradients for the cerebral cortex, leaving cerebellar-cortical MIND gradient relationships unexplored. Given emerging evidence for cerebellar involvement in affective disorders, future investigations incorporating cerebellar-cortical gradient relationships would be valuable. Finally, our study primarily focused on genetic and molecular underpinnings and did not incorporate potential environmental exposure factors, such as air pollution or psychosocial stress, which have been shown to influence brain structure and are relevant to bipolar disorder pathophysiology (Liu et al., 2023; Zhang, Anderson, et al., 2025). Future research integrating both intrinsic biological factors and environmental exposures will be needed to provide a more comprehensive understanding of bipolar disorder etiology.

## Conclusion

Our findings highlight the utility of MIND gradients as robust imaging phenotypes that integrate cortical morphometry with molecular and genetic mechanisms. This multi-scale framework contextualizes BD-related cortical alterations within a continuum spanning genes, neurotransmitter systems, and cognition, providing novel insights into the neurobiological heterogeneity of the disorder.

## Supporting information

Wang et al. supplementary material 1 Wang et al. supplementary material

Wang et al. supplementary material 2 Wang et al. supplementary material

## Data Availability

Human gene expression data are available from the Allen Human Brain Atlas (https://human.brain-map.org/static/download). PET data are accessible at https://github.com/netneurolab/hansen_receptors, and Neurosynth maps are available at https://neurosynth.org/. Cognitive-behavioral terms were obtained from the Cognitive Atlas (https://cognitiveatlas.org/). The UCLA dataset is available at https://openneuro.org/datasets/ds000030/versions/1.0.0, the CHCP dataset at https://www.Chinese-HCP.cn, and the MICA-MICs dataset at https://osf.io/j532r/. BD-associated genes from AHBA can be found at (https://help.brainmap.org/download/attachments/2818165/HBA_ISH_GeneList.pdf?version=2&modificationDate=1614977648535&api=v2). Cell type data are available at https://github.com/jms290/PolySyn_MSNs/blob/master/Data/AHBA/celltypes_PSP.csv, and layer markers are from the raw He et al (Z. He et al., 2017) dataset (https://static-content.springer.com/esm/art%3A10.1038%2Fnn.4548/MediaObjects/41593_2017_BFnn4548_MOESM255_ESM.xlsx). High-resolution T1-weighted structural images were preprocessed using FreeSurfer (version: 6.0.1; http://surfer.nmr.mgh.harvard.edu/). Genetic data were preprocessed using the abagen toolbox (https://github.com/rmarkello/abagen). Gene enrichment analyses were conducted using Metascape (https://metascape.org/gp/index.html#/main/step1) and the CSEA tool (http://doughertytools.wustl.edu/CSEAtool.html). The MIND calculation code is available at https://github.com/isebenius/MIND, and MIND gradient construction code is available at https://github.com/MICA-MNI/BrainSpace. Codes for PLS analysis can be found at https://github.com/SarahMorgan/Morphometric_Similarity_SZ/blob/master/Gene_analyses.md. Spatial permutation testing code is available at https://github.com/frantisekvasa/rotate_parcellation. The brain surfaces were visualized using ENIGMA-toolbox (https://github.com/MICA-MNI/ENIGMA).

## Supplementary material

The supplementary material for this article can be found at http://doi.org/10.1017/S0033291725102705.
