# Supplementary material for: Cortical morphometric gradients reveal molecular and cognitive underpinnings of bipolar disorder
Source: Psychol Med. 2025 Dec 18;55:e383. doi: 10.1017/S0033291725102705 (PMC13058653; doi:10.1017/S0033291725102705)
Supplement: Wang et al. supplementary material 1 — Wang et al. supplementary material [file S0033291725102705sup001.docx]

**Supplementary materials**

**Title Page**

**Cortical** **Morphometric Inverse Divergence network gradient in** **bipolar disorder and its association with molecular underpinnings**

**Rui Wang ^1^, Jiajun Xu ^2^, Fei Li ^1^, Xiaoqi Huang ^1^, Chunchao Xia ^3^, Su Lui ^1^, Qiyong Gong ^1,^ ^4^, Huaiqiang Sun ^1, *^**

^1^ Department of Radiology, Institution of Radiology and Medical Imaging, West China Hospital of Sichuan University, Chengdu, 610041, Sichuan, China

^2^ Mental Health Center, West China Hospital, Sichuan University, Chengdu, China

^3^ Department of Radiology, West China Hospital, Sichuan University, Chengdu 610041, China

^4^ Xiamen Key Lab of Psychoradiology and Neuromodulation, Department of Radiology, West China Xiamen Hospital of Sichuan University, Xiamen, Fujian, China

***Correspondence to:**

Huaiqiang Sun, PhD, MD E-mail: sunhuaiqiang@scu.edu.cn

Department of Radiology, Institution of Radiology and Medical Imaging, West China Hospital, Sichuan University, Chengdu, 610041, China.

**Supplementary materials**

**Contents**

[**Supplementary Methods** 4](#_Toc213581342)

[**Supplementary method 1** Quality control of high-resolution structural MRI data 4](#_Toc213581343)

[**Supplementary method 2** MIND network construction 4](#_Toc213581344)

[**Supplementary method 3** BD-related GWAS gene set analyses 5](#_Toc213581345)

[**Supplementary method 4** Enrichment analyses 6](#_Toc213581346)

[**Supplementary method 5** Null models 7](#_Toc213581347)

[**Supplementary Methods References** 9](#_Toc213581348)

[**Supplementary Tables** 11](#_Toc213581349)

[**Table S1.** Demographic of the validation datasets. 11](#_Toc213581350)

[**Table S2.** High-resolution structural MRI data acquisition parameters for four datasets. 12](#_Toc213581351)

[**Table S3.** Desikan–Killiany (DK308) atlas parcellation with corresponding Yeo functional network labels. 13](#_Toc213581352)

[**Table S4.** Desikan–Killiany (DK308) atlas parcellation with corresponding von Economo class labels. 23](#_Toc213581353)

[**Table S5.** Regional MIND gradient differences between BD and healthy controls. 33](#_Toc213581354)

[**Table S6.** The differences in the principal MIND gradient in each Yeo functional network. 34](#_Toc213581355)

[**Table S7.** The differences in the principal MIND gradient in each von Economo class. 35](#_Toc213581356)

[**Table S8.**  Neurotransmitter receptors and transporters included in this study. 36](#_Toc213581357)

[**Table S9.** Association of neurotransmitter systems with the principal MIND gradient alterations. 38](#_Toc213581358)

[**Table S10.** Cognitive-behavioral terms 40](#_Toc213581359)

[**Table S11.** The association of cognitive-behavioral processes with the principal MIND gradient alterations. 42](#_Toc213581360)

[**Table S12.** The six donors’ information in the AHBA database. 49](#_Toc213581361)

[**Table S13.** BD-related genes from the AHBA database. 50](#_Toc213581362)

[**Supplementary Figures** 51](#_Toc213581363)

[**Figure S1.** Variance of the MIND network explained by the gradient components. 51](#_Toc213581364)

[**Figure S2.** The spatial pattern of the 1-5 MIND gradient components. 52](#_Toc213581365)

[**Figure S3.** The spatial maps of Yeo functional networks and von Economo classes. 53](#_Toc213581366)

[**Figure S4.** Distribution of the principal MIND gradient in BD and HC. 54](#_Toc213581367)

[**Figure S5.** Distribution of PLS1 Weighted *Z-*score. 55](#_Toc213581368)

[**Figure S6.** The significant correlations between the two BD-related genes from the AHBA database and the case-control *t*-map. 56](#_Toc213581369)

[**Figure S7.** TIV effect on case-control differences. 57](#_Toc213581370)

[**Figure S8.** The effect of different thresholds (top 20% and 30%) on the principal MIND gradient’s manifestation in case-control differences. 58](#_Toc213581371)

[**Figure S9.** Sample matching effect on case-control differences. 60](#_Toc213581372)

[**Figure S10.** The replicable principal gradient of MIND in healthy controls. 61](#_Toc213581373)

[**Figure S11.** The replicable principal gradient of MIND in healthy controls. 62](#_Toc213581374)

[**Figure S12.** The replicable principal gradient of MIND in healthy controls. 63](#_Toc213581375)

[**Figure S13.** Significant correlations of *CACNA1C* and *SST*-related gene expression with the case-control *t*-map. 64](#_Toc213581376)

[**Supplementary Tables and Figures References** 65](#_Toc213581377)

**Supplementary Methods**

**Supplementary method 1** Quality control of high-resolution structural MRI data

A rigorous quality control (QC) procedure was implemented for all high-resolution structural MRI data. This process began with a visual inspection of the raw data prior to preprocessing to exclude scans with poor image quality, and motion artifacts. Subsequently, the preprocessed data underwent a second thorough inspection to identify and correct various processing errors, including faulty tissue segmentation, inaccurate non-brain tissue removal, errors in intensity normalization, misplacement of pial surfaces, and topological defects. For any participant whose data failed QC, we attempted to correct the issue and rerun the preprocessing pipeline. Following this comprehensive QC process, the final sample comprised 49 individuals with BD and 119 healthy controls from CNP, 353 healthy individuals from CHCP, 50 healthy individuals from MICA-MICs, and 95 healthy individuals from SALD.

**Supplementary method 2** MIND network construction

Morphometric INverse Divergence (MIND) is a new method for estimating morphometric similarity based on structural MRI data (Sebenius et al., 2023). Specifically, each cortical area is represented by a multidimensional distribution of several structural MRI features measured across vertices—for instance, vertex-wise measures of cortical thickness (CT) and curvature. The MIND similarity between pairs of regions is computed using the symmetric Kullback–Leibler (KL) divergence, also known as Jeffrey’s divergence. This metric quantifies the difference between the multivariate distributions of morphometric features for two regions. For a given pair of regions ***x*** and ***y****,* let ***P_x_*** and ***P_y_*** represent the true multivariate distribution of morphometric features for each region. The KL divergence of region ***x*** from region ***y*** is denoted as ***D_xy_ (P_x_ || P_y_)****.* To convert this to symmetric version, the method combines ***D_KL_ (P_x_ || P_y_)*** with ***D_KL_ (P_y_ || P_x_)*** to define the overall divergence ***D (P_x_, P_y_)***, which represents the morphometric dissimilarity between the two regions. Finally, this KL divergence is transformed into a similarity measure, , using the below formula that bounds the value between 0 and 1, where higher values indicate greater morphometric similarity between the regions (Sebenius et al., 2023).


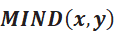

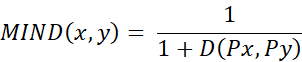


In this study, each vertex of individual surface was characterized by five morphometric features including gray matter volume (GMV), cortical thickness (CT), surface area (SA), mean curvature (MC), and sulcal depth (SD). Based on abovementioned features, each of them was standardized (z-score) across all vertices in the brain, and then the data was divided into predefined 308 parcellations to create a regional multivariate distribution. Subsequently we calculated MIND similarity statistic using the transformed Kullback-Leibler (KL) divergence for any two cortical regions to assess inter-regional MIND similarity, bounded between 0 and 1. Finally, we generated the MIND network, a 308 × 308 similarity matrix for each participant.

**Supplementary method 3** BD-related GWAS gene set analyses

We conducted gene set analyses on the PLS1+ and PLS1− genes using *MAGMA* v1.10 (de Leeuw, Mooij, Heskes, & Posthuma, 2015) to assess enrichment for BD risk genes from the GWAS. Summary statistics from a large-scale BD-related GWAS results were utilized, with gene-wide *P* values obtained by aggregating *P* values of all SNPs within genes. A window of 35 kb upstream and 10 kb downstream of each gene was used to capture SNPs in approximate regulatory regions (Morgan et al., 2019). One-tailed competitive gene set analyses were performed for both PLS1+ and PLS1− genes, revealing significant enrichment only for PLS1+ genes in BD risk genes identified by the GWAS (*P*_PLS1_+ = 0.0322 and *P*_PLS1−_ = 0.1482).

**Supplementary method 4** Enrichment analyses

We performed a series of enrichment analyses to further investigate the genes identified in our study. First, Gene Ontology (GO) and human diseases from DisGeNET database embedded in *Metascape* (<https://metascape.org/gp/index.html#/main/step1>) (Zhou et al., 2019)were used for functional annotations of the PLS1+ genes. The 15,632 genes with qualified brain expression data were used as background. Second, to explore the cellular specificity linked with regional alterations in the principal MIND gradient, we conducted cell type enrichment analyses. Specifically, we computed the ratio of genes within significant PLS1+ gene sets that displayed preferential expression in one of seven major brain cell classes: astrocytes, endothelial cells, microglia, excitatory neurons, inhibitory neurons, oligodendrocytes, and oligodendrocyte precursors (OPCs). These cell markers were identified from five human adult postmortem single-cell and single-nucleus RNA sequencing studies (Darmanis et al., 2015; Habib et al., 2017; Lake et al., 2018; Li et al., 2018; McKenzie et al., 2018; Zhang et al., 2016). Specifically, we first computed the ratio of genes in significant PLS1+ gene set that exhibited preferential expression in one of the seven major brain cell classes. Statistical significance was evaluated by comparing the observed ratios to a null distribution generated by repeating the process 10,000 times on a set of randomly selected genes (Hansen et al., 2021). Third, cortical layer enrichment analysis was applied by using marker genes obtained based on a previous transcriptomic study (He et al., 2017), which included 772 genes for layer I, 483 genes for layer II, 294 genes for layer III, 308 genes for layer IV, 115 genes for layer V, and 2,159 genes for layer VI. Cortical layer enrichment was then performed by using cortical layer marker genes following the abovementioned method as cell type enrichment. Finally, cell-type specific expression analysis (CSEA) tool (<http://doughertytools.wustl.edu/CSEAtool.html>) (Dougherty, Schmidt, Nakajima, & Heintz, 2010) was employed to perform developmental gene expression enrichment analysis to investigate developmental time windows across brain regions. All enrichment analyses used a significance threshold of *P* < 0.05 after BH-FDR correction.

**Supplementary method 5** Null models

To mitigate potential confounding effects of spatial autocorrelations, we conducted spin tests in our study (Alexander-Bloch et al., 2018). This test generated a set of null Pearson’s correlation coefficients by randomly rotating the spherical projection of spatial maps while preserving their spatial relationships. Initially, we conducted 10,000 spin test permutations of cortical regions to create a null distribution. The *P_spin_* value was calculated by determining the proportion of null values of the correlation coefficient that exceed the real values of the correlation coefficient.

**Supplementary Methods References**

Alexander-Bloch, A. F., Shou, H., Liu, S., Satterthwaite, T. D., Glahn, D. C., Shinohara, R. T., . . . Raznahan, A. (2018). On testing for spatial correspondence between maps of human brain structure and function. *Neuroimage, 178*, 540-551.

Darmanis, S., Sloan, S. A., Zhang, Y., Enge, M., Caneda, C., Shuer, L. M., . . . Quake, S. R. (2015). A survey of human brain transcriptome diversity at the single cell level. *Proc Natl Acad Sci U S A, 112*(23), 7285-7290. doi:10.1073/pnas.1507125112

de Leeuw, C. A., Mooij, J. M., Heskes, T., & Posthuma, D. (2015). MAGMA: generalized gene-set analysis of GWAS data. *PLoS Comput Biol, 11*(4), e1004219. doi:10.1371/journal.pcbi.1004219

Dougherty, J. D., Schmidt, E. F., Nakajima, M., & Heintz, N. (2010). Analytical approaches to RNA profiling data for the identification of genes enriched in specific cells. *Nucleic Acids Res, 38*(13), 4218-4230. doi:10.1093/nar/gkq130

Habib, N., Avraham-Davidi, I., Basu, A., Burks, T., Shekhar, K., Hofree, M., . . . Regev, A. (2017). Massively parallel single-nucleus RNA-seq with DroNc-seq. *Nat Methods, 14*(10), 955-958. doi:10.1038/nmeth.4407

Hansen, J. Y., Markello, R. D., Vogel, J. W., Seidlitz, J., Bzdok, D., & Misic, B. (2021). Mapping gene transcription and neurocognition across human neocortex. *Nat Hum Behav, 5*(9), 1240-1250. doi:10.1038/s41562-021-01082-z

He, Z., Han, D., Efimova, O., Guijarro, P., Yu, Q., Oleksiak, A., . . . Khaitovich, P. (2017). Comprehensive transcriptome analysis of neocortical layers in humans, chimpanzees and macaques. *Nat Neurosci, 20*(6), 886-895. doi:10.1038/nn.4548

Lake, B. B., Chen, S., Sos, B. C., Fan, J., Kaeser, G. E., Yung, Y. C., . . . Zhang, K. (2018). Integrative single-cell analysis of transcriptional and epigenetic states in the human adult brain. *Nat Biotechnol, 36*(1), 70-80. doi:10.1038/nbt.4038

Li, M., Santpere, G., Imamura Kawasawa, Y., Evgrafov, O. V., Gulden, F. O., Pochareddy, S., . . . Sestan, N. (2018). Integrative functional genomic analysis of human brain development and neuropsychiatric risks. *Science, 362*(6420). doi:10.1126/science.aat7615

McKenzie, A. T., Wang, M., Hauberg, M. E., Fullard, J. F., Kozlenkov, A., Keenan, A., . . . Zhang, B. (2018). Brain Cell Type Specific Gene Expression and Co-expression Network Architectures. *Sci Rep, 8*(1), 8868. doi:10.1038/s41598-018-27293-5

Morgan, S. E., Seidlitz, J., Whitaker, K. J., Romero-Garcia, R., Clifton, N. E., Scarpazza, C., . . . Bullmore, E. T. (2019). Cortical patterning of abnormal morphometric similarity in psychosis is associated with brain expression of schizophrenia-related genes. *Proc Natl Acad Sci U S A, 116*(19), 9604-9609. doi:10.1073/pnas.1820754116

Sebenius, I., Seidlitz, J., Warrier, V., Bethlehem, R. A. I., Alexander-Bloch, A., Mallard, T. T., . . . Morgan, S. E. (2023). Robust estimation of cortical similarity networks from brain MRI. *Nature Neuroscience, 26*(8), 1461-1471. doi:10.1038/s41593-023-01376-7

Zhang, Y., Sloan, S. A., Clarke, L. E., Caneda, C., Plaza, C. A., Blumenthal, P. D., . . . Barres, B. A. (2016). Purification and Characterization of Progenitor and Mature Human Astrocytes Reveals Transcriptional and Functional Differences with Mouse. *Neuron, 89*(1), 37-53. doi:10.1016/j.neuron.2015.11.013

Zhou, Y., Zhou, B., Pache, L., Chang, M., Khodabakhshi, A. H., Tanaseichuk, O., . . . Chanda, S. K. (2019). Metascape provides a biologist-oriented resource for the analysis of systems-level datasets. *Nat Commun, 10*(1), 1523. doi:10.1038/s41467-019-09234-6

**Supplementary Tables**

**Table S1.** Demographic of the validation datasets.

| **Characteristics** | **CHCP** | **MICA-MICs** | **SALD** |
| --- | --- | --- | --- |
| Sample size | 353 | 50 | 95 |
| Age (years) | 33.98 ± 18.12 | 29.54 ± 5.62 | 67.95 ± 5.85 |
| Gender (F/M) | 186/167 | 23/27 | 55/40 |

Age is expressed as mean ± standard deviation.

Abbreviations: CHCP, Chinese Human Connectome Project; MICA-MICs, Multimodal Imaging and Connectome Analysis-Microstructure-Informed Connectomics; SALD, Southwest University Adult Lifespan Dataset.

**Table S2.** High-resolution structural MRI data acquisition parameters for four datasets.

| **Parameters** | **CNP** | **CHCP** | **MICA-MICs** | **SALD** |
| --- | --- | --- | --- | --- |
| Scanner | 3.0T Siemens Trio | 3.0 T Siemens Prisma | 3.0 T Siemens Prisma | 3.0T Siemens Trio |
| Sequence | MPRAGE | MPRAGE | MPRAGE | MPRAGE |
| TR (ms) | 1900 | 2400 | 2300 | 1900 |
| TE (ms) | 2.26 | 2.22 | 3.14 | 2.52 |
| FOV (mm^2^) | 250 × 250 | 256 × 240 | 256 × 256 | 256 × 256 |
| Matrix size | 256 × 256 | 320 × 300 | 320 × 320 | 256 × 256 |
| Slice thickness (mm) | 1.0 | 0.8 | 0.8 | 1.0 |
| Slice gap (mm) | - | - | - | - |
| Slices | 176 | 224 | 224 | 176 |

Abbreviations: CHCP, Chinese Human Connectome Project; CNP, Consortium for Neuropsychiatric Phenomics; FA, flip angle; FOV, field of view; MICA-MICs, Multimodal Imaging and Connectome Analysis-Microstructure-Informed Connectomics; MPRAGE, Magnetization-Prepared Rapid Gradient-Echo; SALD, Southwest University Adult Lifespan Dataset; TR, repetition time; TE, echo time.

**Table S3.** Desikan–Killiany (DK308) atlas parcellation with corresponding Yeo functional network labels.

| **Yeo functional network** | **Label_ID** | **Hemisphere** | **Cortical region** | **Label_ID** | **Hemisphere** | **Cortical region** |
| --- | --- | --- | --- | --- | --- | --- |
| **DAN** | 5 | Left hemisphere | lh_caudalmiddlefrontal_part2 | 169 | Right hemisphere | rh_inferiorparietal_part1 |
|  | 19 |  | lh_inferiorparietal_part4 | 172 |  | rh_inferiorparietal_part4 |
|  | 20 |  | lh_inferiorparietal_part5 | 175 |  | rh_inferiorparietal_part7 |
|  | 25 |  | lh_inferiortemporal_part2 | 180 |  | rh_inferiortemporal_part2 |
|  | 55 |  | lh_middletemporal_part2 | 182 |  | rh_inferiortemporal_part4 |
|  | 89 |  | lh_precentral_part8 | 211 |  | rh_middletemporal_part4 |
|  | 112 |  | lh_superiorfrontal_part4 | 241 |  | rh_precentral_part3 |
|  | 125 |  | lh_superiorparietal_part4 | 247 |  | rh_precentral_part9 |
|  | 126 |  | lh_superiorparietal_part5 | 250 |  | rh_precuneus_part3 |
|  | 128 |  | lh_superiorparietal_part7 | 252 |  | rh_precuneus_part5 |
|  | 129 |  | lh_superiorparietal_part8 | 270 |  | rh_superiorfrontal_part5 |
|  | 130 |  | lh_superiorparietal_part9 | 280 |  | rh_superiorparietal_part2 |
|  | 131 |  | lh_superiorparietal_part10 | 283 |  | rh_superiorparietal_part5 |
|  | 144 |  | lh_supramarginal_part6 | 284 |  | rh_superiorparietal_part6 |
|  | - |  | - | 285 |  | rh_superiorparietal_part7 |
|  | - |  | - | 286 |  | rh_superiorparietal_part8 |
|  | - |  | - | 287 |  | rh_superiorparietal_part9 |
|  | - |  | - | 288 |  | rh_superiorparietal_part10 |
|  | - |  | - | 295 |  | rh_superiorparietal_part11 |

| **Yeo functional network** | **Label_ID** | **Hemisphere** | **Cortical region** | **Label_ID** | **Hemisphere** | **Cortical region** | |
| --- | --- | --- | --- | --- | --- | --- | --- |
| **DMN** | 1 | Left hemisphere | lh_bankssts_part1 | 153 | Right hemisphere | rh_bankssts_part1 | |
|  | 2 |  | lh_bankssts_part2 | 171 |  | rh_inferiorparietal_part3 | |
|  | 4 |  | lh_caudalmiddlefrontal_part1 | 176 |  | rh_inferiorparietal_part8 | |
|  | 7 |  | lh_caudalmiddlefrontal_part4 | 178 |  | rh_inferiorparietal_part10 | |
|  | 16 |  | lh_inferiorparietal_part1 | 185 |  | rh_isthmuscingulate_part2 | |
|  | 18 |  | lh_inferiorparietal_part3 | 197 |  | rh_lateralorbitofrontal_part3 | |
|  | 21 |  | lh_inferiorparietal_part6 | 205 |  | rh_medialorbitofrontal_part1 | |
|  | 22 |  | lh_inferiorparietal_part7 | 209 |  | rh_middletemporal_part2 | |
|  | 23 |  | lh_inferiorparietal_part8 | 210 |  | rh_middletemporal_part3 | |
|  | 30 |  | lh_isthmuscingulate_part1 | 213 |  | rh_middletemporal_part6 | |
|  | 31 |  | lh_isthmuscingulate_part2 | 222 |  | rh_parsorbitalis_part1 | |
|  | 44 |  | lh_lateralorbitofrontal_part4 | 223 |  | rh_parstriangularis_part1 | |
|  | 52 |  | lh_medialorbitofrontal_part2 | 249 |  | rh_precuneus_part2 | |
|  | 54 |  | lh_middletemporal_part1 | 253 |  | rh_precuneus_part6 | |
|  | 56 |  | lh_middletemporal_part3 | 254 |  | rh_precuneus_part7 | |
|  | 57 |  | lh_middletemporal_part4 | 255 |  | rh_rostralanteriorcingulate_part1 | |
|  | 58 |  | lh_middletemporal_part5 | 267 |  | rh_superiorfrontal_part2 | |
|  | 64 |  | lh_parsopercularis_part1 | 269 |  | rh_superiorfrontal_part4 | |
|  | 67 |  | lh_parsorbitalis_part1 | 271 |  | rh_superiorfrontal_part6 | |
|  | 68 |  | lh_parstriangularis_part1 | 273 |  | rh_superiorfrontal_part8 | |
| **Yeo functional network** | **Label_ID** | **Hemisphere** | **Cortical region** | **Label_ID** | **Hemisphere** | | **Cortical region** |
| **DMN** | 69 | Left hemisphere | lh_parstriangularis_part2 | 275 | Right hemisphere | | rh_superiorfrontal_part10 |
|  | 91 |  | lh_precuneus_part1 | 276 |  | | rh_superiorfrontal_part11 |
|  | 93 |  | lh_precuneus_part3 | 277 |  | | rh_superiorfrontal_part12 |
|  | 94 |  | lh_precuneus_part4 | 289 |  | | rh_superiortemporal_part1 |
|  | 95 |  | lh_precuneus_part5 | 291 |  | | rh_superiortemporal_part3 |
|  | 96 |  | lh_precuneus_part6 | 292 |  | | rh_superiortemporal_part4 |
|  | 97 |  | lh_precuneus_part7 | 294 |  | | rh_superiortemporal_part6 |
|  | 98 |  | lh_rostralanteriorcingulate_part1 | - |  | | - |
|  | 106 |  | lh_rostralmiddlefrontal_part8 | - |  | | - |
|  | 109 |  | lh_superiorfrontal_part1 | - |  | | - |
|  | 111 |  | lh_superiorfrontal_part3 | - |  | | - |
|  | 113 |  | lh_superiorfrontal_part5 | - |  | | - |
|  | 115 |  | lh_superiorfrontal_part7 | - |  | | - |
|  | 116 |  | lh_superiorfrontal_part8 | - |  | | - |
|  | 117 |  | lh_superiorfrontal_part9 | - |  | | - |
|  | 119 |  | lh_superiorfrontal_part11 | - |  | | - |
|  | 120 |  | lh_superiorfrontal_part12 | - |  | | - |
|  | 133 |  | lh_superiortemporal_part2 | - |  | | - |
|  | 135 |  | lh_superiortemporal_part4 | - |  | | - |
|  | 136 |  | lh_superiortemporal_part5 | - |  | | - |

| **Yeo functional network** | **Label_ID** | **Hemisphere** | **Cortical region** | **Label_ID** | **Hemisphere** | **Cortical region** |
| --- | --- | --- | --- | --- | --- | --- |
| **DMN** | 138 | Left hemisphere | lh_superiortemporal_part7 | - | Right hemisphere | - |
| **FPN** | 6 |  | lh_caudalmiddlefrontal_part3 | 156 |  | rh_caudalmiddlefrontal_part1 |
|  | 27 |  | lh_inferiortemporal_part4 | 157 |  | rh_caudalmiddlefrontal_part2 |
|  | 29 |  | lh_inferiortemporal_part6 | 158 |  | rh_caudalmiddlefrontal_part3 |
|  | 66 |  | lh_parsopercularis_part3 | 159 |  | rh_caudalmiddlefrontal_part4 |
|  | 99 |  | lh_rostralmiddlefrontal_part1 | 174 |  | rh_inferiorparietal_part6 |
|  | 100 |  | lh_rostralmiddlefrontal_part2 | 177 |  | rh_inferiorparietal_part9 |
|  | 103 |  | lh_rostralmiddlefrontal_part5 | 208 |  | rh_middletemporal_part1 |
|  | 104 |  | lh_rostralmiddlefrontal_part6 | 212 |  | rh_middletemporal_part5 |
|  | 107 |  | lh_rostralmiddlefrontal_part9 | 220 |  | rh_parsopercularis_part2 |
|  | 108 |  | lh_rostralmiddlefrontal_part10 | 221 |  | rh_parsopercularis_part3 |
|  | 121 |  | lh_superiorfrontal_part13 | 225 |  | rh_parstriangularis_part3 |
|  | 139 |  | lh_supramarginal_part1 | 238 |  | rh_posteriorcingulate_part2 |
|  | 141 |  | lh_supramarginal_part3 | 256 |  | rh_rostralmiddlefrontal_part1 |
|  | - |  | - | 257 |  | rh_rostralmiddlefrontal_part2 |
|  | - |  | - | 258 |  | rh_rostralmiddlefrontal_part3 |
|  | - |  | - | 259 |  | rh_rostralmiddlefrontal_part4 |
|  | - |  | - | 260 |  | rh_rostralmiddlefrontal_part5 |
|  | - |  | - | 261 |  | rh_rostralmiddlefrontal_part6 |
|  | - |  | - | 262 |  | rh_rostralmiddlefrontal_part7 |

| **Yeo functional network** | **Label_ID** | **Hemisphere** | **Cortical region** | **Label_ID** | | **Hemisphere** | | **Cortical region** | |  |
| --- | --- | --- | --- | --- | --- | --- | --- | --- | --- | --- |
| **FPN** | - | Left hemisphere | - | 264 | | Right hemisphere | | rh_rostralmiddlefrontal_part9 | |  |
|  | - |  | - | 274 | |  | | rh_superiorfrontal_part9 | |  |
|  | - |  | - | 278 | |  | | rh_superiorfrontal_part13 | |  |
|  | - |  | - | 297 | |  | | rh_supramarginal_part3 | |  |
| **LN** | 10 |  | lh_entorhinal_part1 | 163 | |  | | rh_entorhinal_part1 | |  |
|  | 12 |  | lh_fusiform_part2 | 165 | |  | | rh_fusiform_part2 | |  |
|  | 14 |  | lh_fusiform_part4 | 179 | |  | | rh_inferiortemporal_part1 | |  |
|  | 24 |  | lh_inferiortemporal_part1 | 181 | |  | | rh_inferiortemporal_part3 | |  |
|  | 26 |  | lh_inferiortemporal_part3 | 183 | |  | | rh_inferiortemporal_part5 | |  |
|  | 28 |  | lh_inferiortemporal_part5 | 195 | |  | | rh_lateralorbitofrontal_part1 | |  |
|  | 41 |  | lh_lateralorbitofrontal_part1 | 196 | |  | | rh_lateralorbitofrontal_part2 | |  |
|  | 42 |  | lh_lateralorbitofrontal_part2 | 198 | |  | | rh_lateralorbitofrontal_part4 | |  |
|  | 43 |  | lh_lateralorbitofrontal_part3 | 206 | |  | | rh_medialorbitofrontal_part2 | |  |
|  | 51 |  | lh_medialorbitofrontal_part1 | 207 | |  | | rh_medialorbitofrontal_part3 | |  |
|  | 53 |  | lh_medialorbitofrontal_part3 | 215 | |  | | rh_parahippocampal_part2 | |  |
|  | 59 |  | lh_parahippocampal_part1 | 302 | |  | | rh_frontalpole_part1 | |  |
|  | 102 |  | lh_rostralmiddlefrontal_part4 | 303 | |  | | rh_temporalpole_part1 | |  |
|  | 146 |  | lh_frontalpole_part1 | - | |  | | - | |  |
|  | 147 |  | lh_temporalpole_part1 | - | |  | | - | |  |
|  | - |  | - | - | |  | | - | |  |
| **Yeo functional network** | **Label_ID** | **Hemisphere** | **Cortical region** | | **Label_ID** | | **Hemisphere** | | **Cortical region** | |
| **SMN** | 61 | Left hemisphere | lh_paracentral_part1 | | 217 | | Right hemisphere | | rh_paracentral_part2 | |
|  | 62 |  | lh_paracentral_part2 | | 218 | |  | | rh_paracentral_part3 | |
|  | 63 |  | lh_paracentral_part3 | | 229 | |  | | rh_postcentral_part1 | |
|  | 72 |  | lh_postcentral_part1 | | 230 | |  | | rh_postcentral_part2 | |
|  | 73 |  | lh_postcentral_part2 | | 231 | |  | | rh_postcentral_part3 | |
|  | 74 |  | lh_postcentral_part3 | | 232 | |  | | rh_postcentral_part4 | |
|  | 75 |  | lh_postcentral_part4 | | 233 | |  | | rh_postcentral_part5 | |
|  | 76 |  | lh_postcentral_part5 | | 234 | |  | | rh_postcentral_part6 | |
|  | 77 |  | lh_postcentral_part6 | | 235 | |  | | rh_postcentral_part7 | |
|  | 78 |  | lh_postcentral_part7 | | 236 | |  | | rh_postcentral_part8 | |
|  | 79 |  | lh_postcentral_part9 | | 240 | |  | | rh_precentral_part2 | |
|  | 82 |  | lh_precentral_part1 | | 242 | |  | | rh_precentral_part4 | |
|  | 84 |  | lh_precentral_part3 | | 243 | |  | | rh_precentral_part5 | |
|  | 86 |  | lh_precentral_part5 | | 244 | |  | | rh_precentral_part6 | |
|  | 87 |  | lh_precentral_part6 | | 245 | |  | | rh_precentral_part7 | |
|  | 88 |  | lh_precentral_part7 | | 246 | |  | | rh_precentral_part8 | |
|  | 90 |  | lh_precentral_part9 | | 290 | |  | | rh_superiortemporal_part2 | |
|  | 110 |  | lh_superiorfrontal_part2 | | 293 | |  | | rh_superiortemporal_part5 | |
|  | 114 |  | lh_superiorfrontal_part6 | | 296 | |  | | rh_supramarginal_part2 | |
|  | 122 |  | lh_superiorparietal_part1 | | 304 | |  | | rh_transversetemporal_part1 | |

| **Yeo functional network** | **Label_ID** | **Hemisphere** | **Cortical region** | **Label_ID** | **Hemisphere** | **Cortical region** |  |
| --- | --- | --- | --- | --- | --- | --- | --- |
| **SMN** | 124 | Left hemisphere | lh_superiorparietal_part3 | 306 | Right hemisphere | rh_insula_part2 |  |
|  | 132 |  | lh_superiortemporal_part1 | - |  | - |  |
|  | 134 |  | lh_superiortemporal_part3 | - |  | - |  |
|  | 137 |  | lh_superiortemporal_part6 | - |  | - |  |
|  | 140 |  | lh_supramarginal_part2 | - |  | - |  |
|  | 148 |  | lh_transversetemporal_part1 | - |  | - |  |
|  | 149 |  | lh_insula_part1 | - |  | - |  |
| **VAN** | 3 |  | lh_caudalanteriorcingulate_part1 | 154 |  | rh_bankssts_part2 |  |
|  | 65 |  | lh_parsopercularis_part2 | 155 |  | rh_caudalanteriorcingulate_part1 |  |
|  | 80 |  | lh_posteriorcingulate_part1 | 216 |  | rh_paracentral_part1 |  |
|  | 81 |  | lh_posteriorcingulate_part2 | 219 |  | rh_parsopercularis_part1 |  |
|  | 83 |  | lh_precentral_part2 | 224 |  | rh_parsopercularis_part2 |  |
|  | 85 |  | lh_precentral_part4 | 237 |  | rh_posteriorcingulate_part1 |  |
|  | 92 |  | lh_precuneus_part2 | 239 |  | rh_precentral_part1 |  |
|  | 101 |  | lh_rostralmiddlefrontal_part3 | 263 |  | rh_rostralmiddlefrontal_part8 |  |
|  | 105 |  | lh_rostralmiddlefrontal_part7 | 265 |  | rh_rostralmiddlefrontal_part10 |  |
|  | 118 |  | lh_superiorfrontal_part10 | 266 |  | rh_superiorfrontal_part1 |  |
|  | 142 |  | lh_supramarginal_part4 | 268 |  | rh_superiorfrontal_part3 |  |
|  | 143 |  | lh_supramarginal_part5 | 272 |  | rh_superiorfrontal_part7 |  |
|  | 145 |  | lh_supramarginal_part7 | 298 |  | rh_supramarginal_part4 |  |
| **Yeo functional network** | **Label_ID** | **Hemisphere** | **Cortical region** | **Label_ID** | **Hemisphere** | **Cortical region** | |
| **VAN** | 150 | Left hemisphere | lh_insula_part2 | 299 | Right hemisphere | rh_supramarginal_part5 | |
|  | 151 |  | lh_insula_part3 | 300 |  | rh_supramarginal_part6 | |
|  | 152 |  | lh_insula_part4 | 301 |  | rh_supramarginal_part7 | |
|  | - |  | - | 305 |  | rh_insula_part1 | |
|  | - |  | - | 307 |  | rh_insula_part3 | |
|  | - |  | - | 308 |  | rh_insula_part4 | |
| **VIS** | 8 |  | lh_cuneus_part1 | 160 |  | rh_cuneus_part1 | |
|  | 9 |  | lh_cuneus_part2 | 161 |  | rh_cuneus_part2 | |
|  | 11 |  | lh_fusiform_part1 | 162 |  | rh_cuneus_part3 | |
|  | 13 |  | lh_fusiform_part3 | 164 |  | rh_fusiform_part1 | |
|  | 15 |  | lh_fusiform_part5 | 166 |  | rh_fusiform_part3 | |
|  | 17 |  | lh_inferiorparietal_part2 | 167 |  | rh_fusiform_part4 | |
|  | 32 |  | lh_lateraloccipital_part1 | 168 |  | rh_fusiform_part5 | |
|  | 33 |  | lh_lateraloccipital_part2 | 170 |  | rh_inferiorparietal_part2 | |
|  | 34 |  | lh_lateraloccipital_part3 | 173 |  | rh_inferiorparietal_part5 | |
|  | 35 |  | lh_lateraloccipital_part4 | 184 |  | rh_isthmuscingulate_part1 | |
|  | 36 |  | lh_lateraloccipital_part5 | 186 |  | rh_lateraloccipital_part1 | |
|  | 37 |  | lh_lateraloccipital_part6 | 187 |  | rh_lateraloccipital_part2 | |
|  | 38 |  | lh_lateraloccipital_part7 | 188 |  | rh_lateraloccipital_part3 | |
|  | 39 |  | lh_lateraloccipital_part8 | 189 |  | rh_lateraloccipital_part4 | |

| **Yeo functional network** | **Label_ID** | **Hemisphere** | **Cortical region** | **Label_ID** | **Hemisphere** | **Cortical region** |
| --- | --- | --- | --- | --- | --- | --- |
| **VIS** | 40 | Left hemisphere | lh_lateraloccipital_part9 | 190 | Right hemisphere | rh_lateraloccipital_part5 |
|  | 45 |  | lh_lingual_part1 | 191 |  | rh_lateraloccipital_part6 |
|  | 46 |  | lh_lingual_part2 | 192 |  | rh_lateraloccipital_part7 |
|  | 47 |  | lh_lingual_part3 | 193 |  | rh_lateraloccipital_part8 |
|  | 48 |  | lh_lingual_part4 | 194 |  | rh_lateraloccipital_part9 |
|  | 49 |  | lh_lingual_part5 | 199 |  | rh_lingual_part1 |
|  | 50 |  | lh_lingual_part6 | 200 |  | rh_lingual_part2 |
|  | 60 |  | lh_parahippocampal_part2 | 201 |  | rh_lingual_part3 |
|  | 70 |  | lh_pericalcarine_part1 | 202 |  | rh_lingual_part4 |
|  | 71 |  | lh_pericalcarine_part2 | 203 |  | rh_lingual_part5 |
|  | 123 |  | lh_superiorparietal_part2 | 204 |  | rh_lingual_part6 |
|  | 127 |  | lh_superiorparietal_part6 | 214 |  | rh_parahippocampal_part1 |
|  | - |  | - | 226 |  | rh_pericalcarine_part1 |
|  | - |  | - | 227 |  | rh_pericalcarine_part2 |
|  | - |  | - | 228 |  | rh_pericalcarine_part3 |
|  | - |  | - | 248 |  | rh_precuneus_part1 |
|  | - |  | - | 251 |  | rh_precuneus_part4 |
|  | - |  | - | 279 |  | rh_superiorparietal_part1 |
|  | - |  | - | 281 |  | rh_superiorparietal_part3 |
|  | - |  | - | 282 |  | rh_superiorparietal_part4 |

Abbreviations: DAN, dorsal attention network; DMN, default mode network; FPN, fronto-parietal network; rh, right hemisphere; lh, left hemisphere; LN, limbic network; SMN, somato-motor network; VAN, ventral attention network; VIS, visual network.

**Table S4.** Desikan–Killiany (DK308) atlas parcellation with corresponding von Economo class labels.

| **von Economo class** | **Label_ID** | **Hemisphere** | **Cortical region** | **Label_ID** | **Hemisphere** | **Cortical region** |
| --- | --- | --- | --- | --- | --- | --- |
| **Asso 1** | 4 | Left hemisphere | lh_caudalmiddlefrontal_part1 | 156 | Right hemisphere | rh_caudalmiddlefrontal_part1 |
|  | 5 |  | lh_caudalmiddlefrontal_part2 | 157 |  | rh_caudalmiddlefrontal_part2 |
|  | 6 |  | lh_caudalmiddlefrontal_part3 | 158 |  | rh_caudalmiddlefrontal_part3 |
|  | 7 |  | lh_caudalmiddlefrontal_part4 | 159 |  | rh_caudalmiddlefrontal_part4 |
|  | 11 |  | lh_fusiform_part1 | 164 |  | rh_fusiform_part1 |
|  | 12 |  | lh_fusiform_part2 | 165 |  | rh_fusiform_part2 |
|  | 13 |  | lh_fusiform_part3 | 166 |  | rh_fusiform_part3 |
|  | 14 |  | lh_fusiform_part4 | 167 |  | rh_fusiform_part4 |
|  | 15 |  | lh_fusiform_part5 | 168 |  | rh_fusiform_part5 |
|  | 24 |  | lh_inferiortemporal_part1 | 179 |  | rh_inferiortemporal_part1 |
|  | 25 |  | lh_inferiortemporal_part2 | 180 |  | rh_inferiortemporal_part2 |
|  | 26 |  | lh_inferiortemporal_part3 | 181 |  | rh_inferiortemporal_part3 |
|  | 27 |  | lh_inferiortemporal_part4 | 182 |  | rh_inferiortemporal_part4 |
|  | 28 |  | lh_inferiortemporal_part5 | 183 |  | rh_inferiortemporal_part5 |
|  | 29 |  | lh_inferiortemporal_part6 | 208 |  | rh_middletemporal_part1 |
|  | 54 |  | lh_middletemporal_part1 | 209 |  | rh_middletemporal_part2 |
|  | 55 |  | lh_middletemporal_part2 | 210 |  | rh_middletemporal_part3 |
|  | 56 |  | lh_middletemporal_part3 | 211 |  | rh_middletemporal_part4 |
|  | 57 |  | lh_middletemporal_part4 | 212 |  | rh_middletemporal_part5 |

| **von Economo class** | **Label_ID** | **Hemisphere** | **Cortical region** | **Label_ID** | **Hemisphere** | **Cortical region** |
| --- | --- | --- | --- | --- | --- | --- |
| **Asso 1** | 58 | Left hemisphere | lh_middletemporal_part5 | 213 | Right hemisphere | rh_middletemporal_part6 |
|  | 64 |  | lh_parsopercularis_part1 | 219 |  | rh_parsopercularis_part1 |
|  | 65 |  | lh_parsopercularis_part2 | 220 |  | rh_parsopercularis_part2 |
|  | 66 |  | lh_parsopercularis_part3 | 221 |  | rh_parsopercularis_part3 |
|  | 68 |  | lh_parstriangularis_part1 | 223 |  | rh_parstriangularis_part1 |
|  | 69 |  | lh_parstriangularis_part2 | 224 |  | rh_parstriangularis_part2 |
|  | 91 |  | lh_precuneus_part1 | 225 |  | rh_parstriangularis_part3 |
|  | 92 |  | lh_precuneus_part2 | 248 |  | rh_precuneus_part1 |
|  | 93 |  | lh_precuneus_part3 | 249 |  | rh_precuneus_part2 |
|  | 94 |  | lh_precuneus_part4 | 250 |  | rh_precuneus_part3 |
|  | 95 |  | lh_precuneus_part5 | 251 |  | rh_precuneus_part4 |
|  | 96 |  | lh_precuneus_part6 | 252 |  | rh_precuneus_part5 |
|  | 97 |  | lh_precuneus_part7 | 253 |  | rh_precuneus_part6 |
|  | 109 |  | lh_superiorfrontal_part1 | 254 |  | rh_precuneus_part7 |
|  | 110 |  | lh_superiorfrontal_part2 | 266 |  | rh_superiorfrontal_part1 |
|  | 111 |  | lh_superiorfrontal_part3 | 267 |  | rh_superiorfrontal_part2 |
|  | 112 |  | lh_superiorfrontal_part4 | 268 |  | rh_superiorfrontal_part3 |
|  | 113 |  | lh_superiorfrontal_part5 | 269 |  | rh_superiorfrontal_part4 |
|  | 114 |  | lh_superiorfrontal_part6 | 270 |  | rh_superiorfrontal_part5 |

| **von Economo class** | **Label_ID** | **Hemisphere** | **Cortical region** | **Label_ID** | **Hemisphere** | **Cortical region** |
| --- | --- | --- | --- | --- | --- | --- |
| **Asso 1** | 115 | Left hemisphere | lh_superiorfrontal_part7 | 271 | Right hemisphere | rh_superiorfrontal_part6 |
|  | 116 |  | lh_superiorfrontal_part8 | 272 |  | rh_superiorfrontal_part7 |
|  | 117 |  | lh_superiorfrontal_part9 | 273 |  | rh_superiorfrontal_part8 |
|  | 118 |  | lh_superiorfrontal_part10 | 274 |  | rh_superiorfrontal_part9 |
|  | 119 |  | lh_superiorfrontal_part11 | 275 |  | rh_superiorfrontal_part10 |
|  | 120 |  | lh_superiorfrontal_part12 | 276 |  | rh_superiorfrontal_part11 |
|  | 121 |  | lh_superiorfrontal_part13 | 277 |  | rh_superiorfrontal_part12 |
|  | 122 |  | lh_superiorparietal_part1 | 278 |  | rh_superiorfrontal_part13 |
|  | 123 |  | lh_superiorparietal_part2 | 279 |  | rh_superiorparietal_part1 |
|  | 124 |  | lh_superiorparietal_part3 | 280 |  | rh_superiorparietal_part2 |
|  | 125 |  | lh_superiorparietal_part4 | 281 |  | rh_superiorparietal_part3 |
|  | 126 |  | lh_superiorparietal_part5 | 282 |  | rh_superiorparietal_part4 |
|  | 127 |  | lh_superiorparietal_part6 | 283 |  | rh_superiorparietal_part5 |
|  | 128 |  | lh_superiorparietal_part7 | 284 |  | rh_superiorparietal_part6 |
|  | 129 |  | lh_superiorparietal_part8 | 285 |  | rh_superiorparietal_part7 |
|  | 130 |  | lh_superiorparietal_part9 | 286 |  | rh_superiorparietal_part8 |
|  | 131 |  | lh_superiorparietal_part10 | 287 |  | rh_superiorparietal_part9 |
|  | 147 |  | lh_temporalpole_part1 | 288 |  | rh_superiorparietal_part10 |
|  | - |  | - | 303 |  | rh_temporalpole_part1 |

| **von Economo class** | **Label_ID** | **Hemisphere** | **Cortical region** | **Label_ID** | **Hemisphere** | **Cortical region** |
| --- | --- | --- | --- | --- | --- | --- |
| **Asso 2** | 2 | Left hemisphere | lh_bankssts_part2 | 153 | Right hemisphere | rh_bankssts_part1 |
|  | 16 |  | lh_inferiorparietal_part1 | 154 |  | rh_bankssts_part2 |
|  | 17 |  | lh_inferiorparietal_part2 | 169 |  | rh_inferiorparietal_part1 |
|  | 18 |  | lh_inferiorparietal_part3 | 170 |  | rh_inferiorparietal_part2 |
|  | 19 |  | lh_inferiorparietal_part4 | 171 |  | rh_inferiorparietal_part3 |
|  | 20 |  | lh_inferiorparietal_part5 | 172 |  | rh_inferiorparietal_part4 |
|  | 21 |  | lh_inferiorparietal_part6 | 173 |  | rh_inferiorparietal_part5 |
|  | 22 |  | lh_inferiorparietal_part7 | 174 |  | rh_inferiorparietal_part6 |
|  | 23 |  | lh_inferiorparietal_part8 | 175 |  | rh_inferiorparietal_part7 |
|  | 99 |  | lh_rostralmiddlefrontal_part1 | 176 |  | rh_inferiorparietal_part8 |
|  | 100 |  | lh_rostralmiddlefrontal_part2 | 177 |  | rh_inferiorparietal_part9 |
|  | 101 |  | lh_rostralmiddlefrontal_part3 | 178 |  | rh_inferiorparietal_part10 |
|  | 102 |  | lh_rostralmiddlefrontal_part4 | 256 |  | rh_rostralmiddlefrontal_part1 |
|  | 103 |  | lh_rostralmiddlefrontal_part5 | 257 |  | rh_rostralmiddlefrontal_part2 |
|  | 104 |  | lh_rostralmiddlefrontal_part6 | 258 |  | rh_rostralmiddlefrontal_part3 |
|  | 105 |  | lh_rostralmiddlefrontal_part7 | 259 |  | rh_rostralmiddlefrontal_part4 |
|  | 106 |  | lh_rostralmiddlefrontal_part8 | 260 |  | rh_rostralmiddlefrontal_part5 |
|  | 107 |  | lh_rostralmiddlefrontal_part9 | 261 |  | rh_rostralmiddlefrontal_part6 |
|  | 108 |  | lh_rostralmiddlefrontal_part10 | 262 |  | rh_rostralmiddlefrontal_part7 |

| **von Economo class** | **Label_ID** | **Hemisphere** | **Cortical region** | **Label_ID** | **Hemisphere** | **Cortical region** |
| --- | --- | --- | --- | --- | --- | --- |
| **Asso 2** | 132 | Left hemisphere | lh_superiortemporal_part1 | 263 | Right hemisphere | rh_rostralmiddlefrontal_part8 |
|  | 133 |  | lh_superiortemporal_part2 | 264 |  | rh_rostralmiddlefrontal_part9 |
|  | 134 |  | lh_superiortemporal_part3 | 265 |  | rh_rostralmiddlefrontal_part10 |
|  | 135 |  | lh_superiortemporal_part4 | 289 |  | rh_superiortemporal_part1 |
|  | 136 |  | lh_superiortemporal_part5 | 290 |  | rh_superiortemporal_part2 |
|  | 137 |  | lh_superiortemporal_part6 | 291 |  | rh_superiortemporal_part3 |
|  | 138 |  | lh_superiortemporal_part7 | 292 |  | rh_superiortemporal_part4 |
|  | 139 |  | lh_supramarginal_part1 | 293 |  | rh_superiortemporal_part5 |
|  | 140 |  | lh_supramarginal_part2 | 294 |  | rh_superiortemporal_part6 |
|  | 141 |  | lh_supramarginal_part3 | 295 |  | rh_supramarginal_part1 |
|  | 142 |  | lh_supramarginal_part4 | 296 |  | rh_supramarginal_part2 |
|  | 143 |  | lh_supramarginal_part5 | 297 |  | rh_supramarginal_part3 |
|  | 144 |  | lh_supramarginal_part6 | 298 |  | rh_supramarginal_part4 |
|  | 145 |  | lh_supramarginal_part7 | 299 |  | rh_supramarginal_part5 |
|  | 146 |  | lh_frontalpole_part1 | 300 |  | rh_supramarginal_part6 |
|  | - |  | - | 301 |  | rh_supramarginal_part7 |
|  | - |  | - | 302 |  | rh_frontalpole_part1 |
|  | - |  | - | - |  | - |
|  | - |  | - | - |  | - |

| **von Economo class** | **Label_ID** | **Hemisphere** | **Cortical region** | **Label_ID** | **Hemisphere** | **Cortical region** |
| --- | --- | --- | --- | --- | --- | --- |
| **Insula** | 149 | Left hemisphere | lh_insula_part1 | 305 | Right hemisphere | rh_insula_part1 |
|  | 150 |  | lh_insula_part2 | 306 |  | rh_insula_part2 |
|  | 151 |  | lh_insula_part3 | 307 |  | rh_insula_part3 |
|  | 152 |  | lh_insula_part4 | 308 |  | rh_insula_part4 |
| **Limbic** | 3 |  | lh_caudalanteriorcingulate_part1 | 155 |  | rh_caudalanteriorcingulate_part1 |
|  | 10 |  | lh_entorhinal_part1 | 163 |  | rh_entorhinal_part1 |
|  | 30 |  | lh_isthmuscingulate_part1 | 184 |  | rh_isthmuscingulate_part1 |
|  | 31 |  | lh_isthmuscingulate_part2 | 185 |  | rh_isthmuscingulate_part2 |
|  | 59 |  | lh_parahippocampal_part1 | 214 |  | rh_parahippocampal_part1 |
|  | 60 |  | lh_parahippocampal_part2 | 215 |  | rh_parahippocampal_part2 |
|  | 80 |  | lh_posteriorcingulate_part1 | 237 |  | rh_posteriorcingulate_part1 |
|  | 81 |  | lh_posteriorcingulate_part2 | 238 |  | rh_posteriorcingulate_part2 |
|  | 98 |  | lh_rostralanteriorcingulate_part1 | 255 |  | rh_rostralanteriorcingulate_part1 |
| **Prim motor** | 61 |  | lh_paracentral_part1 | 216 |  | rh_paracentral_part1 |
|  | 62 |  | lh_paracentral_part2 | 217 |  | rh_paracentral_part2 |
|  | 63 |  | lh_paracentral_part3 | 218 |  | rh_paracentral_part3 |
|  | 82 |  | lh_precentral_part1 | 239 |  | rh_precentral_part1 |
|  | 83 |  | lh_precentral_part2 | 240 |  | rh_precentral_part2 |
|  | 84 |  | lh_precentral_part3 | 241 |  | rh_precentral_part3 |

| **von Economo class** | **Label_ID** | **Hemisphere** | **Cortical region** | **Label_ID** | **Hemisphere** | **Cortical region** |
| --- | --- | --- | --- | --- | --- | --- |
| **Prim motor** | 85 | Left hemisphere | lh_precentral_part4 | 242 | Right hemisphere | rh_precentral_part4 |
|  | 86 |  | lh_precentral_part5 | 243 |  | rh_precentral_part5 |
|  | 87 |  | lh_precentral_part6 | 244 |  | rh_precentral_part6 |
|  | 88 |  | lh_precentral_part7 | 245 |  | rh_precentral_part7 |
|  | 89 |  | lh_precentral_part8 | 246 |  | rh_precentral_part8 |
|  | 90 |  | lh_precentral_part9 | 247 |  | rh_precentral_part9 |
|  | - |  | - | - |  | - |
|  | - |  | - | - |  | - |
|  | - |  | - | - |  | - |
| **Prim sens** | 70 |  | lh_pericalcarine_part1 | 226 |  | rh_pericalcarine_part1 |
|  | 71 |  | lh_pericalcarine_part2 | 227 |  | rh_pericalcarine_part2 |
|  | 72 |  | lh_postcentral_part1 | 228 |  | rh_pericalcarine_part3 |
|  | 73 |  | lh_postcentral_part2 | 229 |  | rh_postcentral_part1 |
|  | 74 |  | lh_postcentral_part3 | 230 |  | rh_postcentral_part2 |
|  | 75 |  | lh_postcentral_part4 | 231 |  | rh_postcentral_part3 |
|  | 76 |  | lh_postcentral_part5 | 232 |  | rh_postcentral_part4 |
|  | 77 |  | lh_postcentral_part6 | 233 |  | rh_postcentral_part5 |
|  | 78 |  | lh_postcentral_part7 | 234 |  | rh_postcentral_part6 |
|  | 79 |  | lh_postcentral_part8 | 235 |  | rh_postcentral_part7 |

| **von Economo class** | **Label_ID** | **Hemisphere** | **Cortical region** | **Label_ID** | **Hemisphere** | **Cortical region** |
| --- | --- | --- | --- | --- | --- | --- |
| **Prim sens** | 148 | Left hemisphere | lh_transversetemporal_part1 | 236 | Right hemisphere | rh_postcentral_part8 |
|  | - |  | - | 304 |  | rh_transversetemporal_part1 |
| **Sec sens** | 8 |  | lh_cuneus_part1 | 160 |  | rh_cuneus_part1 |
|  | 9 |  | lh_cuneus_part2 | 161 |  | rh_cuneus_part2 |
|  | 32 |  | lh_lateraloccipital_part1 | 162 |  | rh_cuneus_part3 |
|  | 33 |  | lh_lateraloccipital_part2 | 186 |  | rh_lateraloccipital_part1 |
|  | 34 |  | lh_lateraloccipital_part3 | 187 |  | rh_lateraloccipital_part2 |
|  | 35 |  | lh_lateraloccipital_part4 | 188 |  | rh_lateraloccipital_part3 |
|  | 36 |  | lh_lateraloccipital_part5 | 189 |  | rh_lateraloccipital_part4 |
|  | 37 |  | lh_lateraloccipital_part6 | 190 |  | rh_lateraloccipital_part5 |
|  | 38 |  | lh_lateraloccipital_part7 | 191 |  | rh_lateraloccipital_part6 |
|  | 39 |  | lh_lateraloccipital_part8 | 192 |  | rh_lateraloccipital_part7 |
|  | 40 |  | lh_lateraloccipital_part9 | 193 |  | rh_lateraloccipital_part8 |
|  | 41 |  | lh_lateralorbitofrontal_part1 | 194 |  | rh_lateraloccipital_part9 |
|  | 42 |  | lh_lateralorbitofrontal_part2 | 195 |  | rh_lateralorbitofrontal_part1 |
|  | 43 |  | lh_lateralorbitofrontal_part3 | 196 |  | rh_lateralorbitofrontal_part2 |
|  | 44 |  | lh_lateralorbitofrontal_part4 | 197 |  | rh_lateralorbitofrontal_part3 |
|  | 45 |  | lh_lingual_part1 | 198 |  | rh_lateralorbitofrontal_part4 |
|  | 46 |  | lh_lingual_part2 | 199 |  | rh_lingual_part1 |

| **von Economo class** | **Label_ID** | **Hemisphere** | **Cortical region** | **Label_ID** | **Hemisphere** | **Cortical region** |
| --- | --- | --- | --- | --- | --- | --- |
| **Sec sens** | 47 | Left hemisphere | lh_lingual_part3 | 200 | Right hemisphere | rh_lingual_part2 |
|  | 48 |  | lh_lingual_part4 | 201 |  | rh_lingual_part3 |
|  | 49 |  | lh_lingual_part5 | 202 |  | rh_lingual_part4 |
|  | 50 |  | lh_lingual_part6 | 203 |  | rh_lingual_part5 |
|  | 51 |  | lh_medialorbitofrontal_part1 | 204 |  | rh_lingual_part6 |
|  | 52 |  | lh_medialorbitofrontal_part2 | 205 |  | rh_medialorbitofrontal_part1 |
|  | 53 |  | lh_medialorbitofrontal_part3 | 206 |  | rh_medialorbitofrontal_part2 |
|  | 67 |  | lh_parsorbitalis_part1 | 207 |  | rh_medialorbitofrontal_part3 |
|  | - |  | - | 222 |  | rh_parsorbitalis_part1 |

Abbreviations: Asso1, association cortex1; Asso2, association cortex2; Insula, insular cortex; lh, left hemisphere; Limbic, limbic regions; Prim motor, primary motor cortex; Prim sens, primary sensory cortex; rh, right hemisphere; Sec sens, second sensory cortex.

**Table S5.** Regional MIND gradient differences between BD and healthy controls.

| **Regions** | **MNI coordinates (x, y, z)** | | | ***t*-statistic** | ***P* value** |
| --- | --- | --- | --- | --- | --- |
| lh_lateraloccipital_part8 | -24 | -93 | -13 | 3.91 | 2.04 × 10^-2^ |
| lh_rostralmiddlefrontal_part4 | -22 | 58 | -10 | 4.17 | 1.50 × 10^-2^ |

GLM was used to investigate regionally principal MIND gradient alterations in BD group, while regressing out the effect of age, sex, education years, and age × sex interaction. The *t*-statistic > 0 means BD > healthy controls. All *P* values survived after BH-FDR correction with *P* < 0.05. Abbreviations: BD, bipolar disorder; BH-FDR, Benjamini-Hochberg false discovery rate; GLM, general linear model; lh, left hemisphere; MIND, Morphometric Inverse Divergence; MNI, Montreal Neurological Institute.

**Table S6.** The differences in the principal MIND gradient in each Yeo functional network.

| **Statistics** | **DAN** | **DMN** | **FPN** | **LN** | **SMN** | **VAN** | **VIS** |
| --- | --- | --- | --- | --- | --- | --- | --- |
| *t*-statistic | 0.23 | -1.54 | 3.24 | 0.73 | -2.11 | -3.47 | 2.84 |
| *P* value | 2.66 × 10^-1^ | 1.77 × 10^-1^ | 5.14 × 10^-3**^ | 5.46 × 10^-1^ | 6.44 × 10^-2^ | 4.72 × 10^-3**^ | 1.19 × 10^-2*^ |

GLM was used to investigate regionally principal MIND gradient alterations in BD group, while regressing out the effect of age, sex, education years, and age × sex interaction. The *t*-statistic > 0 means BD > healthy controls, The *t*-statistic < 0 means BD < healthy controls. All *P* values survived after BH-FDR correction with *P* < 0.05. ^*^Indicates that the *P* value < 0.05, and ^**^ indicates that the *P* value < 0.01. Abbreviations: BD, bipolar disorder; BH-FDR, Benjamini-Hochberg false discovery rate; DAN, dorsal attention network; DMN, default mode network; FPN, fronto-parietal network; GLM, general linear model; LN, limbic network; MIND, Morphometric Inverse Divergence; SMN, somato-motor network; VAN, ventral attention network; VIS, visual network.

**Table S7.** The differences in the principal MIND gradient in each von Economo class.

| **Statistics** | **Asso 1** | **Asso 2** | **Insula** | **Limbic** | **Prim motor** | **Prim sens** | **Sec sens** |
| --- | --- | --- | --- | --- | --- | --- | --- |
| *t*-statistic | -1.60 | 2.23 | -0.66 | -2.21 | -2.54 | 0.68 | 3.14 |
| *P* value | 1.57 × 10^-1^ | 5.02 × 10^-2^ | 5.12 × 10^-1^ | 5.02 × 10^-2^ | 4.20 × 10^-2*^ | 5.13 × 10^-1^ | 1.41 × 10^-2*^ |

GLM was used to investigate regionally principal MIND gradient alterations in BD group, while regressing out the effect of age, sex, education years, and age × sex interaction. The *t*-statistic > 0 means BD > healthy controls, The *t*-statistic < 0 means BD < healthy controls. All *P* values survived after BH-FDR correction with *P* < 0.05. ^*^Indicates that the *P* value < 0.05. Abbreviations: Asso1, association cortex1; Asso2, association cortex2; BD, bipolar disorder; BH-FDR, Benjamini-Hochberg false discovery rate; GLM, general linear model; Insula, insular cortex; Limbic, limbic regions; MIND, Morphometric Inverse Divergence; Prim motor, primary motor cortex; Prim sens, primary sensory cortex; Sec sens, second sensory cortex.

**Table S8.**  Neurotransmitter receptors and transporters included in this study.

| **Receptor/transporter** | **Neurotransmitter** | **Tracer** | **Modality** | **Subjects** | **Source and reference** |
| --- | --- | --- | --- | --- | --- |
| *α_4_β_2_* | acetylcholine | (^18^F) ﬂubatine | PET | 30 | (Hillmer et al., 2016) |
| M_1_ | acetylcholine | (^11^C) LSN3172176 | PET | 24 | (Naganawa et al., 2021) |
| VAChT | acetylcholine | (^18^F) FEOBV | PET | 4 | PI: Lauri Tuominen & Synthia Guimond |
|  |  |  |  | 18 | (Aghourian et al., 2017) |
|  |  |  |  | 5 | (Bedard et al., 2019) |
|  |  |  |  | 3 | PI: Taylor W. Schmitz & R. Nathan Spreng |
| CB_1_ | cannabinoid | (^11^C) OMAR | PET | 77 | (Normandin et al., 2015) |
| DAT | dopamine | (^123^I)-FP-CIT | SPECT | 174 | (Dukart et al., 2018) |
| D_1_ | dopamine | (^11^C) SCH23390 | PET | 13 | (Kaller et al., 2017) |
| D_2_ | dopamine | (^11^C) FLB-457 | PET | 37 | (Smith et al., 2019) |
| GABA_A/BZ_ | GABA | (^11^C) ﬂumazenil | PET | 16 | (Nørgaard et al., 2021) |
| mGluR_5_ | glutamate | (^11^C) ABP688 | PET | 73 | (Smart et al., 2019) |
|  |  |  |  | 22 | PI: Pedro Rosa-Neto |
|  |  |  |  | 28 | (DuBois et al., 2016) |
| NMDA | glutamate | (^18^F) GE-179 | PET | 29 | (Galovic et al., 2021) |
| H_3_ | histamine | (^11^C) GSK189254 | PET | 8 | (Gallezot et al., 2017) |
| NET | norepinephrine | (^11^C) MRB | PET | 77 | (Ding et al., 2010) |
| MOR | opioid | (^11^C) carfentanil | PET | 204 | (Kantonen et al., 2020) |
| 5-HT_1A_ | serotonin | (^11^C) WAY-100635 | PET | 36 | (Savli et al., 2012) |
| 5-HT_1B_ | serotonin | (^11^C) P943 | PET | 65 | (Gallezot et al., 2010) |
|  |  |  |  | 23 | (Gallezot et al., 2010) |
| 5-HT_2A_ | serotonin | (^11^C) Cimbi-36 | PET | 29 | (Beliveau et al., 2017) |
| 5-HT_4_ | serotonin | (^11^C) SB207145 | PET | 59 | (Beliveau et al., 2017) |
| 5-HT_6_ | serotonin | (^11^C) GSK215083 | PET | 30 | (Radhakrishnan et al., 2020; Radhakrishnan et al., 2018) |
| 5-HTT | serotonin | (^11^C) DASB | PET | 100 | (Beliveau et al., 2017) |

Abbreviations: *α_4_β_2_*, nicotinic acetylcholine receptors; 5-HT, 5-hydroxytryptamine (serotonin); CB1, cannabinoid type 1; D, dopamine; DAT, dopamine transporter; GABA_A/BZ_, gamma-aminobutyric acid A/BZ; H_3_, histamine H_3_ receptor; mGluR_5_, metabotropic glutamate type 5; M_1_, muscarinic acetylcholine receptor M_1_; MOR, mu opioid receptor; NAT, noradrenaline transporter; NET, norepinephrine transporter; NMDA, N-methyl-D-aspartate receptor; PET, positron emission tomography; SPECT, single photon emission computed tomography; VAChT, vesicular acetylcholine transporter.

**Table S9.** Association of neurotransmitter systems with the principal MIND gradient alterations.

| **Receptor/transporter** | **Spatial correlation**  **(Pearson’s coefficient)** | | ***P_spin_*** | **-log_10_(*P_spin_*)** | **Bonferroni *P* value** |
| --- | --- | --- | --- | --- | --- |
| *α_4_β_2_* | -0.19 | 4.80 × 10^-2^ | | 1.32 | 9.18 × 10^-1^ |
| M_1_ | -0.01 | 9.00 × 10^-1^ | | 0.05 | 1.00 |
| VAChT | -0.39 | 2.00 × 10^-4^ | | 3.70 | 3.80 × 10^-3^ |
| CB_1_ | -0.08 | 4.58 × 10^-1^ | | 0.34 | 1.00 |
| DAT | -0.18 | 1.00 × 10^-1^ | | 1.00 | 1.00 |
| D_1_ | -0.05 | 7.33 × 10^-1^ | | 0.14 | 1.00 |
| D_2_ | -0.10 | 3.92 × 10^-1^ | | 0.41 | 1.00 |
| GABA_A/BZ_ | 0.30 | 6.00 × 10^-4^ | | 3.22 | 1.14 × 10^-2^ |
| mGluR_5_ | -0.17 | 1.54 × 10^-2^ | | 0.81 | 1.00 |
| NMDA | -0.07 | 4.10 × 10^-1^ | | 0.39 | 1.00 |
| H_3_ | -0.22 | 5.20 × 10^-2^ | | 1.28 | 9.96 × 10^-1^ |
| NET | -0.24 | 1.80 × 10^-2^ | | 1.76 | 3.32 × 10^-1^ |
| MOR | -0.27 | 6.50 × 10^-2^ | | 1.18 | 1.00 |
| 5-HT_1A_ | -0.15 | 2.94 × 10^-1^ | | 0.53 | 1.00 |
| 5-HT_1B_ | 0.19 | 9.20 × 10^-2^ | | 1.03 | 1.00 |
| 5-HT_2A_ | 0.12 | 2.17 × 10^-1^ | | 0.67 | 1.00 |
| 5-HT_4_ | -0.06 | 5.39 × 10^-1^ | | 0.27 | 1.00 |
| 5-HT_6_ | -0.01 | 8.66 × 10^-1^ | | 0.06 | 1.00 |
| 5-HTT | -0.08 | 4.77 × 10^-1^ | | 0.32 | 1.00 |

Spatial correlations between the case-control *t*-map of the principal MIND gradient and each neurotransmitter receptor map were assessed. Statistical significance was determined using 10,000 spin tests, with Bonferroni correction applied to account for multiple comparisons across 19 different maps. All *P* values remained significant after Bonferroni correction (*P* < 0.05). Abbreviations: *α_4_β_2_*, nicotinic acetylcholine receptors; 5-HT, 5-hydroxytryptamine (serotonin); CB1, cannabinoid type 1; D, dopamine; DAT, dopamine transporter; GABA_A/BZ_, gamma-aminobutyric acid A/BZ; H_3_, histamine H_3_ receptor; mGluR_5_, metabotropic glutamate type 5; M1, muscarinic acetylcholine receptor M1; MIND, Morphometric Inverse Divergence; MOR, mu opioid receptor; NAT, noradrenaline transporter; NET, norepinephrine transporter; NMDA, N-methyl-D-aspartate receptor; PET, positron emission tomography; SPECT, single photon emission computed tomography; VAChT, vesicular acetylcholine transporter.

**Table S10.** Cognitive-behavioral terms

| **terms** | **terms** | **terms** | **terms** | **terms** |
| --- | --- | --- | --- | --- |
| action | eating | insight | naming | semantic memory |
| adaptation | efficiency | integration | navigation | sentence comprehension |
| addiction | effort | intelligence | object recognition | skill |
| anticipation | emotion | intention | pain | sleep |
| anxiety | emotion regulation | interference | perception | social cognition |
| arousal | empathy | judgment | planning | spatial attention |
| association | encoding | knowledge | priming | speech perception |
| attention | episodic memory | language | psychosis | speech production |
| autobiographical memory | expectancy | language comprehension | reading | strategy |
| balance | expertise | learning | reasoning | strength |
| belief | extinction | listening | recall | stress |
| categorization | face recognition | localization | recognition | sustained attention |
| cognitive control | facial expression | loss | rehearsal | task difficulty |
| communication | familiarity | maintenance | reinforcement learning | thought |
| competition | fear | manipulation | response inhibition | timing |
| concept | fixation | meaning | response selection | transition |
| consciousness | focus | memory | retention | uncertainty |
| consolidation | gaze | memory retrieval | retrieval | updating |
| context | goal | mental imagery | reward anticipation | utility |
| coordination | hyperactivity | monitoring | rhythm | valence |
| decision | imagery | mood | risk | verbal fluency |
| decision making | impulsivity | morphology | rule | visual attention |
| detection | induction | motor control | salience | visual perception |
| discrimination | inference | movement | search | word recognition |
| distraction | inhibition | multisensory | selective attention | working memory |

A total of 125 terms that overlapped between the Neurosynth database (Yarkoni, Poldrack, Nichols, Van Essen, & Wager, 2011) and the Cognitive Atlas (<https://cognitiveatlas.org/>) (Poldrack et al., 2011) were included in the analysis.

**Table S11.** The association of cognitive-behavioral processes with the principal MIND gradient alterations.

| **terms** | **PLS weight** | ***Z*-score** | **FDR *P* value** |
| --- | --- | --- | --- |
| action | -7.30 × 10^-4^ | -0.95 | 4.91 × 10^-1^ |
| adaptation | 7.54 × 10^-4^ | 0.92 | 5.05 × 10^-1^ |
| addiction | -9.70 × 10^-4^ | -1.13 | 4.28 × 10^-1^ |
| anticipation | -1.14 × 10^-3^ | -1.26 | 3.53 × 10^-1^ |
| anxiety | -8.20 × 10^-4^ | -0.98 | 4.73 × 10^-1^ |
| arousal | -2.38 × 10^-3^ | -2.72 | 2.91 × 10^-2*^ |
| association | -2.20 × 10^-4^ | -0.26 | 8.57 × 10^-1^ |
| attention | 1.65 × 10^-3^ | 2.34 | 6.64 × 10^-2^ |
| autobiographical memory | 7.43 × 10^-5^ | 0.08 | 9.59 × 10^-1^ |
| balance | -1.62 × 10^-3^ | -1.89 | 1.39 × 10^-1^ |
| belief | 1.03 × 10^-3^ | 1.34 | 3.30 × 10^-1^ |
| categorization | -3.10 × 10^-4^ | -0.36 | 7.99 × 10^-1^ |
| cognitive control | -2.00 × 10^-4^ | -0.20 | 8.80 × 10^-1^ |
| communication | -2.16 × 10^-3^ | -2.57 | 4.19 × 10^-2*^ |
| competition | 1.88 × 10^-3^ | 2.09 | 9.74 × 10^-2^ |
| concept | 9.33 × 10^-5^ | 0.11 | 9.49 × 10^-1^ |
| consciousness | 4.58 × 10^-4^ | 0.52 | 7.26 × 10^-1^ |
| consolidation | -2.22 × 10^-3^ | -2.23 | 8.36 × 10^-2^ |
| context | -1.18 × 10^-3^ | -1.42 | 3.03 × 10^-1^ |
| coordination | -2.53 × 10^-3^ | -3.13 | 1.03 × 10^-2*^ |
| decision | 2.80 × 10^-3^ | 3.27 | 7.49 × 10^-3**^ |
| decision making | 3.37 × 10^-3^ | 4.02 | 8.93 × 10^-4***^ |
| detection | 1.84 × 10^-3^ | 1.86 | 1.46 × 10^-1^ |
| discrimination | -1.16 × 10^-3^ | -1.34 | 3.30 × 10^-1^ |
| distraction | -7.10 × 10^-4^ | -0.72 | 6.05 × 10^-1^ |
| eating | 2.22 × 10^-3^ | 2.46 | 5.44 × 10^-2^ |
| efficiency | 1.63 × 10^-3^ | 2.00 | 1.17 × 10^-1^ |
| effort | -2.11 × 10^-3^ | -2.37 | 6.31 × 10^-2^ |
| emotion | -7.70 × 10^-4^ | -1.08 | 4.45 × 10^-1^ |
| emotion regulation | -3.50 × 10^-4^ | -0.42 | 7.70 × 10^-1^ |
| empathy | -3.30 × 10^-3^ | -3.40 | 5.11 × 10^-3**^ |
| encoding | 1.43 × 10^-3^ | 1.79 | 1.63 × 10^-1^ |
| episodic memory | 4.54 × 10^-4^ | 0.55 | 7.09 × 10^-1^ |
| expectancy | 1.09 × 10^-3^ | 1.31 | 3.34 × 10^-1^ |
| expertise | 1.97 × 10^-3^ | 2.17 | 8.83 × 10^-2^ |
| extinction | 7.62 × 10^-4^ | 0.90 | 5.05 × 10^-1^ |
| face recognition | 1.47 × 10^-3^ | 2.14 | 9.24 × 10^-2^ |
| facial expression | 4.96 × 10^-4^ | 0.65 | 6.44 × 10^-1^ |
| familiarity | 9.16 × 10^-4^ | 1.09 | 4.45 × 10^-1^ |
| fear | -8.40 × 10^-4^ | -1.08 | 4.45 × 10^-1^ |
| fixation | 3.30 × 10^-3^ | 3.79 | 1.92 × 10^-3**^ |
| focus | -3.00 × 10^-4^ | -0.31 | 8.20 × 10^-1^ |
| gaze | 2.69 × 10^-3^ | 2.89 | 1.94 × 10^-2*^ |
| goal | 1.63 × 10^-3^ | 2.29 | 7.42 × 10^-2^ |
| hyperactivity | -4.10 × 10^-4^ | -0.50 | 7.38 × 10^-1^ |
| imagery | -1.43 × 10^-3^ | -1.84 | 1.51 × 10^-1^ |
| impulsivity | 2.55 × 10^-3^ | 2.77 | 2.63 × 10^-2*^ |
| induction | -2.64 × 10^-3^ | -2.95 | 1.68 × 10^-2*^ |
| inference | -3.20 × 10^-4^ | -0.42 | 7.70 × 10^-1^ |
| inhibition | -1.40 × 10^-3^ | -1.43 | 3.03 × 10^-1^ |
| insight | -8.00 × 10^-4^ | -0.92 | 5.05 × 10^-1^ |
| integration | -1.87 × 10^-3^ | -2.42 | 5.86 × 10^-2^ |
| intelligence | 2.88 × 10^-3^ | 3.02 | 1.43 × 10^-2*^ |
| intention | 3.00 × 10^-3^ | 3.38 | 5.25 × 10^-3**^ |
| interference | 5.45 × 10^-5^ | 0.06 | 9.59 × 10^-1^ |
| judgment | 1.84 × 10^-3^ | 2.47 | 5.38 × 10^-2^ |
| knowledge | 1.46 × 10^-3^ | 1.81 | 1.56 × 10^-1^ |
| language | -1.19 × 10^-3^ | -1.64 | 2.09 × 10^-1^ |
| language comprehension | -2.21 × 10^-3^ | -3.00 | 1.49 × 10^-2*^ |
| learning | 7.62 × 10^-4^ | 0.85 | 5.28 × 10^-1^ |
| listening | -4.43 × 10^-3^ | -6.40 | 1.91 × 10^-8***^ |
| localization | -6.09 × 10^-5^ | -0.06 | 9.59 × 10^-1^ |
| loss | -1.48 × 10^-3^ | -1.94 | 1.29 × 10^-1^ |
| maintenance | 7.42 × 10^-4^ | 0.90 | 5.05 × 10^-1^ |
| manipulation | 1.10 × 10^-3^ | 1.45 | 2.96 × 10^-1^ |
| meaning | -1.42 × 10^-3^ | -1.90 | 1.36 × 10^-1^ |
| memory | 2.14 × 10^-3^ | 3.16 | 1.03 × 10^-2*^ |
| memory retrieval | -3.60 × 10^-4^ | -0.41 | 7.70 × 10^-1^ |
| mental imagery | 1.85 × 10^-3^ | 2.20 | 8.67 × 10^-2^ |
| monitoring | -1.09 × 10^-3^ | -1.05 | 4.48 × 10^-1^ |
| mood | 1.96 × 10^-3^ | 2.40 | 6.04 × 10^-2^ |
| morphology | -2.39 × 10^-3^ | -3.14 | 1.03 × 10^-2*^ |
| motor control | -3.14 × 10^-3^ | -4.15 | 5.84 × 10^-4***^ |
| movement | -2.82 × 10^-3^ | -3.78 | 1.92 × 10^-3**^ |
| multisensory | -6.10 × 10^-4^ | -0.72 | 6.05 × 10^-1^ |
| naming | 2.54 × 10^-4^ | 0.34 | 8.04 × 10^-1^ |
| navigation | 2.21 × 10^-4^ | 0.23 | 8.64 × 10^-1^ |
| object recognition | 3.13 × 10^-3^ | 3.62 | 3.04 × 10^-3**^ |
| pain | -4.24 × 10^-3^ | -4.68 | 6.86 × 10^-5***^ |
| perception | -1.67 × 10^-3^ | -2.18 | 8.83 × 10^-2^ |
| planning | -9.60 × 10^-4^ | -1.26 | 3.53 × 10^-1^ |
| priming | 4.58 × 10^-4^ | 0.61 | 6.66 × 10^-1^ |
| psychosis | -5.40 × 10^-4^ | -0.61 | 6.66 × 10^-1^ |
| reading | 8.37 × 10^-4^ | 1.07 | 4.45 × 10^-1^ |
| reasoning | 2.86 × 10^-3^ | 3.57 | 3.14 × 10^-3**^ |
| recall | -2.00 × 10^-3^ | -1.78 | 1.63 × 10^-1^ |
| recognition | 1.03 × 10^-3^ | 1.38 | 3.24 × 10^-1^ |
| rehearsal | -1.04 × 10^-3^ | -1.32 | 3.34 × 10^-1^ |
| reinforcement learning | 1.86 × 10^-3^ | 2.13 | 9.31 × 10^-2^ |
| response inhibition | -3.90 × 10^-3^ | -0.42 | 7.70 × 10^-1^ |
| response selection | -1.24 × 10^-3^ | -1.33 | 3.34 × 10^-1^ |
| retention | 6.85 × 10^-4^ | 0.70 | 6.11 × 10^-1^ |
| retrieval | 8.12 × 10^-4^ | 1.05 | 4.49 × 10^-1^ |
| reward anticipation | 2.32 × 10^-3^ | 2.67 | 3.31 × 10^-2*^ |
| rhythm | -3.73 × 10^-3^ | -5.10 | 1.09 × 10^-5***^ |
| risk | 1.72 × 10^-3^ | 2.10 | 9.65 × 10^-2^ |
| rule | 2.95 × 10^-3^ | 3.60 | 3.04 × 10^-3**^ |
| salience | 9.57 × 10^-4^ | 1.20 | 3.82 × 10^-1^ |
| search | 3.33 × 10^-3^ | 3.68 | 2.69 × 10^-3**^ |
| selective attention | 6.52 × 10^-4^ | 0.85 | 5.28 × 10^-1^ |
| semantic memory | -8.40 × 10^-4^ | -1.00 | 4.70 × 10^-1^ |
| sentence comprehension | -5.00 × 10^-4^ | -0.71 | 6.08 × 10^-1^ |
| skill | -1.41 × 10^-3^ | -1.56 | 2.45 × 10^-1^ |
| sleep | -6.30 × 10^-4^ | -0.74 | 6.01 × 10^-1^ |
| social cognition | 3.47 × 10^-5^ | 0.05 | 9.64 × 10^-1^ |
| spatial attention | 1.53 × 10^-3^ | 2.15 | 9.15 × 10^-2^ |
| speech perception | -3.57 × 10^-3^ | -5.34 | 5.67 × 10^-6***^ |
| speech production | -3.39 × 10^-3^ | -5.27 | 5.72 × 10^-6***^ |
| strategy | 3.16 × 10^-4^ | 0.36 | 7.99 × 10^-1^ |
| strength | -3.90 × 10^-4^ | -0.41 | 7.70 × 10^-1^ |
| stress | -8.50 × 10^-4^ | -0.99 | 4.70 × 10^-1^ |
| sustained attention | -1.89 × 10^-3^ | -2.05 | 1.06 × 10^-1^ |
| task difficulty | -8.40 × 10^-4^ | -0.85 | 5.28 × 10^-1^ |
| thought | 1.18 × 10^-3^ | 1.35 | 3.31 × 10^-1^ |
| timing | -1.52 × 10^-3^ | -1.98 | 1.19 × 10^-1^ |
| transition | 1.38 × 10^-3^ | 1.65 | 2.09 × 10^-1^ |
| uncertainty | 2.67 × 10^-3^ | 2.88 | 1.94 × 10^-2*^ |
| updating | 2.69 × 10^-3^ | 3.51 | 3.78 × 10^-3**^ |
| utility | -2.10 × 10^-4^ | -0.23 | 8.64 × 10^-1^ |
| valence | -7.50 × 10^-4^ | -1.00 | 4.70 × 10^-1^ |
| verbal fluency | 4.99 × 10^-5^ | 0.06 | 9.59 × 10^-1^ |
| visual attention | 4.43 × 10^-3^ | 4.65 | 6.86 × 10^-5***^ |
| visual perception | 1.01 × 10^-3^ | 1.27 | 3.53 × 10^-1^ |
| word recognition | -4.00 × 10^-4^ | -0.48 | 7.42 × 10^-1^ |
| working memory | 1.66 × 10^-3^ | 2.28 | 7.48 × 10^-2^ |

The PLS weight indicates the direction of the correlation. The *Z*-score for each weight was determined using a bootstrapping procedure (10,000 iterations). All *P* values were corrected for multiple comparisons using the BH-FDR correction method (*P* < 0.05). ^*^Indicates that the *P* value < 0.05, ^**^ indicates that the *P* value < 0.01, and ^***^ indicates that the *P* value < 0.001. Abbreviations: BH-FDR, Benjamini-Hochberg false discovery rate; MIND, Morphometric Inverse Divergence; PLS, Partial Least Squares.

**Table S12.** The six donors’ information in the AHBA database.

| **Donor** | **Age (years)** | **Gender** | **Ethnicity** | **Hemisphere** | **Samples** | **Post-mortem interval (h)** |
| --- | --- | --- | --- | --- | --- | --- |
| H0351.2001 | 24 | Male | African American | Both | 946 | 23 |
| H0351.2002 | 39 | Male | African American | Both | 893 | 10 |
| H0351.1009 | 57 | Male | Caucasian | Left | 363 | 25.5 |
| H0351.1012 | 31 | Male | Caucasian | Left | 529 | 17.5 |
| H0351.1015 | 49 | Female | Hispanic | Left | 470 | 30 |
| H0351.1016 | 55 | Male | Caucasian | Left | 501 | 18 |

Abbreviations: AHBA, Allen Human Brain Atlas.

**Table S13.** BD-related genes from the AHBA database.

| **Gene Symbol** | **Description** | **Gene Family** |
| --- | --- | --- |
| *ADRBK2* | adrenergic, beta, receptor kinase 2 | kinase |
| ***CLOCK^*^*** | clock homolog (mouse) | transcription factor |
| ***XBP1^*^*** | X-box binding protein 1 | transcription factor |

Gene symbols in bold indicate these genes were contained in the list of 15,632 background genes. ^*^Indicates that these genes were significantly correlated with the case-control *t*-map (All *P* values were derived from spin tests and adjusted by the BH-FDR method). Abbreviations: *ADRBK2*, Adrenergic Receptor Kinase Beta 2; AHBA, Allen Human Brain Atlas; BD, bipolar disorder; BH-FDR, Benjamini-Hochberg false discovery rate; *CLOCK*, Circadian Locomotor Output Cycles Kaput; *XBP1*, X-box Binding Protein 1.

**Supplementary Figures**


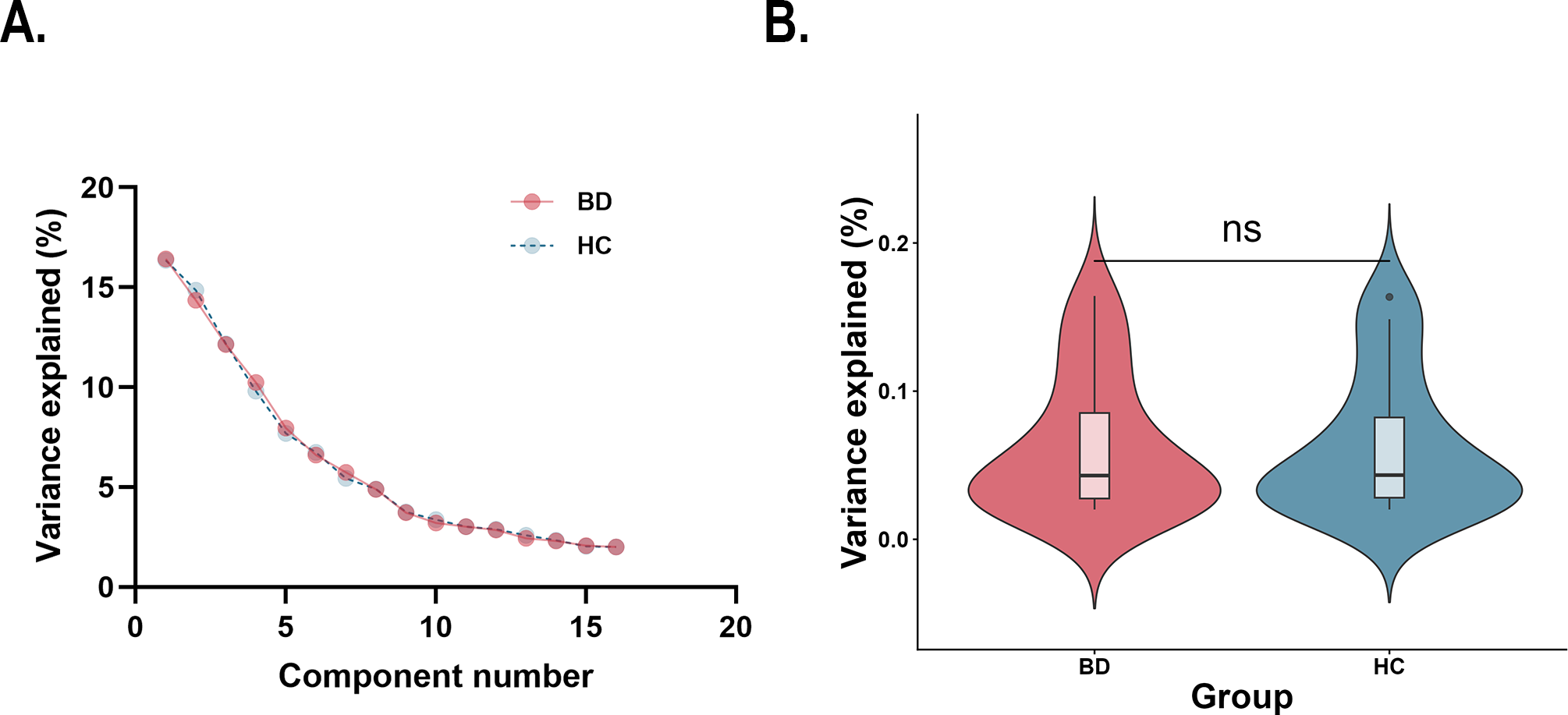


**Figure S1.** Variance of the MIND network explained by the gradient components.

**A.** Scree plot showing the variance explained by the gradient components of the MIND network. The gradient components were derived using the diffusion embedding algorithm. Healthy controls are shown in light blue, and BD patients are shown in pink. **B.** Differences in variance explained of the principal MIND gradient between BD and HC. Abbreviations: BD, bipolar disorder; HC, healthy controls; MIND, Morphometric Inverse Divergence; ns, not significant.


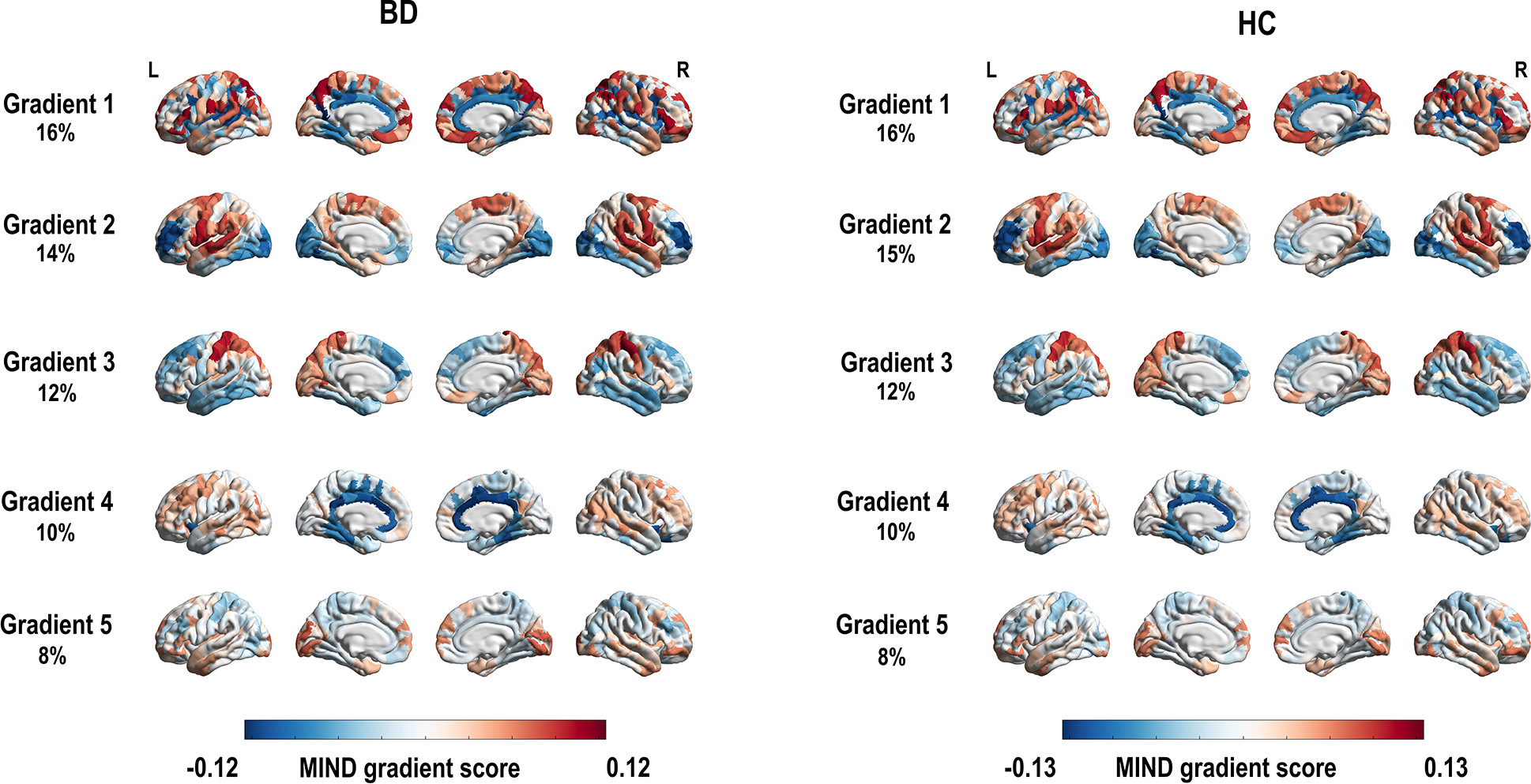


**Figure S2.** The spatial pattern of the 1-5 MIND gradient components.

The 1-5 gradient components were derived using the diffusion embedding algorithm. The percentages represent variance explained by the gradient components of the MIND network. Abbreviations: BD, bipolar disorder; HC, healthy controls; R, right; L, left; MIND, Morphometric Inverse Divergence.


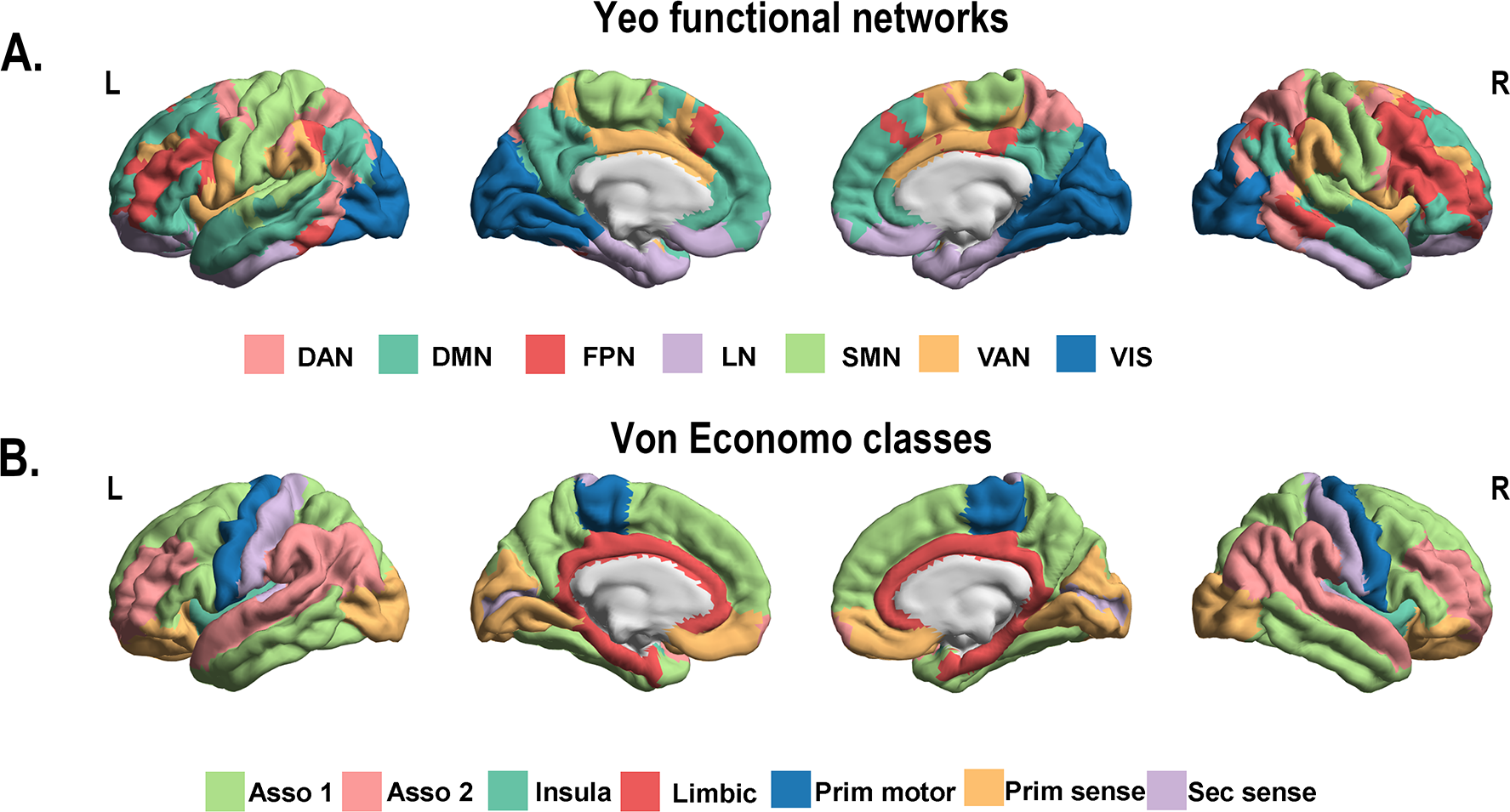


**Figure S3.** The spatial maps of Yeo functional networks and von Economo classes.

**A.** The spatial maps of Yeo functional networks. **B.** The spatial maps of von Economo classes. Abbreviations: Asso1, association cortex1; Asso2, association cortex2; DAN, dorsal attention network; DMN, default mode network; FPN, fronto-parietal network; Insula, insular cortex; L, left; Limbic, limbic regions; LN, limbic network; Prim motor, primary motor cortex; Prim sens, primary sensory cortex; R, right; Sec sens, second sensory cortex; SMN, somato-motor network; VAN, ventral attention network; VIS, visual network.


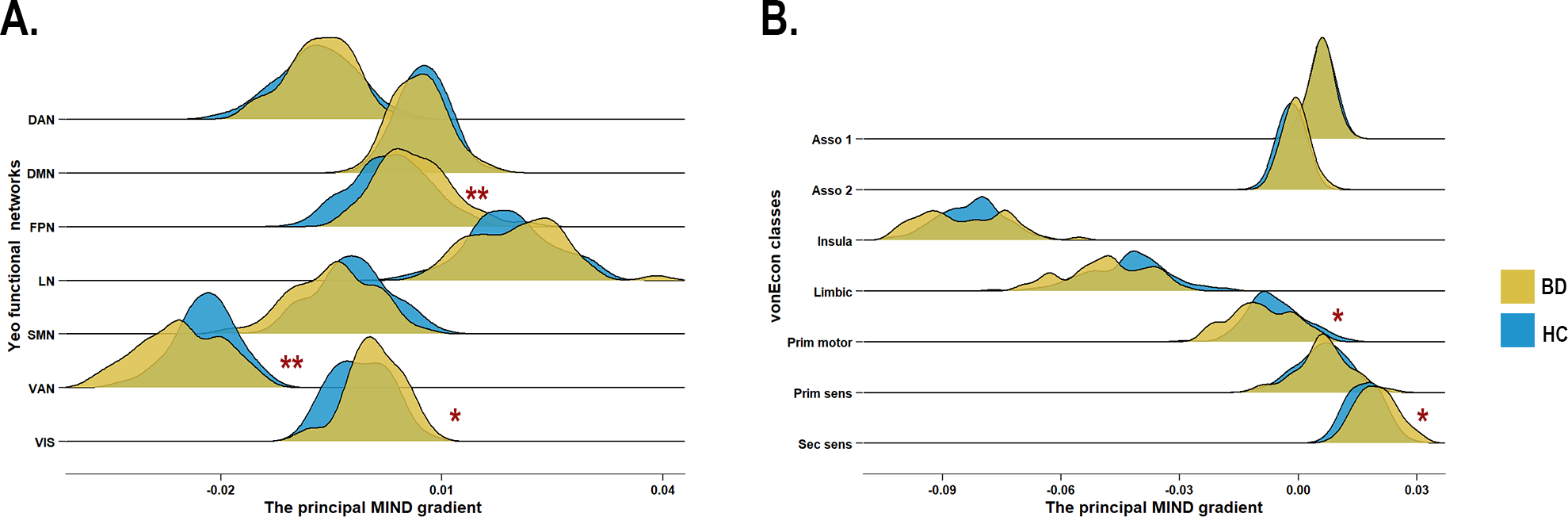


**Figure S4.** Distribution of the principal MIND gradient in BD and HC.

**A.** Distribution of the principal MIND gradient in BD and HC based on Yeo functional networks. **B.** Distribution of the principal MIND gradient in BD and HC based on Von Economo classes. All *P* values survived after BH-FDR correction with *P* < 0.05. ^*^Indicates that the *P* value < 0.05, and ^**^ indicates that the *P* value < 0.01. Abbreviations: Asso1, association cortex1; Asso2, association cortex2; BD, bipolar disorder; BH-FDR, Benjamini-Hochberg false discovery rate; DAN, dorsal attention network; DMN, default mode network; FPN, fronto-parietal network; HC, healthy controls; Insula, insular cortex; Limbic, limbic regions; LN, limbic network; MIND, Morphometric Inverse Divergence; Prim motor, primary motor cortex; Prim sens, primary sensory cortex; Sec sens, second sensory cortex; SMN, somato-motor network; VAN, ventral attention network; VIS, visual network.


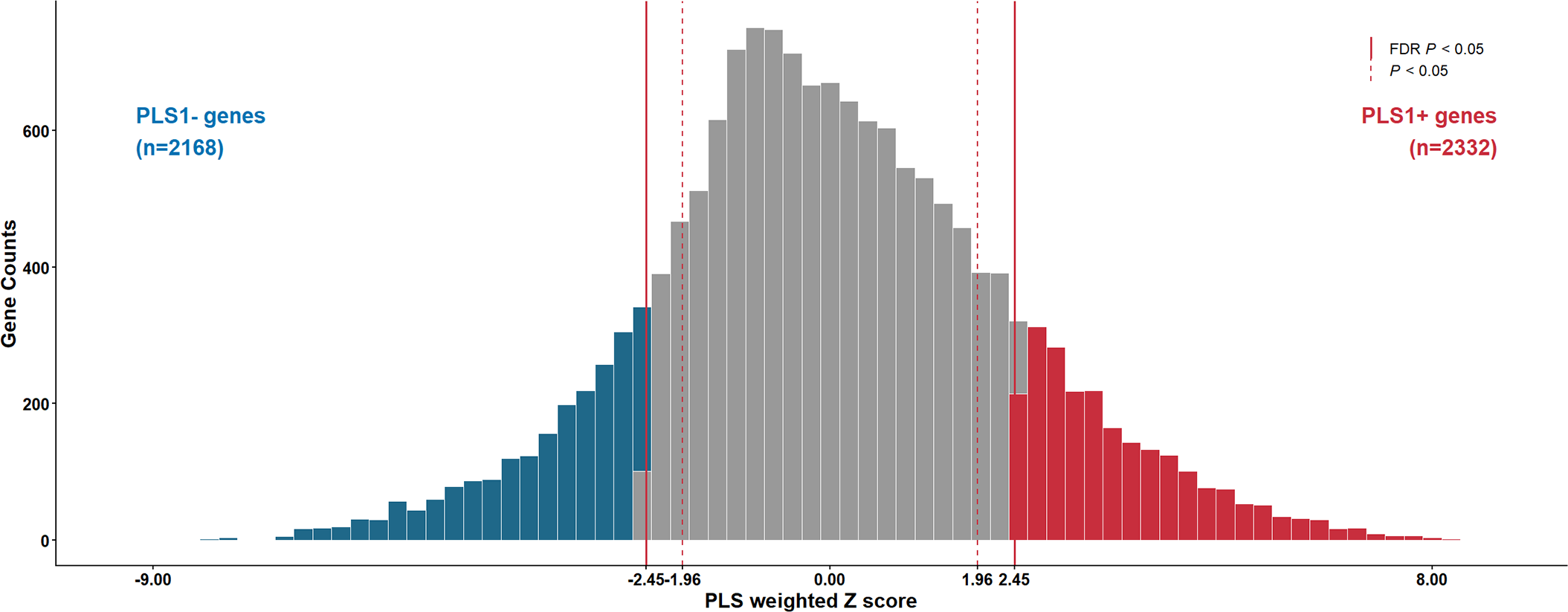


**Figure S5.** Distribution of PLS1 Weighted *Z-*score.

A total of 2332 PLS1+ genes (*Z*-score > 2.45, FDR *P* value < 0.05) and 2168 PLS1- genes (*Z*-score < − 2.45, FDR *P* value < 0.05) were identified by ranked *Z*-score. All *P* values survived after BH-FDR correction with *P* < 0.05. Abbreviations: BH-FDR, Benjamini-Hochberg false discovery rate; PLS, Partial Least Squares.


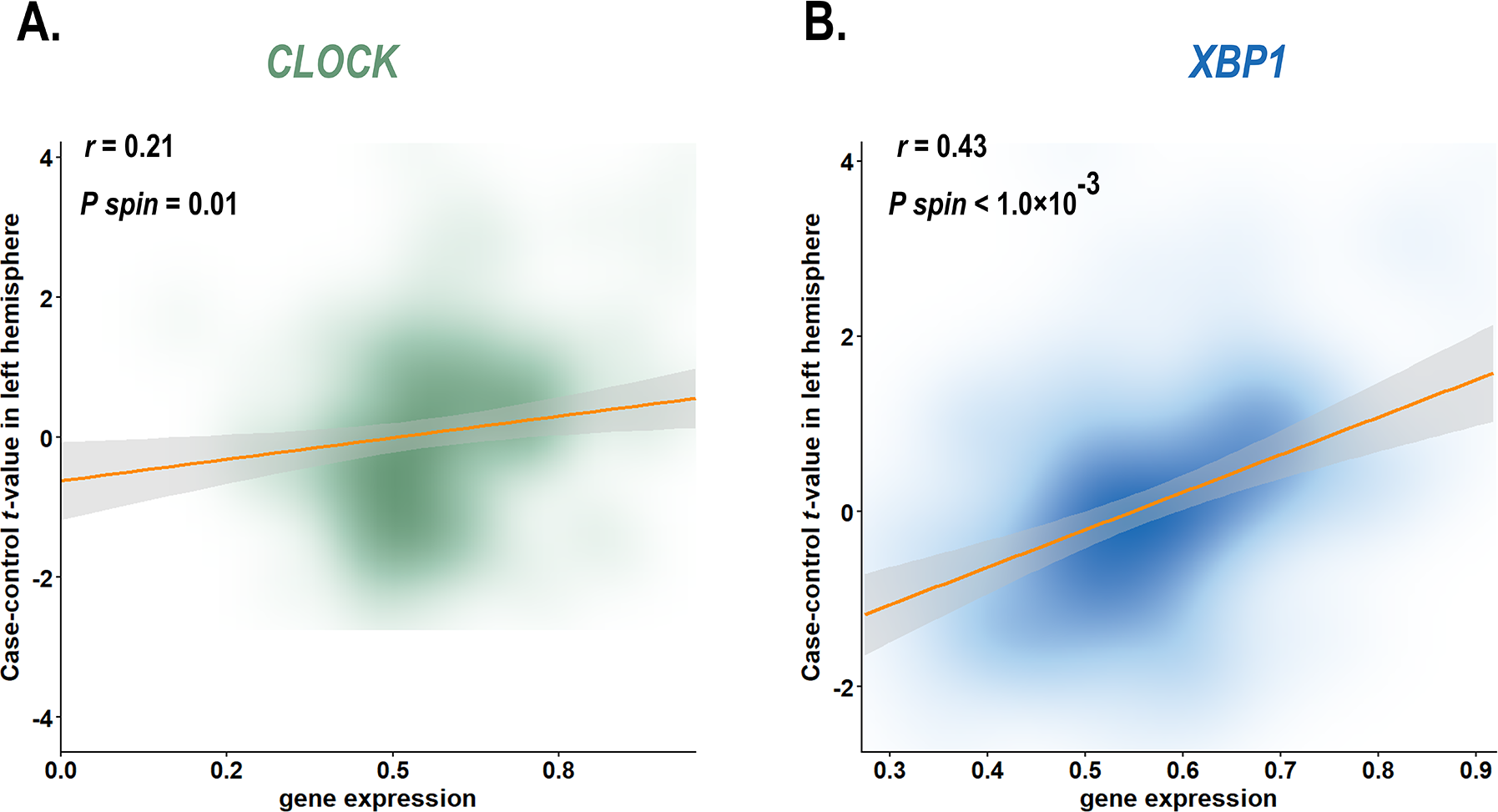


**Figure S6.** The significant correlations between the two BD-related genes from the AHBA database and the case-control *t*-map.

Two genes were positively correlated with regional changes in the principal MIND gradient. The gray band indicates the 95% conﬁdence interval. All *P* values were derived from spin tests and adjusted by the BH-FDR method. Abbreviations: AHBA, Allen Human Brain Atlas; BD, bipolar disorder; BH-FDR, Benjamini-Hochberg false discovery rate; *CLOCK*, Circadian Locomotor Output Cycles Kaput; MIND, Morphometric Inverse Divergence; *XBP1*, X-box Binding Protein 1.


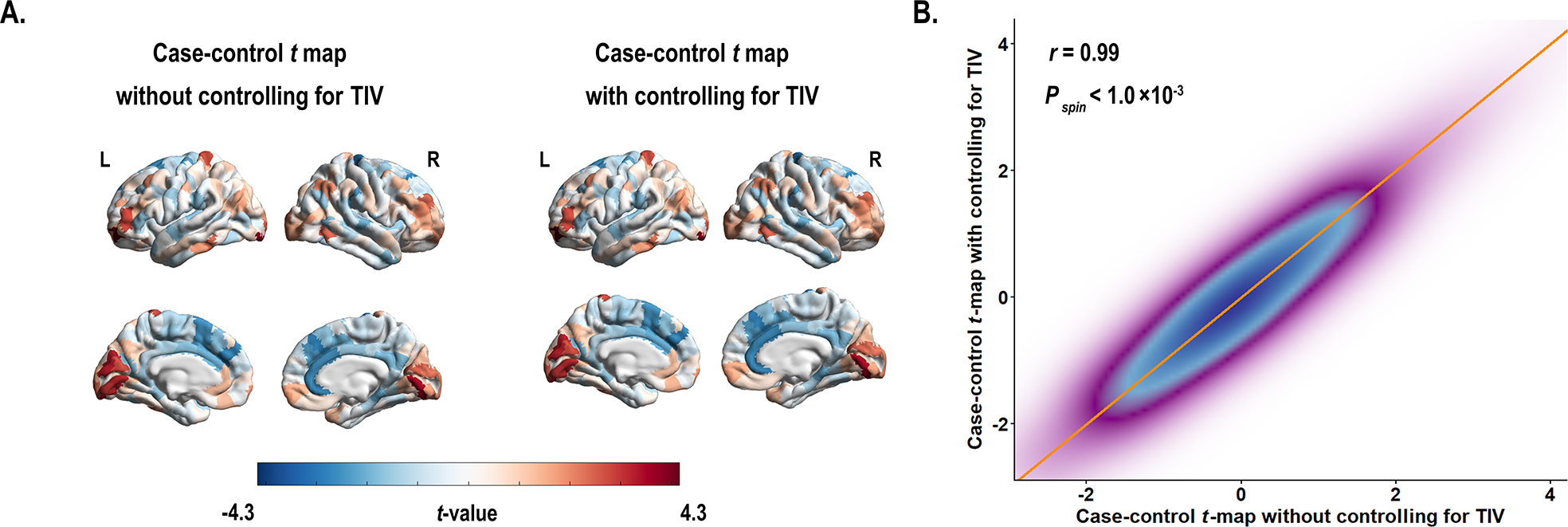


**Figure S7.** TIV effect on case-control differences.

**A.** The case-control *t* map with without controlling for TIV. **B.** The case-control *t* map with controlling for TIV. **C.** The results of spatial correlation analysis between the principal MIND gradient without and with controlling for TIV. *P* value was calculated based on spin test. Abbreviations: L, left; MIND, Morphometric Inverse Divergence; R, right; TIV, total intracranial volume.


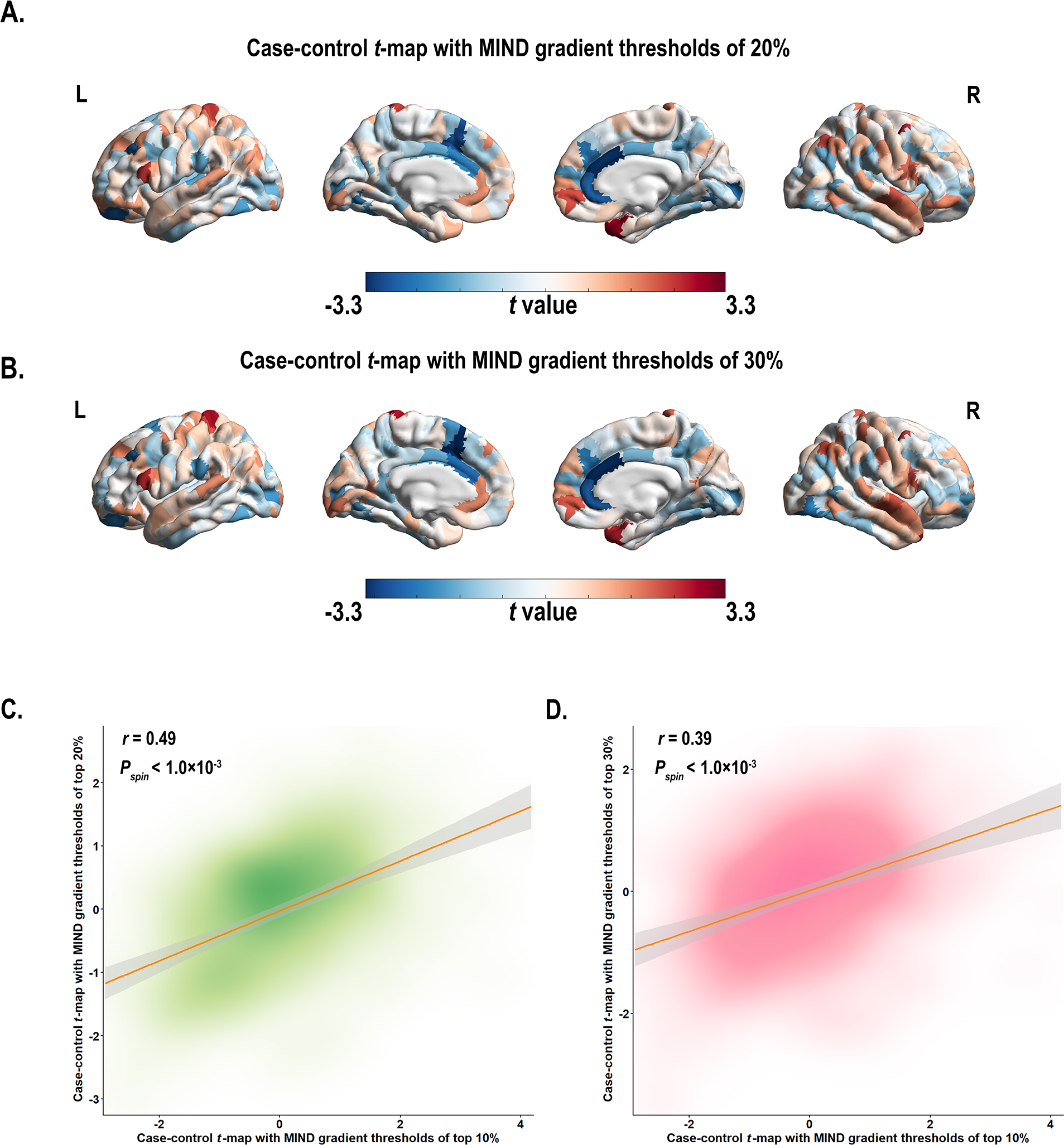


**Figure S8.** The effect of different thresholds (top 20% and 30%) on the principal MIND gradient’s manifestation in case-control differences.

**A.** The case-control *t* map with the principal MIND gradient thresholds of top 20%. **B.** The case-control *t* map with the principal MIND gradient thresholds of top 30%. **C.** The results of spatial correlation analysis between the principal MIND gradient with thresholds of top 10% and with the principal MIND gradient with thresholds of top 20%. **D.** The results of spatial correlation analysis between the principal MIND gradient with thresholds of top 10% and with the principal MIND gradient with thresholds of top 30%. The gray band indicates the 95% conﬁdence interval. All *P* values were calculated based on spin test. Abbreviations: L, left; MIND, Morphometric Inverse Divergence; R, right.


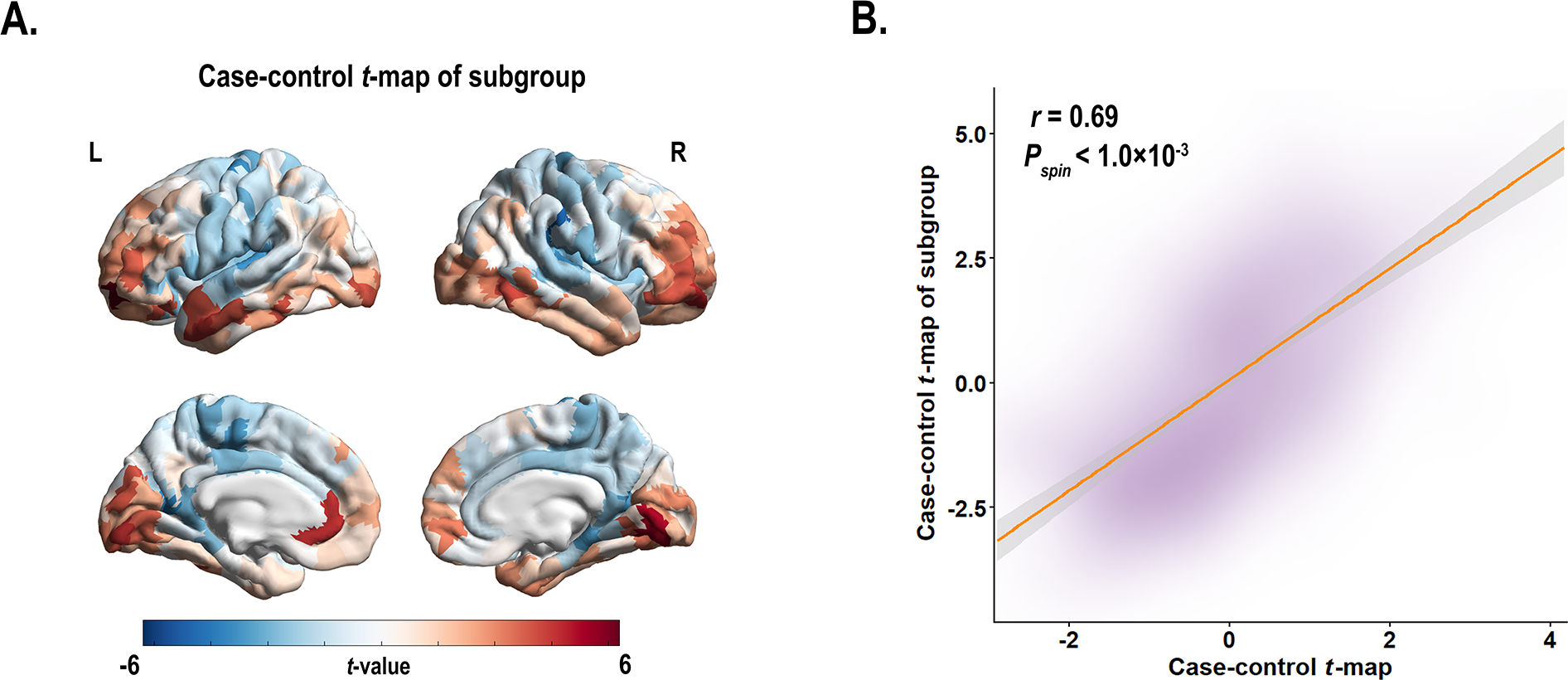


**Figure S9.** Sample matching effect on case-control differences.

**A.** Case-control *t*-map of the principal MIND gradient in the matched subgroup **B.** The results of spatial correlation analysis between the principal MIND gradient and the principal MIND gradient from the matched subgroup. The gray band indicates the 95% conﬁdence interval. *P* value was calculated based on spin test. Abbreviations: L, left; MIND, Morphometric Inverse Divergence; R, right.


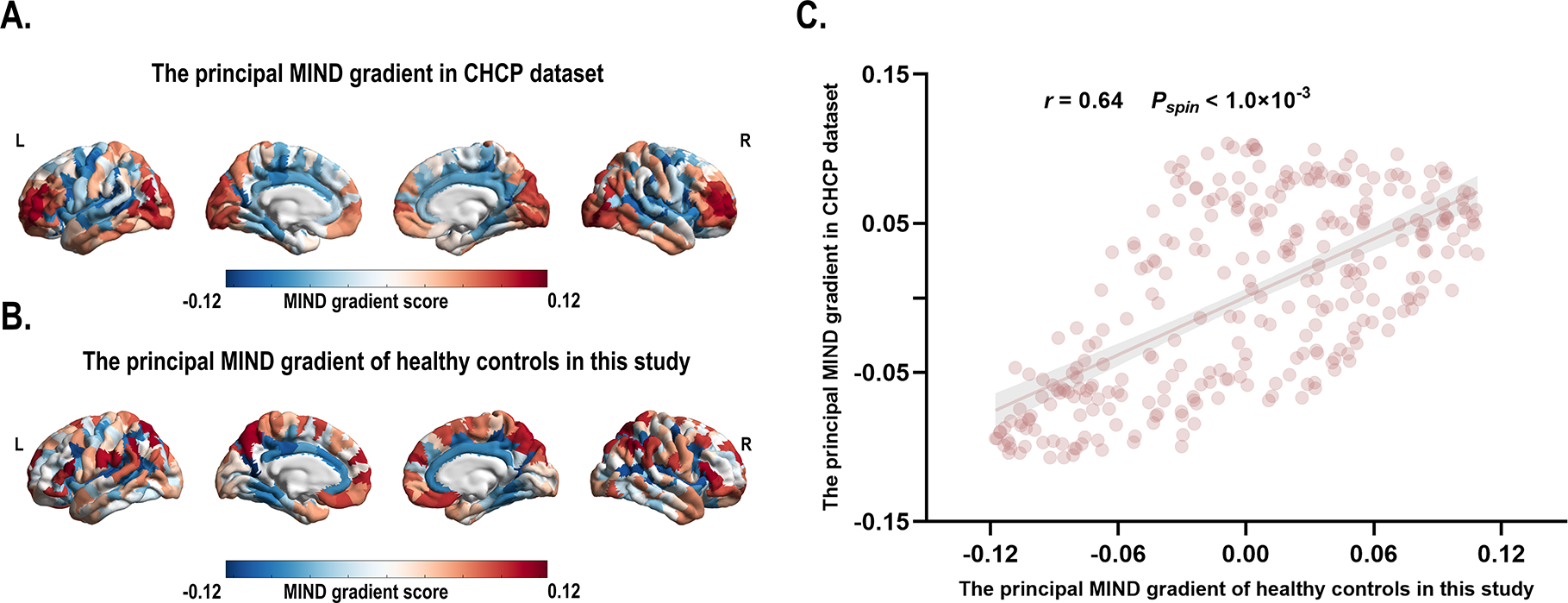


**Figure S10.** The replicable principal gradient of MIND in healthy controls.

**A.** The principal MIND gradient in the CHCP dataset (Ge et al., 2023). **B.** The principal MIND gradient of health controls in this study. **C.** The results of spatial correlation analysis between two datasets. The gray band indicates the 95% conﬁdence interval. *P* value was calculated based on spin test. Abbreviations: CHCP, Chinese Human Connectome Project; L, left; MIND, Morphometric Inverse Divergence; R, right.


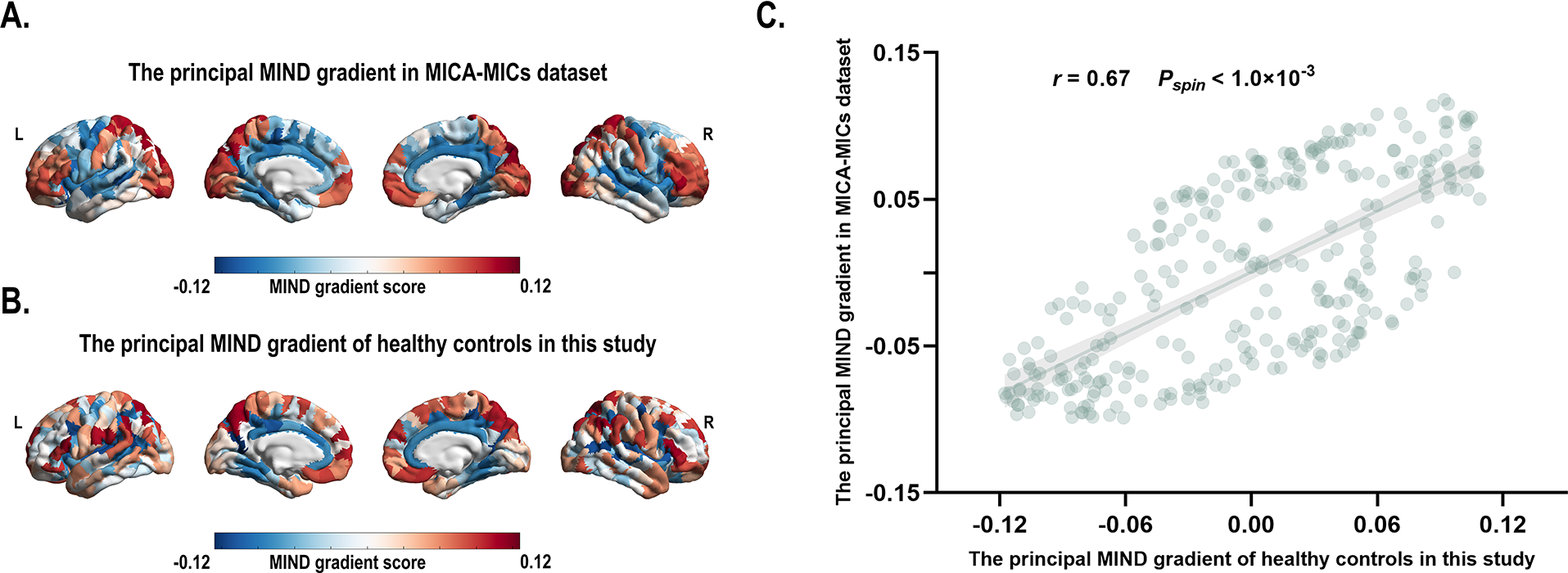


**Figure S11.** The replicable principal gradient of MIND in healthy controls.

**A.** The principal MIND gradient in the MICA-MICs dataset (Royer et al., 2022). **B.** The principal MIND gradient of health controls in this study. **C.** The results of spatial correlation analysis between two datasets. The gray band indicates the 95% conﬁdence interval. *P* value was calculated based on spin test. Abbreviations: L, left; MIND, Morphometric Inverse Divergence; MICA-MICs, Multimodal Imaging and Connectome Analysis-Microstructure-Informed Connectomics; R, right.


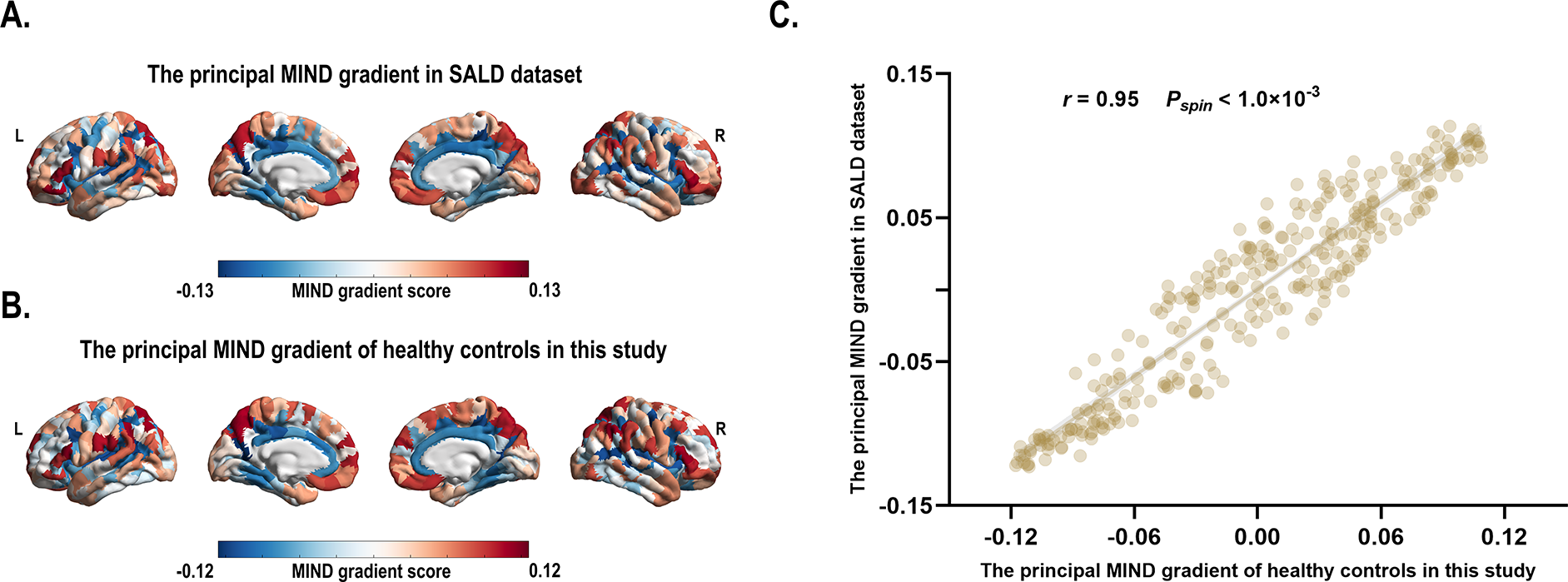


**Figure S12.** The replicable principal gradient of MIND in healthy controls.

**A.** The principal MIND gradient in the SALD dataset (participants aged > 60 years) (Wei et al., 2018). **B.** The principal MIND gradient of health controls in this study. **C.** The results of spatial correlation analysis between two datasets. The gray band indicates the 95% conﬁdence interval. *P* value was calculated based on spin test. Abbreviations: L, left; MIND, Morphometric Inverse Divergence; MICA-MICs, Multimodal Imaging and Connectome Analysis-Microstructure-Informed Connectomics; SALD, Southwest University Adult Lifespan Dataset; R, right.


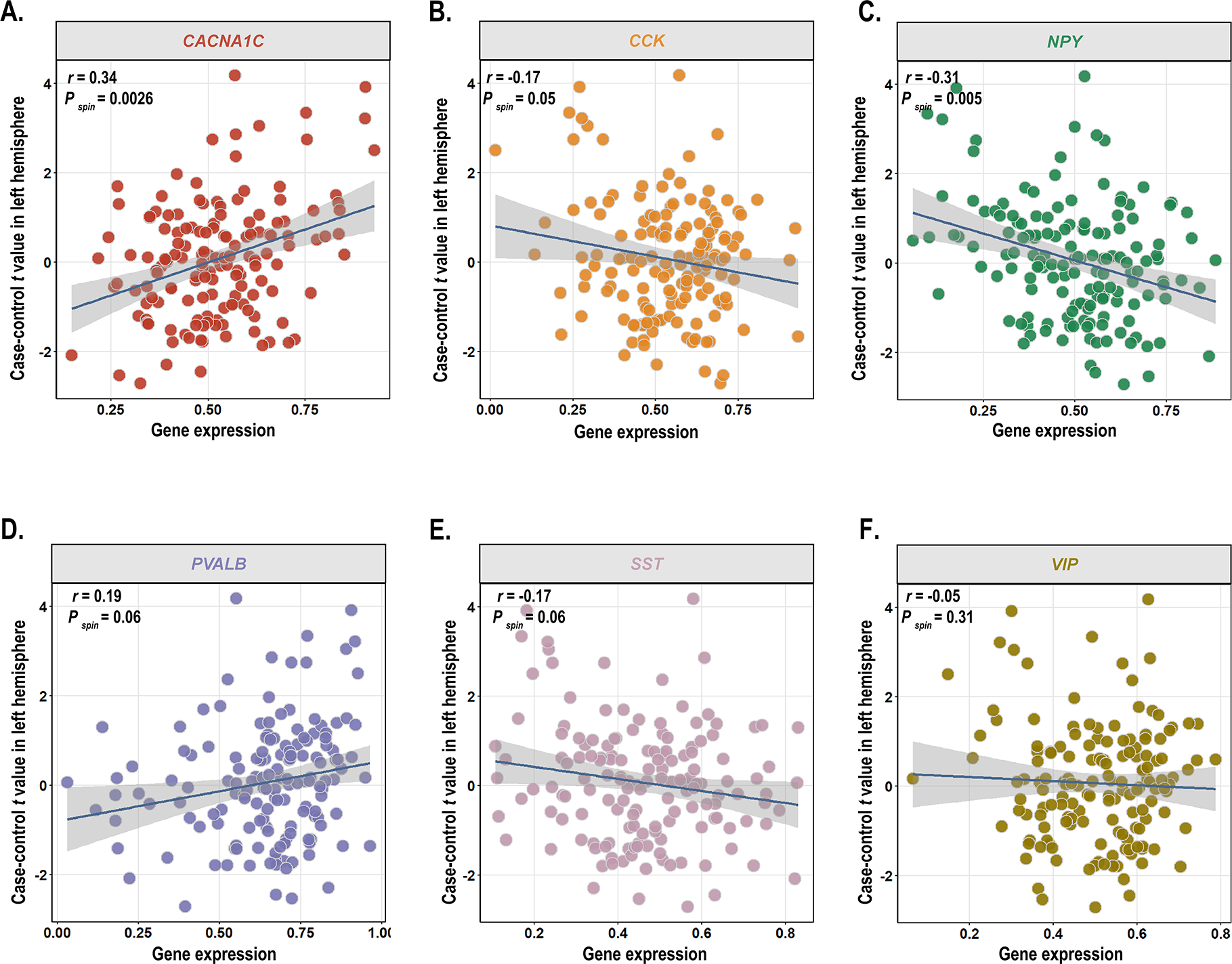


**Figure S13.** Significant correlations of *CACNA1C* and *SST*-related gene expression with the case-control *t*-map.

**A.** Scatter plot showing the associations between gene expression of CACNA1C gene and the case-control *t*-map. **B-F.** Scatter plots showing the associations between gene expression of SST-related genes and the case-control *t*-maps. Three gene markers are positively correlated with regional changes in the principal MIND gradient. Four gene markers are negatively correlated with regional changes in the principal MIND gradient. The gray band indicates the 95% conﬁdence interval. All *P* values were derived from spin tests and adjusted by the BH-FDR method. Abbreviations: BH-FDR, Benjamini-Hochberg false discovery rate; *CACNA1C*, Calcium Voltage-Gated Channel Subunit Alpha 1C; *CCK*, Cholecystokinin; MIND, Morphometric Inverse Divergence; *NPY*, Neuropeptide Y; *PVALB*, Parvalbumin; *SST*, somatostatin; *VIP*, Vasoactive Intestinal Peptide.

**Supplementary Tables and Figures References**

Aghourian, M., Legault-Denis, C., Soucy, J. P., Rosa-Neto, P., Gauthier, S., Kostikov, A., . . . Bédard, M. A. (2017). Quantification of brain cholinergic denervation in Alzheimer's disease using PET imaging with [(18)F]-FEOBV. *Mol Psychiatry, 22*(11), 1531-1538. doi:10.1038/mp.2017.183

Bedard, M. A., Aghourian, M., Legault-Denis, C., Postuma, R. B., Soucy, J. P., Gagnon, J. F., . . . Montplaisir, J. (2019). Brain cholinergic alterations in idiopathic REM sleep behaviour disorder: a PET imaging study with (18)F-FEOBV. *Sleep Med, 58*, 35-41. doi:10.1016/j.sleep.2018.12.020

Beliveau, V., Ganz, M., Feng, L., Ozenne, B., Højgaard, L., Fisher, P. M., . . . Knudsen, G. M. (2017). A High-Resolution In Vivo Atlas of the Human Brain's Serotonin System. *J Neurosci, 37*(1), 120-128. doi:10.1523/jneurosci.2830-16.2016

Ding, Y. S., Singhal, T., Planeta-Wilson, B., Gallezot, J. D., Nabulsi, N., Labaree, D., . . . Malison, R. T. (2010). PET imaging of the effects of age and cocaine on the norepinephrine transporter in the human brain using (S,S)-[(11)C]O-methylreboxetine and HRRT. *Synapse, 64*(1), 30-38. doi:10.1002/syn.20696

DuBois, J. M., Rousset, O. G., Rowley, J., Porras-Betancourt, M., Reader, A. J., Labbe, A., . . . Kobayashi, E. (2016). Characterization of age/sex and the regional distribution of mGluR5 availability in the healthy human brain measured by high-resolution [(11)C]ABP688 PET. *Eur J Nucl Med Mol Imaging, 43*(1), 152-162. doi:10.1007/s00259-015-3167-6

Dukart, J., Holiga, Š., Chatham, C., Hawkins, P., Forsyth, A., McMillan, R., . . . Sambataro, F. (2018). Cerebral blood flow predicts differential neurotransmitter activity. *Sci Rep, 8*(1), 4074. doi:10.1038/s41598-018-22444-0

Gallezot, J. D., Nabulsi, N., Neumeister, A., Planeta-Wilson, B., Williams, W. A., Singhal, T., . . . Carson, R. E. (2010). Kinetic modeling of the serotonin 5-HT(1B) receptor radioligand [(11)C]P943 in humans. *J Cereb Blood Flow Metab, 30*(1), 196-210. doi:10.1038/jcbfm.2009.195

Gallezot, J. D., Planeta, B., Nabulsi, N., Palumbo, D., Li, X., Liu, J., . . . Carson, R. E. (2017). Determination of receptor occupancy in the presence of mass dose: [(11)C]GSK189254 PET imaging of histamine H(3) receptor occupancy by PF-03654746. *J Cereb Blood Flow Metab, 37*(3), 1095-1107. doi:10.1177/0271678x16650697

Galovic, M., Al-Diwani, A., Vivekananda, U., Torrealdea, F., Erlandsson, K., Fryer, T. D., . . . investigators, f. t. N. (2021). In vivo NMDA receptor function in people with NMDA receptor antibody encephalitis. *medRxiv*, 2021.2012.2004.21267226. doi:10.1101/2021.12.04.21267226

Ge, J., Yang, G., Han, M., Zhou, S., Men, W., Qin, L., . . . Rao, H. (2023). Increasing diversity in connectomics with the Chinese Human Connectome Project. *Nature Neuroscience, 26*(1), 163-172.

Hillmer, A. T., Esterlis, I., Gallezot, J. D., Bois, F., Zheng, M. Q., Nabulsi, N., . . . Cosgrove, K. P. (2016). Imaging of cerebral α4β2* nicotinic acetylcholine receptors with (-)-[(18)F]Flubatine PET: Implementation of bolus plus constant infusion and sensitivity to acetylcholine in human brain. *Neuroimage, 141*, 71-80. doi:10.1016/j.neuroimage.2016.07.026

Kaller, S., Rullmann, M., Patt, M., Becker, G. A., Luthardt, J., Girbardt, J., . . . Sabri, O. (2017). Test-retest measurements of dopamine D(1)-type receptors using simultaneous PET/MRI imaging. *Eur J Nucl Med Mol Imaging, 44*(6), 1025-1032. doi:10.1007/s00259-017-3645-0

Kantonen, T., Karjalainen, T., Isojärvi, J., Nuutila, P., Tuisku, J., Rinne, J., . . . Nummenmaa, L. (2020). Interindividual variability and lateralization of μ-opioid receptors in the human brain. *Neuroimage, 217*, 116922. doi:10.1016/j.neuroimage.2020.116922

Naganawa, M., Nabulsi, N., Henry, S., Matuskey, D., Lin, S. F., Slieker, L., . . . Huang, Y. (2021). First-in-Human Assessment of (11)C-LSN3172176, an M1 Muscarinic Acetylcholine Receptor PET Radiotracer. *J Nucl Med, 62*(4), 553-560. doi:10.2967/jnumed.120.246967

Nørgaard, M., Beliveau, V., Ganz, M., Svarer, C., Pinborg, L. H., Keller, S. H., . . . Knudsen, G. M. (2021). A high-resolution in vivo atlas of the human brain's benzodiazepine binding site of GABA(A) receptors. *Neuroimage, 232*, 117878. doi:10.1016/j.neuroimage.2021.117878

Normandin, M. D., Zheng, M. Q., Lin, K. S., Mason, N. S., Lin, S. F., Ropchan, J., . . . Huang, Y. (2015). Imaging the cannabinoid CB1 receptor in humans with [11C]OMAR: assessment of kinetic analysis methods, test-retest reproducibility, and gender differences. *J Cereb Blood Flow Metab, 35*(8), 1313-1322. doi:10.1038/jcbfm.2015.46

Poldrack, R. A., Kittur, A., Kalar, D., Miller, E., Seppa, C., Gil, Y., . . . Bilder, R. M. (2011). The cognitive atlas: toward a knowledge foundation for cognitive neuroscience. *Front Neuroinform, 5*, 17. doi:10.3389/fninf.2011.00017

Radhakrishnan, R., Matuskey, D., Nabulsi, N., Gaiser, E., Gallezot, J. D., Henry, S., . . . D'Souza, D. C. (2020). In vivo 5-HT(6) and 5-HT(2A) receptor availability in antipsychotic treated schizophrenia patients vs. unmedicated healthy humans measured with [(11)C]GSK215083 PET. *Psychiatry Res Neuroimaging, 295*, 111007. doi:10.1016/j.pscychresns.2019.111007

Radhakrishnan, R., Nabulsi, N., Gaiser, E., Gallezot, J. D., Henry, S., Planeta, B., . . . Matuskey, D. (2018). Age-Related Change in 5-HT(6) Receptor Availability in Healthy Male Volunteers Measured with (11)C-GSK215083 PET. *J Nucl Med, 59*(9), 1445-1450. doi:10.2967/jnumed.117.206516

Royer, J., Rodríguez-Cruces, R., Tavakol, S., Larivière, S., Herholz, P., Li, Q., . . . Bernhardt, B. C. (2022). An Open MRI Dataset For Multiscale Neuroscience. *Sci Data, 9*(1), 569. doi:10.1038/s41597-022-01682-y

Savli, M., Bauer, A., Mitterhauser, M., Ding, Y. S., Hahn, A., Kroll, T., . . . Lanzenberger, R. (2012). Normative database of the serotonergic system in healthy subjects using multi-tracer PET. *Neuroimage, 63*(1), 447-459. doi:10.1016/j.neuroimage.2012.07.001

Smart, K., Cox, S. M. L., Scala, S. G., Tippler, M., Jaworska, N., Boivin, M., . . . Leyton, M. (2019). Sex differences in [(11)C]ABP688 binding: a positron emission tomography study of mGlu5 receptors. *Eur J Nucl Med Mol Imaging, 46*(5), 1179-1183. doi:10.1007/s00259-018-4252-4

Smith, C. T., Crawford, J. L., Dang, L. C., Seaman, K. L., San Juan, M. D., Vijay, A., . . . Samanez-Larkin, G. R. (2019). Partial-volume correction increases estimated dopamine D2-like receptor binding potential and reduces adult age differences. *J Cereb Blood Flow Metab, 39*(5), 822-833. doi:10.1177/0271678x17737693

Wei, D., Zhuang, K., Ai, L., Chen, Q., Yang, W., Liu, W., . . . Qiu, J. (2018). Structural and functional brain scans from the cross-sectional Southwest University adult lifespan dataset. *Sci Data, 5*, 180134. doi:10.1038/sdata.2018.134

Yarkoni, T., Poldrack, R. A., Nichols, T. E., Van Essen, D. C., & Wager, T. D. (2011). Large-scale automated synthesis of human functional neuroimaging data. *Nature Methods, 8*(8), 665-670. doi:10.1038/nmeth.1635
